# Organelle genome architecture of *Salvia plebeia* reveals mitochondrial recombination and evolutionary dynamics

**DOI:** 10.3389/fpls.2026.1865234

**Published:** 2026-07-09

**Authors:** Xun Gong, Keming Zhu, Emmanuel Fleming, Shaoshuai Yu, Min Tang

**Affiliations:** 1 Department of Rheumatology & Immunology, Affiliated Hospital of Jiangsu University, Zhenjiang, Jiangsu, China; 2 School of Life Sciences, Jiangsu University, Zhenjiang, Jiangsu, China; 3 Department of Pharmacy, Affiliated People’s Hospital of Jiangsu University, Zhenjiang, Jiangsu, China

**Keywords:** Lamiaceae, mitochondrial genome evolution, organelle genome, phylogenetic analysis, repeat-mediated recombination, Salvia plebeian

## Abstract

**Introduction:**

*Salvia plebeia* is a medicinal plant in Lamiaceae, but its organelle genome architecture and evolutionary dynamics remain insufficiently understood, particularly for the mitochondrial genome.

**Methods:**

We assembled and analyzed the mitochondrial and chloroplast genomes of *S. plebeia* using PacBio HiFi long-read sequencing. Genome assembly, annotation, and correction were performed using PMAT2, Minimap2, NextPolish, and GeSeq. Repetitive elements were identified using SSR, tandem repeat, and dispersed repeat analyses. Repeat-mediated recombination was validated using long-read mapping and PCR. Comparative chloroplast genomics and phylogenetic analyses were conducted using mVISTA, MUMmer4, and IQ-TREE2. RNA-editing sites were predicted using PREPACT, and codon usage bias was analyzed using CodonW.

**Results:**

The mitogenome is a 444,036 bp circular molecule encoding 58 genes, while the chloroplast genome is 151,062 bp with a typical quadripartite structure. A total of 135 SSRs, 331 dispersed repeats, and 25 mitochondrial plastid DNA transfer (MTPT) regions were identified. Six repeat pairs were experimentally supported to mediate homologous recombination, indicating alternative mitochondrial conformations. RNA-editing analysis identified 587 C-to-U sites. Phylogenetic analyses placed S. plebeia within the *Salvia* lineage, and plastomes showed high conservation, whereas the mitogenome exhibited extensive structural rearrangements.

**Discussion:**

These findings highlight a contrast between conserved chloroplast genomes and highly dynamic mitochondrial genomes in *S. plebeia*, driven by repeat-mediated recombination and RNA editing, providing new insights into organelle genome evolution in Lamiaceae.

## Introduction

*Salvia* L. is the largest genus in the family Lamiaceae and comprises more than 1, 000 species with substantial medicinal, culinary, ornamental, and ecological value. Representative members, including *Salvia miltiorrhiza*, *Salvia officinalis*, *Salvia rosmarinus*, and *Salvia plebeia*, have long been used as medicinal or aromatic plants and have attracted increasing attention in phytochemistry, pharmacology, and plant genomics (Liang et al., 2020; Dai et al., 2023). *S. plebeia* R. Br. is an annual or biennial herb widely distributed across East Asia and other temperate to subtropical regions (Xiong et al., 2019; Kim et al., 2021; Liang et al., 2021). In traditional medicine, this species has been used for the treatment of inflammatory disorders, respiratory diseases, hepatitis, nephritis, and respiratory infections (Meng et al., 2022; Kim et al., 2023; Dai et al., 2025). Phytochemical investigations have identified flavonoids, phenolic acids, terpenoids, and volatile oils as its major bioactive constituents, among which rosmarinic acid, homoplantaginin, luteolin derivatives, and related phenolic compounds contribute to its anti-inflammatory, antioxidant, antimicrobial, hepatoprotective, and immunomodulatory activities (Zhang et al., 2015; Zou et al., 2018; Kim et al., 2021).

Recent genomic studies have substantially advanced the understanding of the nuclear genome and specialized metabolism of *S. plebeian* (Dai et al., 2025). A chromosome-scale nuclear genome assembly has provided important insights into its genome organization and the biosynthesis and regulation of rosmarinic acid, including the functional characterization of SpRAS, SpCYP98A enzymes, and the transcription factor SpbHLH54. These findings establish a valuable nuclear genomic and metabolic framework for *S. plebeia*. However, despite these advances, the organelle genomes of *S. plebeia*, especially the mitochondrial genome (mitogenome), remain insufficiently characterized, limiting a more complete understanding of its genome architecture and evolutionary features.

Plant mitogenomes are highly variable in size and structure and often contain abundant repeats, plastid-derived fragments, and rearranged genomic regions (Liu et al., 2025; Wang et al., 2025; Yu et al., 2025; Zhou et al., 2025; Qin et al., 2026). Repeat-mediated homologous recombination, particularly involving forward and palindromic repeats, can generate alternative mitochondrial conformations and is considered a major force shaping mitogenome architecture. In addition, codon usage bias, RNA editing, gene conservation, collinearity, and nonsynonymous substitution rates/synonymous substitution rates (Ka/Ks) patterns provide useful information for evaluating functional constraints and lineage-specific evolutionary dynamics. Therefore, systematic characterization of these features is essential for understanding mitogenome evolution in *S. plebeia* and related Lamiaceae species.

In this study, the mitochondrial and chloroplast genomes (cpgenome) of *S. plebeia* were assembled and analyzed using PacBio HiFi long-read sequencing. The study aimed to characterize the general features of the *S. plebeia* mitogenome, identify repetitive elements and repeat-mediated recombination patterns, evaluate codon usage and RNA-editing profiles, compare cpgenome architecture, and resolve the phylogenetic and comparative mitogenomic relationships of *S. plebeia* within Lamiaceae. These analyses provide new insights into organelle genome architecture, mitochondrial recombination, and evolutionary dynamics in *S. plebeia*, thereby complementing existing nuclear genomic studies and expanding genomic resources for medicinal plants in the genus *Salvia*.

## Materials and methods

### Collection of plant materials, library preparation, and sequencing

Fresh leaves of *S. plebeian* were collected from Jiangsu University, Jiangsu Province, China (coordinates: 32°12′N, 119°30′E). The collected plant materials were then authenticated by two botanists from the School of Life Sciences at Jiangsu University, confirming the species identity as *S. plebeia*. After confirmation, leaves were washed with DEPC-treated water and immediately frozen at −80 °C. Genomic DNA was extracted using the CTAB method, and its quality was assessed via 0.75% agarose gel electrophoresis, a Qubit 3.0 Fluorometer (Life Technologies, USA), and a NanoDrop One spectrophotometer (Thermo Fisher Scientific, USA) (Yao et al., 2022). For high-fidelity (HiFi) long-read sequencing, SMRTbell libraries were prepared using the SMRTbell Prep Kit 3.0 (Pacific Biosciences, Menlo Park, CA, USA) and sequenced on the PacBio Sequel II system with Circular Consensus Sequencing (CCS) mode. High-quality HiFi reads were generated through CCS.

### Methodological approach for DNA barcoding in species identification

To establish a robust molecular identification framework, we employed a DNA barcode approach targeting the genes *rbcL* and *matK*, which were universally recognized as core barcoding markers due to their high discrimination power and sequence variability among species for plant species discrimination (Zhu et al., 2022; Yu et al., 2026). For species verification, all barcode sequences were queried against the Barcode of Life Data Systems (BOLD) database (https://www.boldsystems.org) using the BOLD Identification Engine (Sonet et al., 2013). Taxonomic assignment was determined based on the highest sequence identity to vouchered reference specimens. To further validate the species identity at the organelle-genome level, the assembled cpgenome was compared with the publicly available *S. plebeia* cpgenome from NCBI (NC_050929.1). BLASTn (Camacho et al., 2009) was used for whole-plastome comparison, and sequence identity, query coverage, and reference coverage were calculated based on the alignment results.

### Assembly and annotation of organelle genomes

The mitogenome of *S. plebeia* was assembled using PacBio HiFi long-read sequencing data. Initial assembly was performed with PMAT2 software (v2.0.2, parameter: -g 5G) to generate raw contigs (Bi et al., 2024). Assembly graphs (GFA files) were visualized and manually adjusted using Bandage (v0.8.1) to remove non-mitochondrial sequences and refine repetitive regions (Wick et al., 2015). To improve accuracy, HiFi reads were mapped to the preliminary assembly using Minimap2 (Li, 2018), followed by polishing with NextPolish to correct base errors and resolve structural ambiguities (Hu et al., 2020), yielding the final mitogenome sequence. For annotation, the mitogenomes of closely related species (*Salvia rosmarinus* PP992923.1) was used as references in GeSeq (https://chlorobox.mpimp-golm.mpg.de/geseq.html) to predict protein-coding genes (PCGs) (Tillich et al., 2017). Transfer RNAs (tRNAs) and ribosomal RNAs (rRNAs) were identified using tRNAscan-SE (v2.0) and BLASTn (Chan et al., 2021), respectively. Any discrepancies in the annotation were manually resolved with Apollo (v2.5.0) (Dunn et al., 2019). The final circular mitogenome map was visualized with OGDRAW (https://chlorobox.mpimp-golm.mpg.de/OGDraw.html) (Greiner et al., 2019).

### Comprehensive analysis of repetitive elements and homologous recombination in mitogenome

Repetitive sequences in the *S. plebeia* mitogenome, including simple sequence repeats (SSRs), tandem repeats, and dispersed repeats, were systematically characterized to investigate their roles in homologous recombination. SSRs were identified using misa.pl (v2.1) with thresholds set at 10, 5, 4, 3, 3 and 3 repeat units for mono-, di-, tri-, tetra-, penta- and hexanucleotide motifs, respectively, allowing a maximum distance of 1000 bp between adjacent SSRs (Thiel et al., 2003). Tandem repeats (≥ 7 bp unit length) were detected by Tandem Repeats Finder (TRF v4.09) with parameters 2 7 7 80 10 50 500 -f -d -m (Benson, 1999), while dispersed repeats (Minimal repeat size ≥ 30 bp, Hamming distance ≤ 3) were identified using REPuter (https://bibiserv.cebitec.uni-bielefeld.de/reputer) (Kurtz et al., 2001). The distribution of these repeats was visualized via Circos (v0.69-8), revealing their genomic organization (Krzywinski et al., 2009).

To assess recombination potential, flanking sequences (1000 bp upstream/downstream) of these repeats were extracted to generate reference and recombinant templates. Two conformational models were constructed: (1) a reference conformation representing the assembled genomic arrangement and (2) a recombinant conformation simulating hypothetical rearrangements (inferred from REPuter-identified repeat pairs) mediated by repeat-driven homologous recombination. PacBio HiFi reads were aligned to both conformations using Minimap2, and reads spanning repeat-flanking junctions were extracted to evaluate structural support. Alternative mitogenome conformations were determined based on read support, and recombinant conformations supported by junction-spanning reads were considered evidence of repeat-associated structural heterogeneity.

Experimental validation was performed by designing primers (F1&R1 and F2&R2) targeting flanking regions (300 bp upstream/downstream) of candidate repeats. PCR amplification was conducted in a 25 μl reaction mixture containing 1 μl genomic DNA, 1 μl each of 8 μM forward and reverse primers, 13 μl 2× Taq PCR Master Mix, and 10 μl ddH2O. Thermocycling conditions included initial denaturation at 96 °C for 3 min, followed by 32 cycles of 94 °C (30 s), 62 °C (30 s), and 72 °C (1 min), with a final extension at 72 °C for 10 min. Amplified products were resolved by agarose gel electrophoresis and sequenced via Sanger sequencing to confirm recombination-induced structural variations.

### Assembly of cpgenome and identification of mitochondrial plastid sequences

The cpgenome of *S. plebeia* was assembled by HiFi long-read sequencing data using ptGAUL software (Zhou et al., 2023), followed by annotation through the CPGAVAS2 web server (http://47.96.249.172:16019/analyzer/annotate) (Shi et al., 2019) and visualization of the circular cpgenome map with CPGView (http://www.1kmpg.cn/cpgview/) (Liu et al., 2023). To identify mitochondrial-plastid sequence transfers (MTPTs) in *S. plebeia*, BLASTn (v2.13.0) was employed using the mitogenome as the reference database and the cpgenome as the query sequence, with an E-value threshold of 1e-6. This analysis targeted plastid-derived DNA fragments within the mitogenome, a common feature in plant mitogenomes influenced by dispersed repeats that drive homologous recombination and structural complexity. Identified MTPTs were subsequently visualized using TBtools (v2.010) (Chen et al., 2020) and functionally annotated via the GESeq platform (https://chlorobox.mpimp-golm.mpg.de/geseq.html).

### Comparative and evolutionary analysis of cpgenomes

The CPJSdraw software (v1.0.0) (Li et al., 2023) was used to detect the Large Single-Copy (LSC)/Inverted Repeat B (IRB)/Small Single-Copy (SSC)/Inverted Repeat A (IRA) boundaries between the cpgenome sequences of the four Lamiaceae plants (*Salvia rosmarinus* PP994678.1, *Salvia miltiorrhiza* OR652279.1, *Platostoma chinense* MT328397.1 and *Vitex trifolia* NC_062602.1) on the evolutionary tree that have the closest evolutionary distance to *S. plebeia* for comparative analysis. To assess structural conservation and sequence divergence within the genus Salvia, a comprehensive comparative analysis of the cpgenome was performed. The assembled cpgenome was systematically compared against four representative congeneric species: *Salvia plebeia* NC_050929.1, *Salvia rosmarinus PP994678.1*, *Salvia miltiorrhiza* OR652279.1 and *Salvia yangii* MT537168.1. Then mVISTA program (https://genome.lbl.gov/vista/mvista/submit.shtml) (Frazer et al., 2004) was employed with default parameters for global alignment visualization, utilizing the *Salvia plebeia* (NC_050929.1) as the reference sequence in the Shuffle-LAGAN alignment model. This enabled quantitative assessment of sequence identity across entire cpgenomes, including coding regions, intergenic spacers, and inverted repeat (IR) junctions. Visualization thresholds were set at 70% identity over a 100-bp sliding window to highlight divergent hotspots. Complementarily, whole-plastome alignments were generated using MUMmer4 with ‘nucmer’ alignment (Marçais et al., 2018). This identified structural rearrangements and localized inversions through dot-plot analysis.

Phylogenetic relationships were further inferred using representative cpgenome sequences from Lamiales, with particular emphasis on taxa showing close evolutionary affinity to *S. plebeia*. The taxa used for cpgenome comparison included the newly assembled *S. plebeia* cpgenome in this study, the previously published *S. plebeia* cpgenome (NC_050929.1), *Salvia rosmarinus* (PP994678.1), *Salvia miltiorrhiza* (OR652279.1), *Salvia yangii* (MT537168.1), *Platostoma chinense* (MT328397.1), and *Vitex trifolia* (NC_062602.1). Among them, *S. plebeia* (NC_050929.1), *S. rosmarinus* (PP994678.1), *S. miltiorrhiza* (OR652279.1), and *S. yangii* (MT537168.1) were mainly used for comparative analysis within the genus *Salvia*, whereas *S. rosmarinus* (PP994678.1), *S. miltiorrhiza* (OR652279.1), *P. chinense* (MT328397.1), and *V. trifolia* (NC_062602.1) were selected according to their relatively close evolutionary distances to *S. plebeia* for IR boundary comparison among related Lamiaceae species. Complete cpgenome sequences of these taxa were downloaded from GenBank, and shared chloroplast PCGs were extracted and used for phylogenetic reconstruction. Each orthologous protein-coding gene was aligned separately, and poorly aligned or ambiguous regions were removed to minimize alignment uncertainty. The refined gene alignments were subsequently concatenated into a combined chloroplast PCG matrix. The best-fit nucleotide substitution model was selected using ModelFinder (Kalyaanamoorthy et al., 2017), and a maximum-likelihood phylogenetic tree was reconstructed using IQ-TREE2 (v2.1.4) (Minh et al., 2020). Branch support was assessed using bootstrap analysis to evaluate the reliability of the inferred nodes. The final tree was visualized and used to determine the evolutionary placement of *S. plebeia* within Lamiaceae. Based on the resulting topology, species with relatively short evolutionary distances to *S. plebeia* were selected for comparative cpgenome analyses, including IR boundary comparison, mVISTA-based sequence similarity analysis, and MUMmer-based whole-plastome collinearity analysis.

### RNA-editing site prediction and codon usage optimization analysis

RNA-editing events, predominantly cytidine-to-uridine (C-to-U) conversions, were systematically predicted to investigate post-transcriptional modifications in the *S. plebeia* mitogenome. Using the PREPACT3 online platform (http://www.prepact.de/prepact-main.php; accessed 6 October 2024) (Lenz and Knoop, 2013), protein sequences from 25 phylogenetically diverse plant species were employed as references, with a BLASTX cutoff of 1e-3 to ensure specificity. Codon preference patterns were analyzed to assess translational optimization in *S. plebeia*. PCGs were extracted from the mitogenome using TBtools (v2.010), and relative synonymous codon usage (RSCU) values were calculated with CodonW (v1.4.4) (Wang et al., 2021). The results of RNA editing prediction and Codon usage analysis were visualized using the ggplot2 package (v3.5.2) of R language (Ito and Murphy, 2013).

### Phylogenetic, collinear and Ka/Ks analysis of *S. plebeia* mitogenome

To resolve the evolutionary position of *S. plebeia* within Lamiaceae, mitogenomes from 24 species across five angiosperm orders (Lamiales, Sapindales, Solanales, Fabales and Rosales) were analyzed, with *Mandragora caulescens PP971602.1* designated as the outgroup. Mitogenome sequences were retrieved from GenBank (accession numbers listed in **Supplementary Table 1**) and standardized using Phylosuite (v1.2.3) (Zhang et al., 2020). Shared PCGs were extracted and concatenated. Multiple sequence alignment (MSA) was performed with MAFFT (v7.313) (Katoh and Standley, 2013), followed by model selection using PartitionFinder2 to identify optimal evolutionary partitions (Rosa et al., 2019). A maximum-likelihood phylogeny was reconstructed with IQ-TREE2, employing bootstrap with 1, 000 replicates to assess nodal support. The final tree was annotated and visualized using iTOL (v5, https://itol.embl.de) (Letunic and Bork, 2021). To investigate structural conservation, mitogenomes of two Salvia species (*S. rosmarinus* PP992923.1 and *S. miltiorrhiza* NC_023209.1) were compared with *S. plebeia* using Mauve (https://darlinglab.org/mauve/mauve.html) (Darling et al., 2004) and MUMmer4 software (Marçais et al., 2018).

To evaluate the selective pressure acting on mitochondrial genes during evolutanalysis was performed using shared mitochondrial PCGs among *S. plebeia* and related species. Orthologous PCG sequences were extracted and aligned in codon mode to preserve the correct reading frame. Pairwise of Ka, Ks and Ka/Ks ratios were calculated using KaKs_Calculator. The Ka/Ks ratio was used to infer the selection pattern of each mitochondrial PCG, with Ka/Ks < 1 indicating purifying selection, Ka/Ks = 1 indicating neutral evolution, and Ka/Ks > 1 indicating potential positive selection. These analyses were used to assess the evolutionary constraints and potential adaptive divergence of mitochondrial genes in *S. plebeia* relative to closely related taxa.

## Results

### Genetic identification of collected specimens

**Figure 1:**
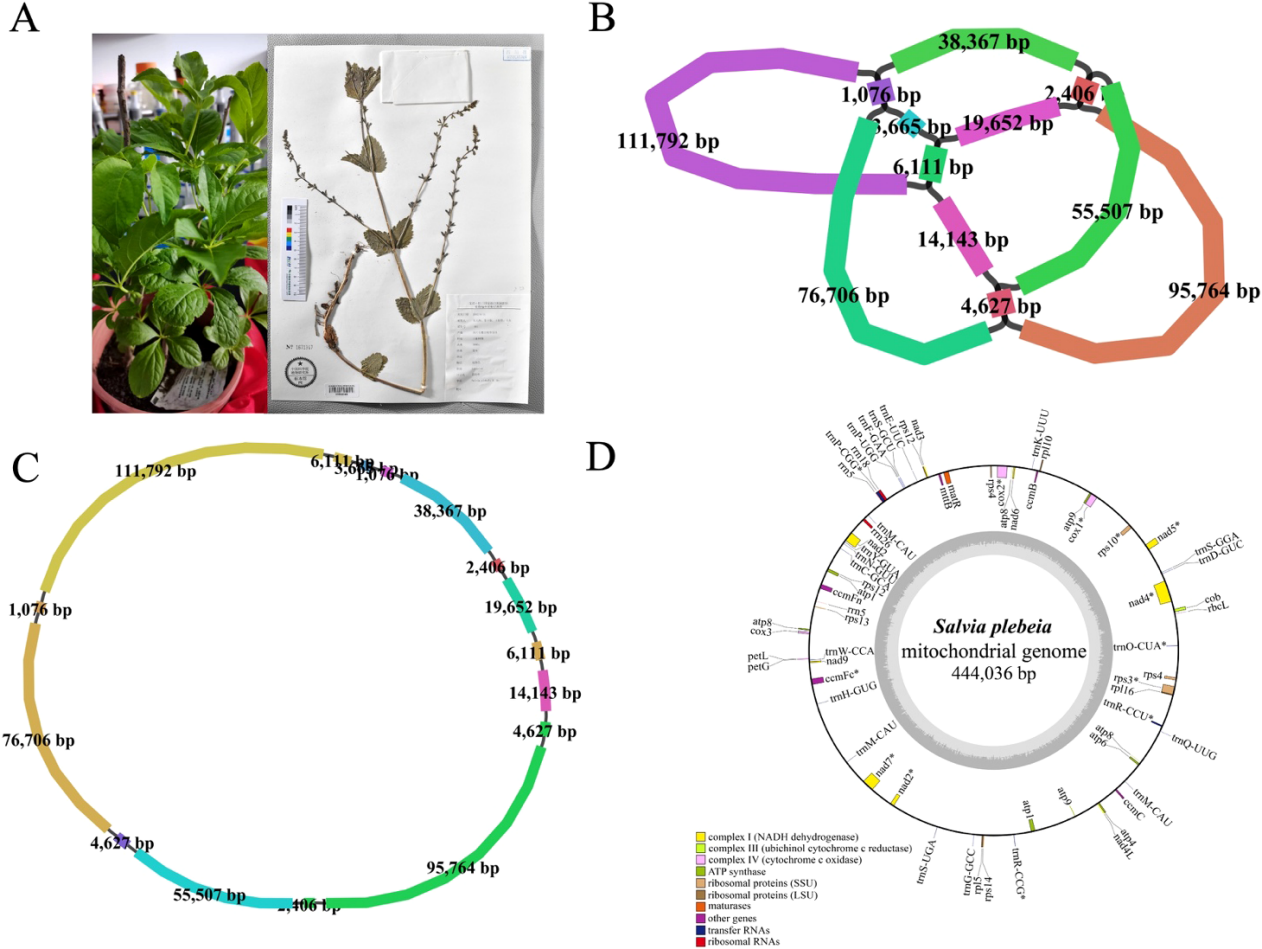
Morphological, genetic, and mitogenome structure overview of *S. plebeia.* **(A)** Morphological characteristics of *S*. *plebeia*. The physical specimen is stored at the Herbarium of the Institute of Botany, Chinese Academy of Sciences. **(B)** Draft of the mitogenome assembly using Bandage. **(C)** Master circular structure of *S. plebeia* mitogenome. **(D)** Circular map of the *S*. *plebeia* mitogenome, showing gene orientations and GC content.

A detailed comparison was conducted with specimens from the National Plant Specimen Resource Center of China Digital Herbarium to ensure accurate identification of the collected samples. The specimen of *S. plebeia* deposited in the Herbarium of the Institute of Botany, Chinese Academy of Sciences, under the specimen number PE01892049, was used as the primary morphological reference for comparison with the collected materials (**Figure 1A**). In addition, the collected specimens were taxonomically examined and confirmed as *S. plebeia* by two botanical experts, Yanhua Yang and Wei Zhang, from the School of Life Sciences, Jiangsu University. For further molecular verification, the *matK* and *rbcL* DNA barcode markers were analyzed against the BOLD database (Gong et al., 2024). The *matK* sequence showed a 100% identity match with *S. plebeia*, providing molecular support for the species-level identification of the collected samples (**Supplementary Table 3**). In contrast, the *rbcL* sequence did not rank *S. plebeia* as the top match, but showed high similarity to species of the genus *Salvia*, with the highest match to *Salvia japonica* var. *japonica* (identity = 99.54%) (**Supplementary Table 4**). This discrepancy is likely due to the relatively conserved nature of the *rbcL* locus, which often contains limited sequence variation among closely related species and therefore may have insufficient discriminatory power at the species level within the genus *Salvia*. By comparison, *matK* generally shows higher sequence variability and provided a more informative barcode signal for species-level identification in this study (Group, C.P.W, 2009; Hollingsworth et al., 2011; Sun et al., 2012; He et al., 2024). To further support the species identity at the organelle-genome level, the assembled cpgenome was compared with the publicly available *S. plebeia* cpgenome from NCBI (NC_050929.1). The two plastomes had the same genome length of 151, 062 bp, and whole-plastome comparison showed 100.00% query coverage, 100.00% reference coverage, and a weighted average sequence identity of 99.9995%. Therefore, the final identification of the collected specimens as *S. plebeia* was supported by morphological comparison, expert taxonomic verification, DNA barcode analysis, and whole-plastome comparison with the published cpgenome.

### Genomic features and structure of *S. plebeia* mitogenome

First, we obtained a sketch of the *S. plebeia* mitogenome assembled from PMAT2 software, containing a total of 12 nodes that were linked to each other to form overlapping regions (**Figure 1B**), and resolved these repetitive regions in turn by selecting the most appropriate pathway supported by the HiFi long read data using minimap2 to obtain accurate conformations (**Figure 1C**). The mitogenome of *S. plebeia* is a circular DNA molecule spanning 444, 036 bp (**Figure 1D**), encoding a total of 58 functional genes essential for mitochondrial energy production, translation, and RNA processing. The genome includes 25 core PCGs involved in oxidative phosphorylation, such as nine subunits of NADH dehydrogenase (*nad1*, *nad2*, *nad3*, *nad4*, *nad4L*, *nad5*, *nad6*, *nad7*, *nad9*), three cytochrome c oxidase subunits (*cox1, cox2, cox3*), one ubiquinol cytochrome c reductase gene (*cob*), five adenosine triphosphate (ATP) synthase subunits (*atp1*, *atp4*, *atp6*, *atp8*, *atp9*), four cytochrome c biogenesis genes (*ccmB*, *ccmC*, *ccmFC*, *ccmFN*), one succinate dehydrogenase subunits (*sdh4*), one maturase gene (*matR*) and one transport membrane protein (*mttB*). The translation machinery is supported by 20 tRNA genes covering all standard amino acids, three ribosomal RNAs (*rrn5*, *rrn18*, *rrn26*), and 13 ribosomal protein genes (10 small subunits: *rps1*, *rps3*, *rps4*, *rps7*, *rps8*, *rps10*, *rps11*, *rps12*, *rps13*, *rps14*; three large subunits: *rpl5*, *rpl10*, *rpl16*) (**Table 1**).

**Table 1:** Encoding genes of *S. plebeia* mitogenome.

Classification	Functional role	Name of genes
Core genes	ATP synthase	*atp1(x2), atp4, atp6, atp8, atp9*
	NADH dehydrogenase	*nad1, nad2, nad3, nad4, nad4L, nad5, nad6, nad7, nad9*
	ubiquinol-cytochrome c reductase	*cob*
	Cytochrome c biogenesis	*ccmB, ccmC, ccmFc, ccmFn*
	Cytochrome c oxidase	*cox1, cox2, cox3*
	Maturases	*matR*
	Transport membrane protein	*mttB*
Variable genes	Large subunit of ribosome	*rpl5, rpl10, rpl16*
	Small subunit of ribosome	*rps3, rps4, rps10, rps12, rps13, rps14*
	Succinate dehydrogenase	*sdh4*
rRNA genes	Ribosomal RNA	*rrn5, rrn18, rrn26*
tRNA genes	Transfer RNA	*trnC-GCA, trnD-GUC, trnE-UUC, trnF-GAA, trnG-GCC, trnH-GUG, trnK-UUU, trnM-CAU(x2), trnN-GUU*
		*trnP-CGG, trnP-UGG, trnQ-UUG, trnR-CCG, trnR-CCU, trnS-GCU, trnS-GGA, trnS-UGA, trnW-CCA, trnY-GUA*

Numbers in parentheses indicate gene copy counts.

**Figure 2:**
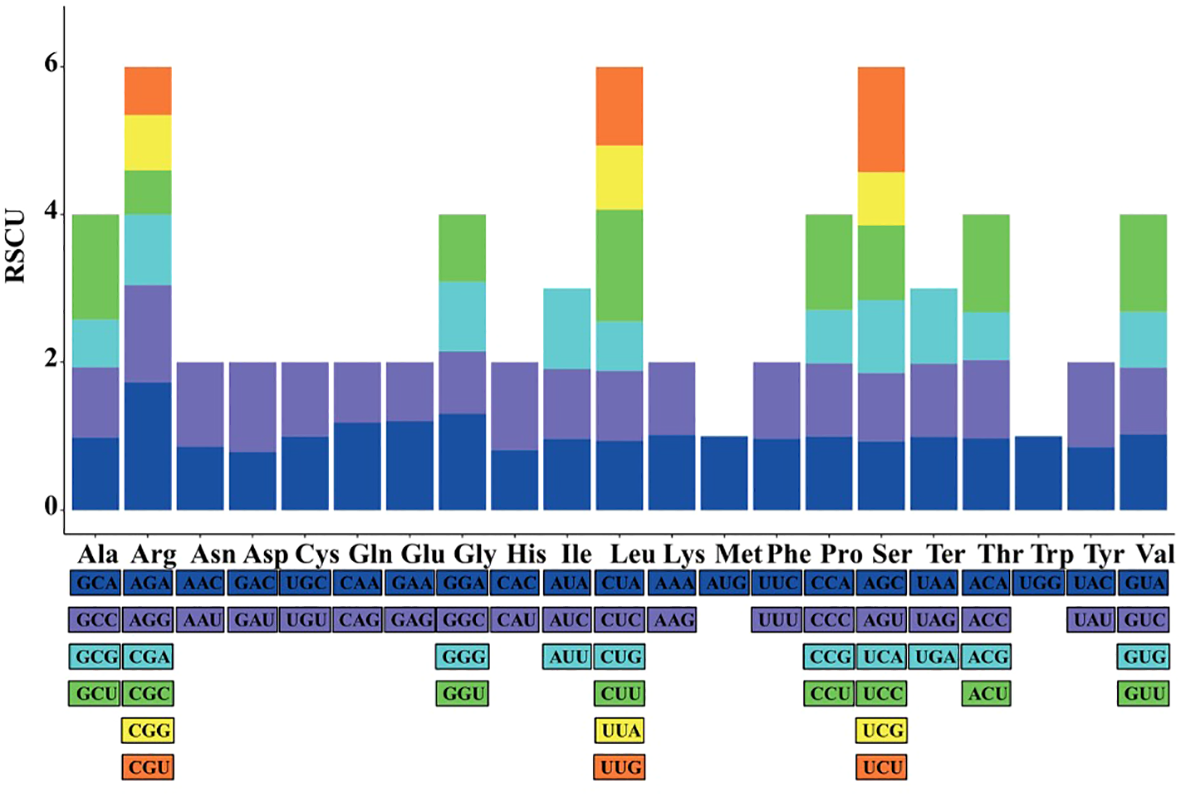
Analysis of codon usage in *S. plebeia* mitogenome.

The mitogenome of *S. plebeia* exhibits distinct codon usage patterns shaped by evolutionary pressures, as revealed through RSCU analysis. Among the 61 codons encoding 20 amino acids (**Figure 2**), pronounced preferences were observed for GCU (Alanine, Ala, RSCU = 1.42), CUU (Leucine, Leu, RSCU = 1.52), UCU (Serine, Ser, RSCU = 1.43), GUU (Valine, Val, RSCU = 1.32), and AGA (Arginine, Arg, RSCU = 1.73), indicating strong translational optimization. Conversely, codons such as GCG (Ala, RSCU = 0.65), CGG (Arg, RSCU = 0.75), UUA (Leu, RSCU = 0.87), and ACG (Threonine, Thr, RSCU = 0.65) were markedly underutilized, reflecting mutational bias or selective avoidance. A striking A/U bias dominated the third codon position, for example, AUA for Isoleucine (Ile, RSCU = 0.96); GAU for Aspartic acid (Asp, RSCU = 1.21), consistent with the AT-rich nature of plant mitogenomes. All PCGs used AUG as the initiation codon. The three stop codons showed similar RSCU values, with UGA being only slightly higher than UAA and UAG, indicating no strong stop-codon bias in the mitochondrial PCGs of S. plebeia. Amino acid usage trends highlighted Leu as the most frequent, driven by CUU (RSCU = 1.52), and Ser, dominated by UCU (RSCU = 1.43). Non-redundant codons for Met (AUG) and Trp (UGG) showed no synonymous bias (RSCU = 1.0) (**Supplementary Table 5**). These findings underscore the interplay of natural selection and mutational drift in shaping codon usage.

### Repeat elements analysis and repeated sequence-mediated homologous recombination

**Figure 3:**
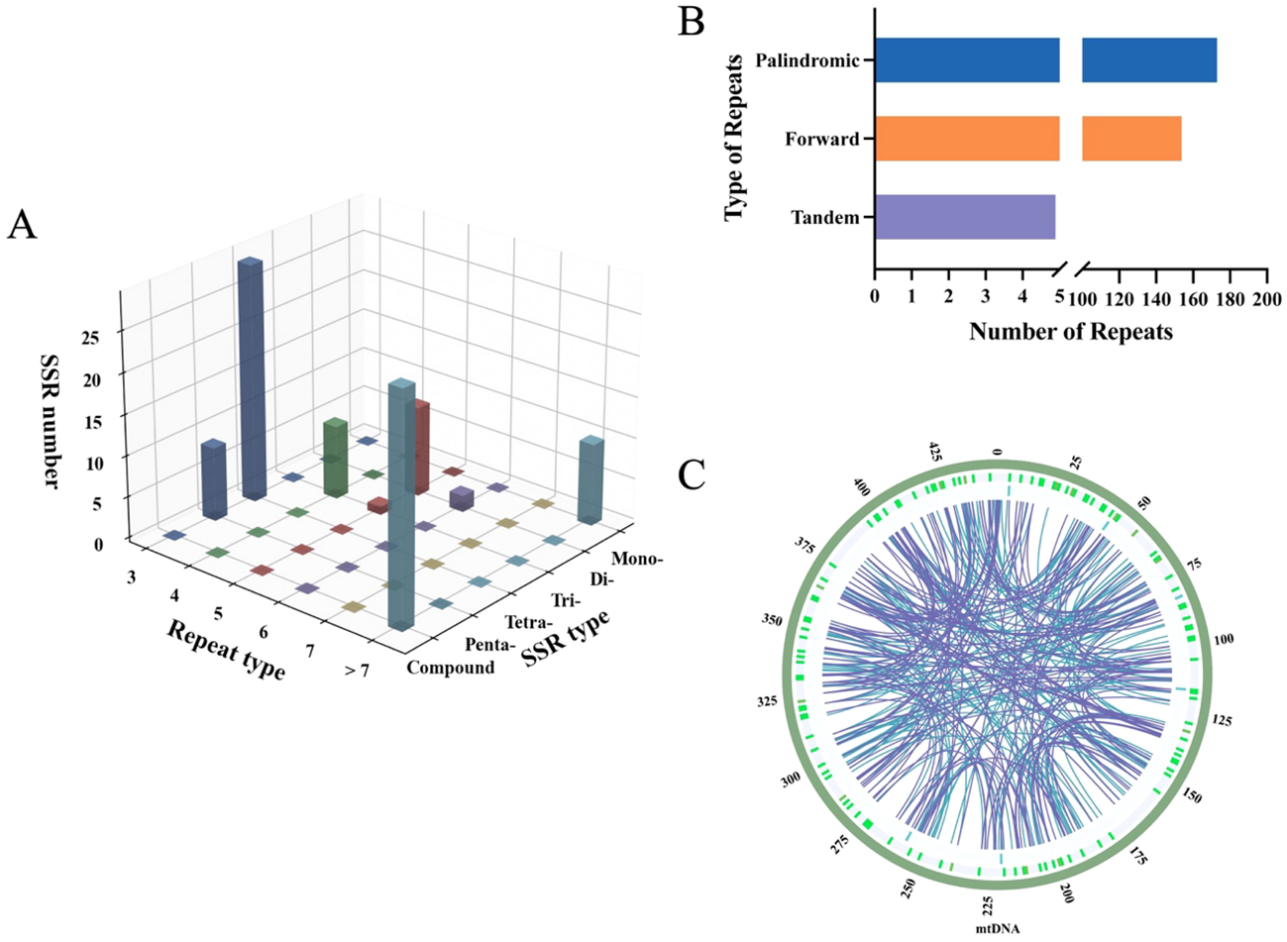
Repeat sequence composition and distribution in *S. plebeia* mitogenome. **(A)** SSRs classified by repeat motif type and repeat number. **(B)** Number of tandem repeats and dispersed repeats, including forward and palindromic repeats, identified in the *S. plebeia* mitogenome. **(C)** Circular visualization of repeat element distribution across the mitogenome. SSRs are shown in the outer track, tandem repeats are shown in the middle track, and dispersed repeats are represented by connecting lines in the inner region. Blue lines indicate forward repeats, and purple lines indicate palindromic repeats.

A comprehensive analysis of SSRs in the *S. plebeia* mitogenome identified 135 repeats ranging in length from 10 to 1679 bp (**Supplementary Table 6**). These SSRs were classified by nucleotide type, with tetranucleotide repeats (p4) being the most frequent (29 occurrences), followed by compound repeats (p2) (28 occurrences) and dinucleotide repeats (p2) (9 occurrences). Trinucleotide (p3), mononucleotide and pentanucleotide repeats appeared less frequently (10, 10 and 9 occurrences, respectively), while hexanucleotide repeats (p6) were not found in *S. plebeia* (**Figure 3A**). The predominance of tetranucleotide repeats suggests their potential role in maintaining genome stability and evolutionary adaptability.

Dispersed repeats constituted the majority of repetitive elements, with 331 repeats identified, including 156 forward repeats and 175 palindromic repeats, spanning 30 bp to 6, 111 bp (**Figure 3B**; **Supplementary Table 7**). In contrast, tandem repeats were relatively scarce, reflecting their distinct genomic distribution and functional roles (**Supplementary Table 8**). The spatial arrangement of all repeat elements, SSRs, tandem repeats, and dispersed repeats—was graphically represented across the mitogenome (**Figure 3C**), highlighting their localization patterns. This mapping provides critical insights into the organizational framework and potential functional implications of repetitive sequences in the *S. plebeia* mitogenome.

**Figure 4:**
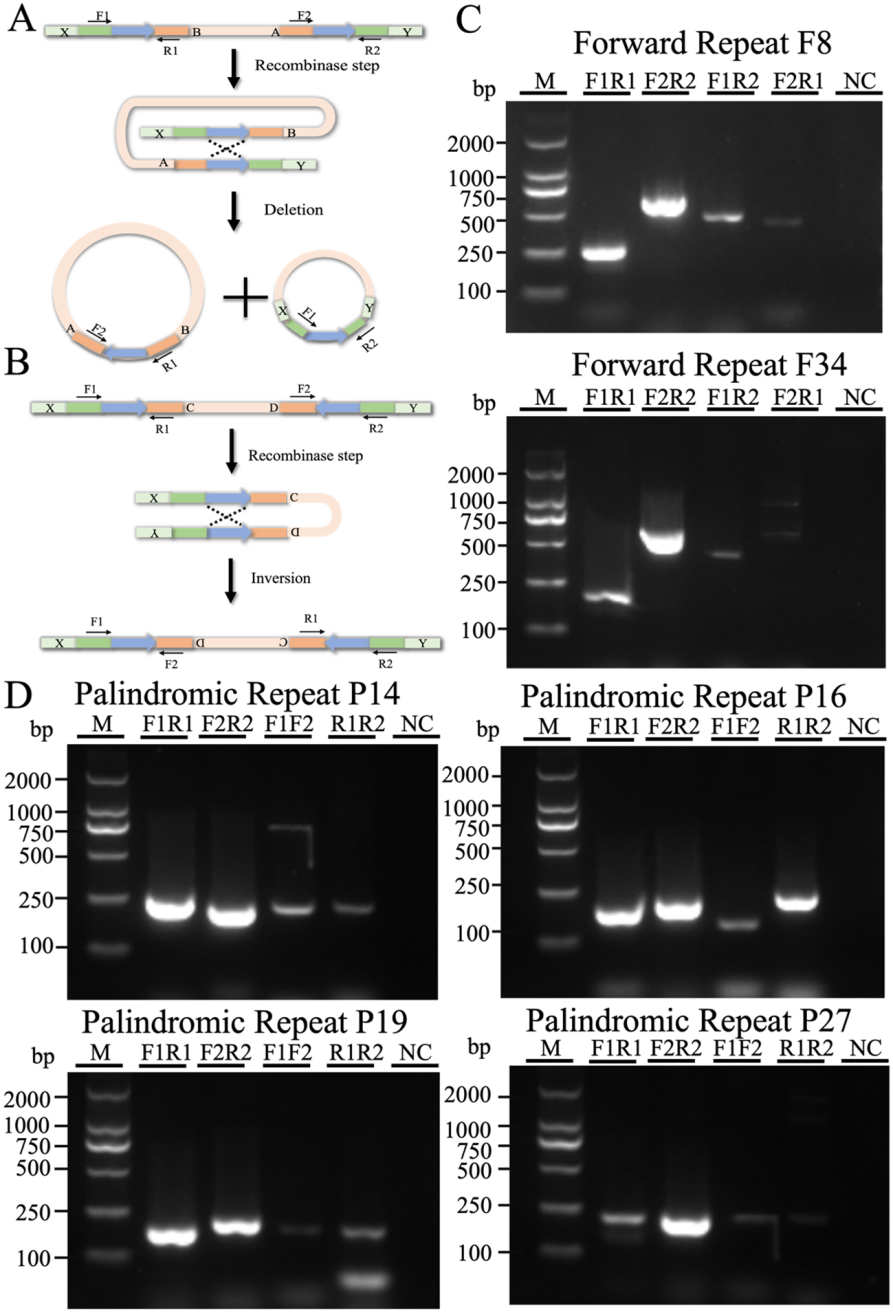
Structural diversity of mitochondrial DNA driven by repeat-mediated recombination events. **(A, B)** Diagrammatic illustration of the recombination mechanism mediated by palindromic and forward repeats, highlighting the generation of diverse mitochondrial DNA conformations. **(C, D)** PCR results showcase the multiple configurations of mitochondrial DNA, with each panel displaying six lanes: (1) molecular marker, (2) and (3) two major conformations, (4) and (5) two minor conformations and (6) negative control corresponding to recombination within six repeat sequences (F8, F34, P14, P16, P19 and P27). The PCR bands clearly demonstrate the structural differences among these conformations.

To investigate potential recombination events mediated by forward and palindromic repeats in the mitogenome, HiFi reads were aligned to the predicted reference and recombinant conformations, which identified several read-supported candidate repeat pairs, including two forward repeats (F8 and F34) and four palindromic repeats (P14, P16, P19, and P27). Schematic representations detail the recombination mechanisms (**Figures 4A, B**). For forward repeat-mediated recombination (F8 and F34), conventional homologous recombination occurs through sequence alignment in same-strand orientation, where the red DNA segment moves between tandemly arranged repeats. Conversely, palindromic repeats (P14, P16, P19 and P27) facilitate structural inversion via complementary strand pairing through hairpin formation, with the red segment undergoing intramolecular rearrangement. The dark red and green boxes represent recombinase recognition sequences, while black arrows indicate crossover regions, collectively forming recombination sites. Genes A, B, C, D, X and Y are shown in their respective genomic positions. To investigate these mechanisms experimentally, distinct PCR strategies were employed. For forward repeats, primer pairs F1&R2 and F2&R1 amplified potential homologous recombination products, while native configurations were verified using conventional flanking primers F1&R1 and F2&R2. Palindromic repeat analysis utilized unconventional primer combinations F1&F2 and R1&R2 to detect inverted rearrangements, with control amplifications using F1&R1 and F2&R2 primer pairs (**Table 2**). Negative controls omitted PCR templates. Results from agarose gel electrophoresis revealed dynamic equilibrium between native and recombinant conformations (**Figures 4C, D**). Both parental and recombined Mitochondrial DNA (mtDNA) bands were simultaneously present, demonstrating ongoing recombination events *in vivo*. This co-amplification pattern validates the proposed models where forward repeats mediate sequence exchange through strand alignment, while palindromic repeats induce structural inversion via hairpin-mediated recombination.

**Table 2:** Primer sequences and PCR conditions for verification of homologous recombination mediated by repetitive sequences.

Repeats	Primers	Sequence 5’-3’	Product length (bp)	Annealing temperature (°C)
F8	F8-F1	TCTCTTCTCGGCTTACCCCT	263	F1&R1:57
	F8-R1	GAAGAAGCGGGAATGTGGGT		F2&R2:57
	F8-F2	TCCTTCTAACCCGCCCGAG	619	F1&R2:57
	F8-R2	TGCGCCTGTGCTTTTTGC		F2&R1:57
F34	F34-F1	TCTAACACGGATTGACAAGC	205	F1&R1:57
	F34-R1	AGATACCCCGCCGCAAC		F2&R2:57
	F34-F2	AGGGATTCCACTGCTCCCT	614	F1&R2:57
	F34-R2	ATGACCATGACCCGATTAGT		F2&R1:57
P14	P14-F1	ACCGGCGCATCTTTAGTTTT	188	F1&R1:57
	P14-R1	CATCAGGGACGAAGAGTTGC		F2&R2:57
	P14-F2	GACGGGTTTTCCAGTAGGCA	216	F1&F2:60
	P1-R2	GACGCTCTCGGAGCTACCA		R1&R2:60
P16	P16-F1	ACGGCTTGTCAAAAGAACCA	258	F1&R1:57
	P16-R1	AGTTTTGGGCCTTCAACGT		F2&R2:57
	P16-F2	AGGCTGAGAGAGGAGAGACT	230	F1&F2:60
	P16-R2	CTTTAGCAGCTCACCCGTAA		R1&R2:60
P19	P19-F1	CAACCATCAGGGAGAGTTTC	176	F1&R1:57
	P19-R1	CAAGGAGCTTCTCTTACCGA		F2&R2:57
	P19-F2	TGGGGCAGTACCAAAATATG	222	F1&F2:57
	P19-R2	GCTTTGAAACCTTTAGCTCG		R1&R2:57
P27	P27-F1	GCTCTCCCAATGAAGGAAG	226	F1&R1:57
	P27-R1	AGAATGAATGCAAGGGGGGA		F2&R2:57
	P27-F2	GCGTCAACTCCCACTTCTTT	234	F1&F2:57
	P27-R2	AGTGGAAACATGAGTTGGCC		R1&R2:57

The designation F1&R1:57 indicates that the annealing temperature for the primer pair F1&R1 was set at 57 degrees Celsius.

### Mitochondrial-plastid DNA transfer and cpgenome characterization

**Figure 5:**
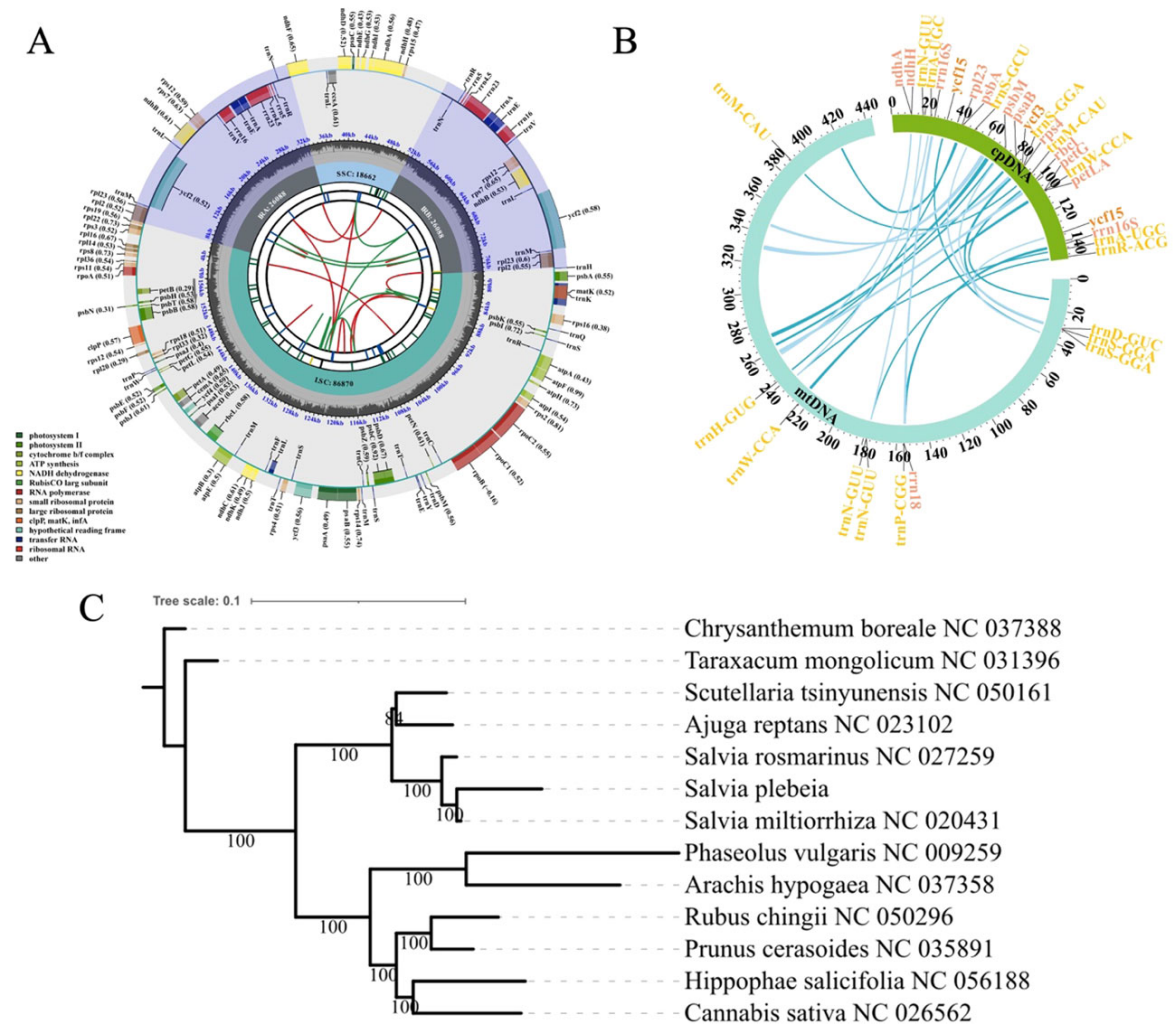
Cpgenome features and phylogenetic analysis of *S. plebeia*. **(A)** Circular map of the *S. plebeia* chloroplast genome. Genes are color-coded according to functional categories, and the large single-copy region, small single-copy region, and two IR regions are indicated. **(B)** Circos visualization of homologous fragments between the cpgenome and mitogenome, showing putative plastid-derived mitochondrial sequences. **(C)** Maximum-likelihood phylogenetic tree inferred from chloroplast PCGs, showing the phylogenetic placement of *S. plebeia* within related taxa. Numbers at nodes indicate bootstrap support values.

The cpgenome of *S. plebeia* was assembled and annotated, yielding a circular cpgenome of 151, 062 bp in length (**Figure 5A**). This fully resolved genome features a canonical quadripartite structure comprising an 86, 870 bp LSC region, an 18, 662 bp SSC region, and two IR regions each spanning 26, 088 bp. Functional annotation identified core chloroplast genes critical for photosynthetic machinery and genome maintenance, including *ycf2*, *ycf3*, *ycf4*, *rpoA*, *rpoB*, *rpoC1*, *rpoC2*, *rbcL* and *ndh* genes (*ndhA-K*). These conserved loci, along with other structural and regulatory elements, collectively define the plastome’s functional architecture. The complete genome sequence and structural characterization provided a foundational genomic framework for subsequent comparative analyses of chloroplast evolution and organization in related taxa. Further investigation into intracellular sequence transfer revealed the presence of MTPTs, which are DNA fragments transferred from the cpgenome into the mitogenome (**Figure 5B**). A total of 25 MTPTs, spanning 11, 540 bp, were found to be shared between the cpgenomes and mitogenomes, representing 2.60% of the mitogenome and 10.19% of the cpgenome of *S. plebeia* (**Supplementary Table 9**). The MTPT lengths ranged from 61 to 2, 138 bp, labeled MTPT1 through MTPT25, revealing functional genes in 15 segments. Some MTPTs contained partial rRNA genes (e.g., *rrn16S* in cpgenome and *rrn18* in mitogenome), specifically in MTPT19 and MTPT20. Additionally, several MTPTs contained tRNA genes, including *trnA-UGC* (MTPT1, MTPT2), *trnM-CAU* (MTPT3), *trnH-GUG* (MTPT4), *trnN-GUU* (MTPT5), *trnR-ACG* (MTPT6), *trnS-GCU* (MTPT7), *trnS-GGA* (MTPT8), *trnD-GUC* (MTPT9), and *trnP-CGG* (MTPT19 and MTPT20). Furthermore, plastid-derived PCGs, predominantly fragmented, included: *rpl23* (MTPT4), *psbM* (MTPT9), *rbcl* (MTPT11), *rps4* (MTPT14), *ndhA* and *ndhH* (MTPT15), *psbA* (MTPT17), *psaB* (MTPT18), *ycf3* (MTPT22), and *petL* and *petG* (MTPT23). No functional genes were annotated in MTPT10, MTPT12, MTPT13, MTPT16, MTPT21, MTPT24 and MTPT25. These results confirm that tRNAs were completely transferred and are potentially functional, while plastid PCGs exist as partial fragments unlikely to retain functionality. Collectively, these MTPTs demonstrate dynamic inter-organellar gene transfer, driving genome evolution in *S. plebeia*.

### Comparative and evolutionary analysis of cpgenomes

Comparative analysis of cpgenome boundaries in *S. plebeia* and related Lamiaceae species revealed both structural conservation and localized boundary variation. The expansion and contraction of inverted repeat regions contributed to size variation at the junctions of the LSC, SSC, and IR regions. The IRB/SSC boundary (JSB) showed relatively high plasticity, with partial extension of the IRB region toward the *ndhF* gene by approximately 1–42 bp, while the SSC region varied from 17, 498 to 17, 922 bp. In contrast, the IR regions were relatively conserved, with IRA and IRB lengths ranging from 25, 521 to 25, 687 bp, and the LSC region ranged from 82, 454 to 85, 148 bp. The JLB and IRA/LSC (JLA) boundaries maintained conserved near-terminal extensions of approximately 102–113 bp (**Supplementary Figure 1A**). These results suggest that the *S. plebeia* cpgenome retains the typical quadripartite structure of angiosperm plastomes, while minor IR boundary shifts may represent lineage-specific evolutionary changes within Lamiaceae.

The mVISTA analysis, using *S. plebeia* (NC_050929.1) as the reference genome, visualized sequence conservation and divergence across the complete chloroplast genomes of four *Salvia* species, including *S. plebeia*, *S. rosmarinus*, *S. miltiorrhiza*, and *S. yangii* (**Supplementary Figure 1B**). The results showed a high level of sequence conservation across the compared plastomes, especially in functionally important coding regions. Photosynthesis-associated genes, including photosystem genes such as *psbA* and *psbD*, the Rubisco large-subunit gene *rbcL*, electron transport-related genes such as *petB*, and ATP synthase genes including *atpA*, *atpB*, *atpE*, and *atpF*, displayed strong conservation. Similarly, ribosomal RNA genes, including *rrn16*, *rrn23*, and *rrn4.5*, also showed exceptionally high sequence identity. These highly conserved regions indicate strong purifying selection acting on genes involved in photosynthesis and chloroplast protein synthesis. In contrast, greater sequence divergence was mainly observed in non-coding regions, including intergenic spacers and introns, particularly in single-copy regions. These variable regions may reflect relaxed selective constraints and could provide useful molecular markers for species identification, population genetic analysis, and plastome evolutionary studies in *Salvia*.

Whole-plastome alignment using MUMmer4 further confirmed the high structural conservation of the *S. plebeia* chloroplast genome. Dot-plot comparisons between the assembled *S. plebeia* cpgenome and three representative *Salvia* chloroplast genomes showed dense and nearly continuous diagonal alignment signals across almost the entire genome length (**Supplementary Figure 2**). This pattern indicates strong collinearity, high synteny, and limited large-scale structural rearrangement among the compared plastomes. In particular, the high consistency between the newly assembled *S. plebeia* cpgenome and the previously reported *S. plebeia* cpgenome confirmed the reliability of the present assembly. Comparisons with *S. rosmarinus*, *S. miltiorrhiza*, and *S. yangii* also showed that cpgenome architecture is highly conserved within the genus *Salvia*, suggesting evolutionary stability of plastome structure in this lineage.

Phylogenetic analysis based on cpgenome sequences further clarified the evolutionary position of *S. plebeia* within Lamiaceae (**Figure 5C**). The newly assembled *S. plebeia* cpgenome clustered closely with the published *S. plebeia* (NC_050929.1) cpgenome, supporting the taxonomic identity and assembly accuracy of the present plastome. Within the phylogenetic framework, *S. plebeia* was placed within the *Salvia* lineage and showed a close evolutionary relationship with other *Salvia* species, including *S. rosmarinus*, *S. miltiorrhiza*, and *S. yangii*. This topology was consistent with the high sequence similarity and strong genome collinearity observed in the comparative analyses. Compared with more distant Lamiaceae species such as *Platostoma chinense* and *Vitex trifolia*, the *Salvia* plastomes showed greater structural and sequence conservation, indicating that cpgenome evolution in *S. plebeia* is characterized by overall architectural stability, strong conservation of core functional genes, and limited divergence mainly concentrated in non-coding regions and IR boundary regions. Collectively, these results demonstrate that the cpgenome of *S. plebeia* is evolutionarily conserved within *Salvia*, while localized sequence divergence and minor IR boundary variation provide evidence of lineage-specific plastome evolution.

### Variation and RNA-editing site analysis in mitochondrial PCGs

The mitogenome of *S. plebeia* exhibited both evolutionary conservation and dynamic adaptability, as revealed through comparative sequence analysis and RNA-editing studies. Nucleotide and amino acid alignments of seven PCGs (*atp8*, *cob*, *nad1*, *nad2*, *nad4*, *nad5 and cox1*) across three Lamiaceae species (*S. plebeia*, *S. rosmarinus* and *P. chinense*) demonstrated high sequence similarity in core functional domains, particularly in oxidative phosphorylation genes like *atp8, nad3* and *nad4* (**Supplementary Figure 3**). Their high sequence conservation across species reflected strong purifying selection to preserve enzymatic efficiency and structural integrity. However, localized divergences were observed in *cob, cox nad1* and *nad5*, marked by mismatches and indels (red boxes). These variations may enhance mitochondrial resilience to stressors like temperature fluctuations or oxidative damage, enabling *S. plebeia* to thrive in diverse habitats. The mismatches and indels in *nad1* and *cox1* further highlighted lineage-specific adaptations. *Nad1* participates in NADH dehydrogenase activity, and its structural variations could modulate redox balance, while *cox1* alterations might influence oxygen utilization efficiency. Such modifications may reflect trade-offs between metabolic performance and energy conservation in response to ecological niches.

**Figure 6:**
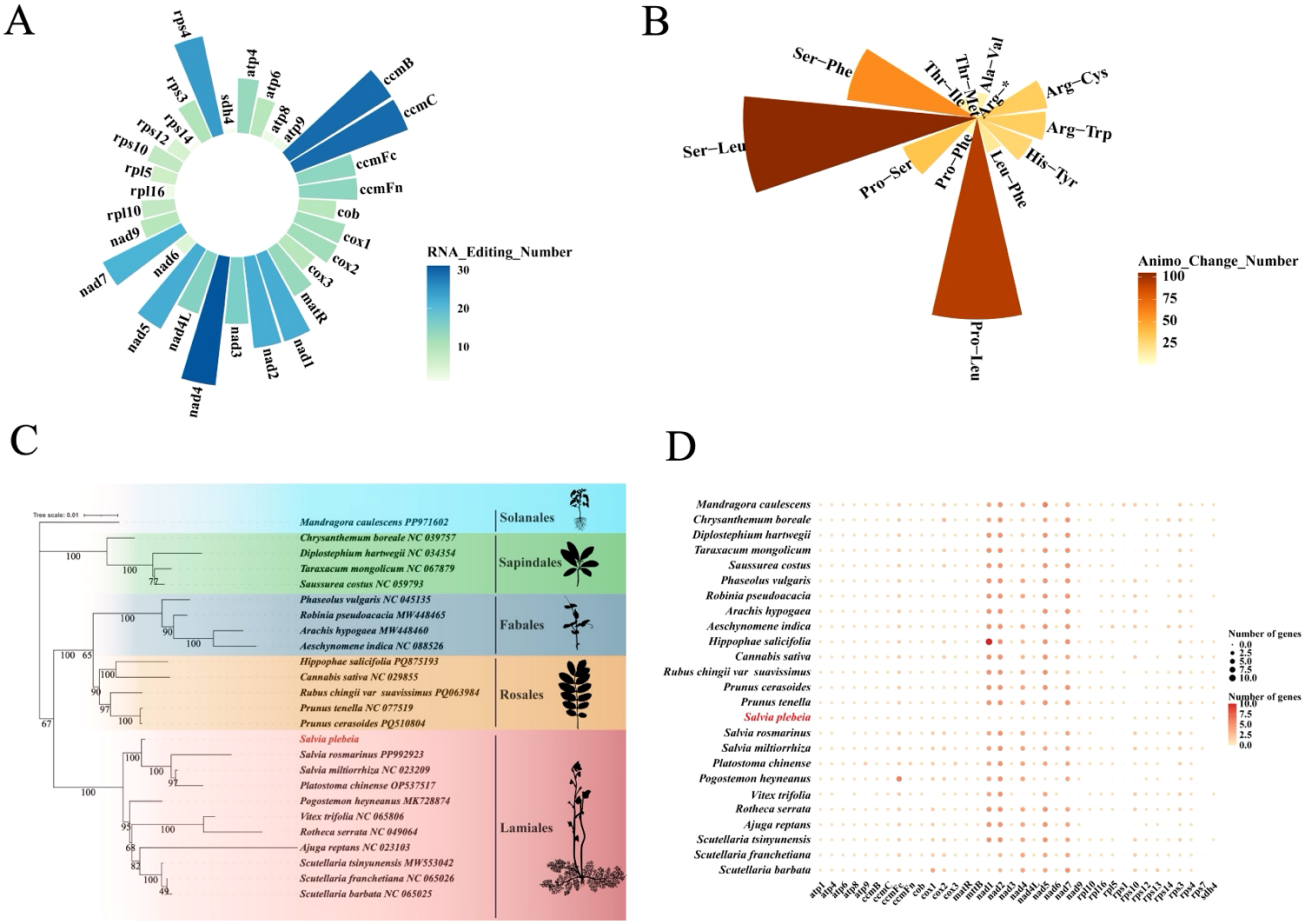
Mitochondrial phylogenetic relationships and comparative RNA-editing profiles of *S. plebeia* and related species. **(A)** Distribution of predicted RNA-editing sites across mitochondrial PCGs in *S. plebeia*, with the number of RNA-editing sites indicated for each gene. **(B)** Analysis of amino acid substitutions resulting from RNA-editing events, detailing the types and frequencies of observed changes. **(C)** Maximum-likelihood phylogenetic tree reconstructed from mitochondrial PCGs of *S. plebeia* and representative plant species. *S. plebeia* is highlighted in red, and major taxonomic groups are indicated by different background colors. Numbers at nodes represent bootstrap support values. **(D)** Bubble plot showing the distribution of predicted RNA-editing sites across mitochondrial PCGs in *S. plebeia* and related species. Bubble size and color intensity indicate the number of predicted RNA-editing sites for each gene.

RNA editing, a crucial post-transcriptional process in plant mitochondria, involves site-specific nucleotide modifications that influence protein functionality. This study identified 587 RNA-editing events across mitochondrial PCGs, predominantly characterized by C-to-U transitions (**Figure 6A**; **Supplementary Table 10**). Among the 33 analyzed PCGs, *nad4* exhibited the highest number of editing sites (42 instances), followed by *ccmFn* (35 instances) and *ccmB* (34 instances), while genes such as *rps14*, *atp8* and *rps10* showed fewer than five edits each. Notably, these edits frequently led to amino acid substitutions, with Ser-to-Leu (105 instances) and Pro-to-Leu (94 instances) being the most prevalent changes, often converting hydrophobic residues to hydrophilic ones (**Figure 6B**; **Supplementary Table 11**). Such alterations may critically impact protein structure, enzymatic activity, or stability, potentially facilitating mitochondrial adaptation under varying physiological conditions. The high frequency of functional amino acid changes underscores the regulatory role of RNA editing in fine-tuning mitochondrial gene expression and protein properties.

### Evolutionary relationships among closely related species

The mitochondrial PCG-based phylogeny in **Figure 6C** resolves evolutionary relationships among 24 species spanning five plant orders (Lamiales, Sapindales, Solanales, Fabales, Rosales), rooted with *Mandragora caulescens*, a species from the order Solanales, designated as the outgroup. Branching patterns align with taxonomic classification, supported by high bootstrap values (close to 100), reflecting robust statistical confidence for the evolutionary relationships. Within Lamiales, *S. plebeia* clusters tightly with congeneric Lamiaceae species (*S. rosmarinus*, *S. miltiorrhiza* and *P. chinense*), corroborating their shared evolutionary trajectory and recent diversification. This phylogenetic coherence underscores both the genetic proximity of Lamiaceae taxa and the distinctive genomic features of *S. plebeia* relative to its Salvia relatives.

Complementing this, **Figure 6D** illustrates mitochondrial gene conservation across taxa using a comparative dot plot. *S. plebeia* and its Lamiaceae relatives exhibit conserved abundance of *nad* family genes (*nad1*, *nad2*, *nad4*), critical for electron transport chain function, suggesting evolutionary retention of core respiratory machinery. Collectively, the conserved mitochondrial gene architecture within Lamiaceae and divergent patterns across orders provide dual insights: (1) shared physiological or metabolic traits may drive gene conservation, and (2) lineage-specific gene losses illuminate adaptive evolution. These integrative analyses including combining phylogenomics, bootstrap-supported branching and gene content visualization establish a framework for exploring mitogenome plasticity, evolutionary constraints, and the medicinal potential of species like *S. plebeia*.

### Synteny analysis of plant mitogenomes

**Figure 7:**
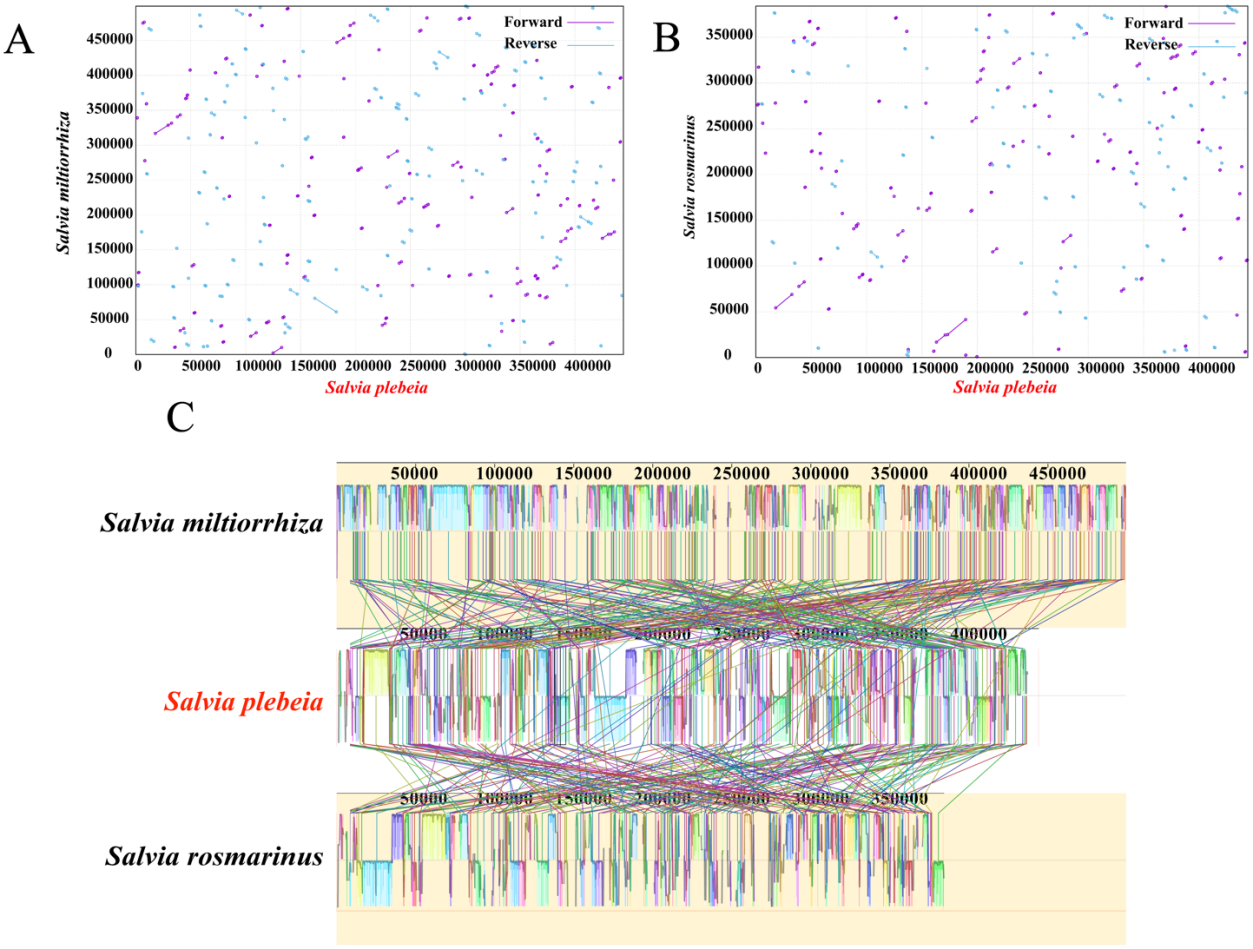
Collinearity analysis of *S. plebeia* and related species. **(A)** displays dot plots comparing the genomic sequence of *S. plebeia* against *S. miltiorrhiza*. **(B)** presents dot plots comparing the genomic sequence of *S. plebeia* against *S. rosmarinus*. **(C)** illustrates a synteny plot comparing the mitogenome of *S. plebeia* with *S. miltiorrhiza* and *S. rosmarinus*, highlighting conserved regions and structural similarities.

Plant mitogenomes exhibit a paradoxical conservation pattern: while functional gene content (number, type and sequence) remains highly conserved across species, gene arrangement and synteny vary dramatically due to frequent genomic rearrangements. Comparative analyses of closely related species reveal large syntenic blocks rich in homologous sequences, which serve as critical markers for inferring evolutionary relationships, genetic kinship, and refining genome annotations. For instance, *S. miltiorrhiza* and *S. rosmarinus with S. plebeia* display strong dot plot alignment (**Figures 7A, B**), reflecting high sequence similarity and conserved synteny consistent with their evolutionary proximity. Additionally, mauve collinearity analysis further resolves this complexity: while conserved homologous blocks exist among *S. plebeia*, *S. rosmarinus*, and *S. miltiorrhiza* (**Figure 7C**), their scrambled order highlights extensive genomic rearrangements in *S. plebeia*, resulting in a structurally variable mitogenome with low organizational conservation.

### Evolutionary dynamics and structural variation of the *S. plebeia* mitogenome

**Figure 8:**
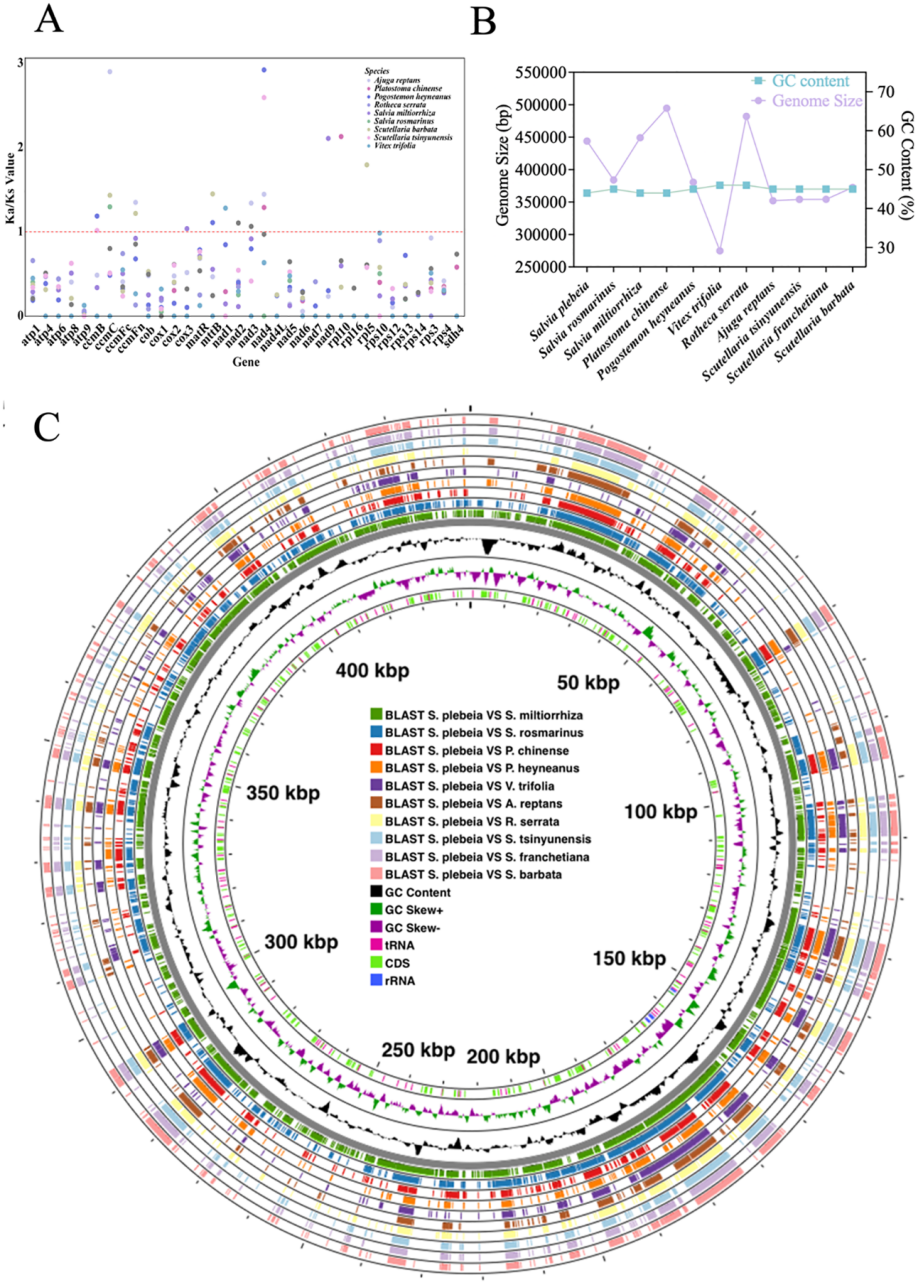
Comparative genomic analysis of *S. plebeia* and related species. **(A)** displays dot plots comparing the Ka/Ks values of PCGs in mitogenome of *S. plebeia* versus ten Lamiales species. **(B)** presents line chats comparing the genomic size and GC content of o *S. plebeia* and other ten mitogenomes. **(C)** shows the BLAST alignment of *S. plebeia* against other ten mitogenomes, with a detailed comparison at gene level.

The mitogenome of *S. plebeia* was analyzed for evolutionary dynamics and structural features through Ka/Ks ratio calculations, GC content profiling, and comparative genomics. Dot plots compare the ratios of Ka to Ks substitution rates for mitochondrial genes between *S. plebeia* and ten Lamiales species. The analysis of Ka/Ks ratios revealed predominant purifying selection, with most genes exhibiting values < 1, like *atp1* in *S. miltiorrhiza* (0.288) and *nad1* in *A. reptans* (0.145), indicative of strong evolutionary constraints conserving amino acid sequences. Notably, Ka/Ks = 0 was observed in genes such as *nad4L* in *R. serrata* and *V. trifolia*, likely reflecting undetectable synonymous substitutions or complete absence of nonsynonymous changes, suggesting extreme functional conservation. Despite this conservative trend, signatures of positive selection (Ka/Ks > 1) were identified in specific lineages: *ccmB* in *S. barbata* (1.450) and *nad4* in *P. heyneanus* (2.922) and *S. tsinyunensis* (2.594), potentially linked to adaptive evolution in cytochrome c biogenesis and energy metabolism, respectively. Heterogeneous selection pressures were evident in genes such as *rps3*, displaying near-neutral evolution in *A. reptans* (0.927) but purifying selection in *V. trifolia* (0.510), highlighting lineage-specific evolutionary dynamics. These results collectively demonstrate that while purifying selection dominates mitochondrial gene evolution, key genes such as *ccmB* and *nad4* undergo positive selection in specific taxa, driving functional innovation or environmental adaptation. The observed variability in Ka/Ks ratios among closely related species underscores the dynamic interplay of evolutionary forces shaping plant mitogenomes. (**Figure 8A**). Line charts contrast the mitogenome size and guanine‐cytosine (GC) composition of *S. plebeia* with ten related species. *S. plebeia* has a 444 kb genome, intermediate within Lamiaceae (250–550 kb range), with its size variation attributed to lineage-specific repeat expansions or gene losses. The GC content of *S. plebeia* (44%) aligns with most Lamiales species (**Figure 8B**). These metrics underscore structural plasticity despite conserved functional gene content. The online tool Proksee (https://proksee.ca) was used to perform comparative analyses of mitogenome structures across species. The first to tenth tracks, moving from the outside to the inside of the circles, indicate the results of BLAST alignment of the DNA sequences of *S.plebeia* with ten Lamiales species. BLAST alignments reveal high sequence homology between *S. plebeia* and congeneric species (*S. miltiorrhiza* and *S. rosmarinus*) (**Figure 8C**).

## Discussion

Plant organelle genomes are closely associated with plant evolution, phylogenetic diversification, and genome structural dynamics (Mower, 2020; Shi et al., 2023; Wang et al., 2024; Yu et al., 2026). Cpgenomes usually retain relatively conserved structures and provide useful information for species identification and phylogenetic inference, whereas plant mitogenomes are more variable in size, structure, and organization, often reflecting extensive rearrangements, sequence transfer, and repeat-associated recombination (Gray, 2012; Moller et al., 2021). Therefore, integrated analysis of mitochondrial and chloroplast genomes can provide important insights into organelle genome evolution and lineage-specific genomic features. In the present study, both mitochondrial and chloroplast genomes of *S. plebeia* were assembled and characterized, providing an organelle-level genomic framework for this medicinally important species. The *S. plebeia* mitogenome was represented as a circular-mapping molecule of 444, 036 bp and contained conserved mitochondrial genes, including core protein-coding genes, ribosomal protein genes, tRNA genes, and rRNA genes. The co-assembled cpgenome was 151, 062 bp in length and exhibited a typical quadripartite structure, consisting of a large single-copy region of 86, 870 bp, a small single-copy region of 18, 862 bp, and two IR regions of 22, 665 bp each.

Building on this genomic characterization, the abundance of SSRs and dispersed repeats in the *S. plebeia* mitogenome suggests that repetitive sequences are important contributors to mitogenome structural dynamics. Here, tetranucleotide SSRs and dispersed repeats were the dominant repeat types, whereas tandem repeats were relatively rare, indicating that different repeat classes may be subject to distinct evolutionary constraints in the *S. plebeia* mitogenome. Long or highly similar dispersed repeats can provide homologous regions for recombination and may therefore increase the structural flexibility of plant mitogenomes. The repeat-rich architecture observed in *S. plebeia* is consistent with the general feature of plant mitogenomes, in which repetitive sequences often contribute to genome rearrangement, alternative conformations, and structural diversity while conserved mitochondrial gene content is largely retained.

Given the prevalence of these repeats, repeat-mediated homologous recombination is likely to play a key role in shaping mitogenome architecture. In this study, several repeat pairs longer than 100 bp were selected for recombination validation, and six repeat pairs were experimentally supported as recombinationally active. These results indicate that the identified repeats are not only static sequence elements but may also participate in the generation of alternative mitochondrial conformations. Forward and palindromic repeats may mediate recombination through different structural routes, thereby contributing to local rearrangements or alternative genomic configurations. Such recombination may partly explain the structural complexity of the *S. plebeia* mitogenome and provides direct evidence for repeat-associated mitogenome plasticity in this species. Similar repeat-mediated structural variation has been reported in other plant mitogenomes and is considered an important force driving mitogenome evolution (David et al., 2017; Cavassim et al., 2021). However, the present study mainly supports the role of these repeats in structural rearrangement. Whether these recombination events affect mitochondrial gene expression, local gene organization, or protein-coding ORFs remains to be further investigated using ORF prediction, transcriptome analysis, and functional validation.

The codon usage and RNA-editing analyses provide complementary information on mitochondrial PCGs in *S. plebeia*. The pronounced A/U bias at the third codon position and the preferential use of codons such as CUU and GCU are consistent with the generally AT-rich nature of plant mitogenomes. These patterns may reflect the combined effects of mutational bias, codon composition, and mitochondrial translational constraints. In this context, codon usage represents a genomic-level feature of mitochondrial protein-coding sequences, whereas RNA editing acts at the post-transcriptional level to modify mitochondrial transcripts and potentially restore conserved amino acids. The predicted RNA-editing sites, mainly involving C-to-U changes, suggest that post-transcriptional modification may play an important role in shaping the final mitochondrial protein products of *S. plebeia*. Amino acid changes such as Ser-to-Leu and Pro-to-Leu may alter protein hydrophobicity and contribute to the conservation of functional mitochondrial proteins. Editing events in genes related to NADH dehydrogenase and cytochrome c maturation may be particularly relevant to mitochondrial respiratory function. Nevertheless, these RNA-editing sites were predicted computationally, and their actual occurrence and biological effects should be further confirmed using transcriptome-based evidence.

The chloroplast and mitochondrial phylogenetic analyses provided broadly consistent support for the placement of *S. plebeia* within the *Salvia* lineage, but they also showed minor differences in the relationships among closely related species. In the chloroplast protein-coding gene-based tree, *S. plebeia* clustered closely with *S. miltiorrhiza*, whereas the mitochondrial protein-coding gene-based tree placed *S. plebeia* as a close relative of the *S. rosmarinus*–*S. miltiorrhiza* clade. This overall agreement supports the taxonomic placement of the collected material and provides an organelle-level evolutionary framework for *S. plebeia*. The slight topological differences between the two organelle trees may reflect differences in the evolutionary histories and molecular evolution of plastid and mitogenomes. Compared with chloroplast genomes, plant mitogenomes are generally more structurally dynamic and are frequently shaped by repeat-mediated recombination, gene loss, rearrangement, and variable rates of sequence evolution. Therefore, the combined use of chloroplast and mitochondrial phylogenetic analyses provides a more balanced view of organelle-level evolution in *S. plebeia* and related Lamiaceae species.

Although this study provides a high-quality organelle genomic resource for *S. plebeia*, several limitations should be acknowledged. First, the mitogenome is represented as an assembled circular-mapping molecule, whereas plant mitochondrial DNA may exist as a mixture of alternative physical conformations *in vivo*. Second, although long-read mapping and PCR validation supported repeat-mediated recombination, the relative abundance, tissue specificity, and developmental dynamics of these conformations remain unclear. Third, the predicted RNA-editing sites require transcriptome-based validation, and their potential effects on mitochondrial protein function should be further examined experimentally. Fourth, while MTPTs and repeat-mediated rearrangements provide useful clues about organelle genome interaction and structural evolution, their functional consequences remain uncertain. In addition, because an assembled nuclear genome sequence of *S. plebeia* is not currently available for direct genome-wide comparison, mitochondrial-to-nuclear transferred fragments could not be systematically evaluated in this study. Future studies integrating complete nuclear, mitochondrial, chloroplast, and transcriptomic datasets will help clarify the functional and evolutionary significance of organelle genome interactions, repeat-mediated recombination, and post-transcriptional regulation in *S. plebeia*.

## Conclusion

This study assembled the mitogenome of *S. plebeia* (444, 036 bp), revealing a circular structure shaped by repetitive element-mediated recombination. While cpgenome (151, 062 bp) demonstrated exceptional conservation across Salvia species, with near-perfect synteny confirming its canonical functional architecture. Phylogenetic analyses confirmed its placement within Lamiaceae, with conserved core genes supporting respiratory function and structural rearrangements reflecting adaptive divergence. SSRs and dispersed repeats drove genomic plasticity, enabling rapid adaptation through homologous recombination. Codon usage biases (A/U-rich) and extensive RNA editing (C-to-U) optimized translational efficiency and protein stability under environmental stress. Critically, MTPTs exemplified inter-organellar crosstalk, retained tRNAs potentially augment mitochondrial translation while fragmented plastid genes decayed. These dual-genome findings advance understanding of organellar genome evolution in plants: mitochondrial plasticity facilitates ecological adaptation, whereas chloroplast stability preserves essential photosynthetic functions across the genus.

## Data Availability

The original contributions presented in the study are included in the article/**Supplementary Material**. Further inquiries can be directed to the corresponding authors.

## Glossary

ROS: reactive oxygen species
CMS: cytoplasmic male sterility
HiFi: high-fidelity
CCS: Circular Consensus Sequencing
tRNAs: Transfer RNAs
RSCU: relative synonymous codon usage
Ala: Alanine
Leu: Leucine
Thr: Threonine
Ile: Isoleucine
Asp: Aspartic acid
Met: Methionine
PCGs: protein-coding genes
GC: guanine‐cytosine
rRNA: ribosomal RNA
Val: Valine
MSA: multiple sequence alignment
Arg: Arginine
Gly: Glycine
mtDNA: mitochondrial DNA
Ser: Serine
SSRs: simple sequence repeats
bp: base pairs
NADH: reduced nicotinamide adenine dinucleotide
ATP: adenosine triphosphate
ccm: cytochrome c biogenesis genes
PCR: polymerase chain reaction
RNA: ribonucleic acid
ROS: reactive oxygen species
BOLD: Barcode of Life Data Systems
mitogenome: mitogenome
MTPTs: mitochondrial-plastid sequence transfers
DEPC: diethyl pyrocarbonate
CTAB: cetyltrimethylammonium bromide
LSC: Large Single-Copy
IRB: Inverted Repeat B
SSC: Small Single-Copy
IRA: Inverted Repeat A
Ka: Nonsynonymous substitution rate
Ks: Synonymous substitution rate
