# Supplementary material for: Organelle genome architecture of Salvia plebeia reveals mitochondrial recombination and evolutionary dynamics
Source: Front Plant Sci. 2026 Jul 9;17:1865234. doi: 10.3389/fpls.2026.1865234 (PMC13391575; doi:10.3389/fpls.2026.1865234)
Supplement: Supplementary file 1 [file Table1.docx]

**Table S1 | Comparative Analysis of Mitochondrial Gene Profiles in** ***S. plebeia* and Related Species.**

| **Order** | **Name** | **NCBI Accession** |
| --- | --- | --- |
| Solanales | *Mandragora caulescens* | PP971602.1 |
| Sapindales | *Chrysanthemum boreale* | NC_039757.1 |
|  | *Diplostephium hartwegii* | NC_034354.1 |
|  | *Taraxacum mongolicum* | NC_067879.1 |
|  | *Saussurea costus* | NC_059793.1 |
| Fabales | *Phaseolus vulgaris* | NC_045135.1 |
|  | *Robinia pseudoacacia* | MW448465.1 |
|  | *Arachis hypogaea* | MW448460.1 |
|  | *Aeschynomene indica* | NC_088526.1 |
| Rosales | *Hippophae salicifolia* | PQ875193.1 |
|  | *Cannabis sativa* | NC_029855.1 |
|  | *Rubus chingii var. suavissimus* | PQ063984.1 |
|  | *Prunus cerasoides* | PQ510804.1 |
|  | *Prunus tenella* | NC_077519.1 |
| Lamiaceae | *Scutellaria tsinyunensis* | MW553042.1 |
|  | *Scutellaria franchetiana* | NC_065026.1 |
|  | *Salvia rosmarinus* | PP992923.1 |
|  | *Salvia miltiorrhiza* | NC_023209.1 |
|  | *Platostoma chinense* | OP537517.1 |
|  | *Scutellaria barbata* | NC_065025.1 |
|  | *Pogostemon heyneanus* | MK728874.1 |
|  | *Ajuga reptans* | NC_023103.1 |
|  | *Rotheca serrata* | NC_049064.1 |
|  | *Vitex trifolia* | NC_065806.1 |
