# Supplementary material for: Organelle genome architecture of Salvia plebeia reveals mitochondrial recombination and evolutionary dynamics
Source: Front Plant Sci. 2026 Jul 9;17:1865234. doi: 10.3389/fpls.2026.1865234 (PMC13391575; doi:10.3389/fpls.2026.1865234)
Supplement: Supplementary file 2 [file Table2.docx]

**Table S2 | Sanger sequencing results of *rbcL* and *matK* genomic sequences**

***rbcL***: GGCATCACTGCTCTACATAGTTCTTAGCAGATAATCCCAATTTCGGTTTAATAGTACATCCTAATAGAGGACGCCCATACTTGTTTAATTTATCTCTTTCAACTTGAATACCATGAGGTGGTCCTTGGAAAGTTTTAACATAAGCAACAGGAATTCGCAGATCCTCCAGACGTAGAGCACGCAGGGCTTTGAACCCAAAAACATTACCCACAATGGAAGTAAACATATTAGTAACAGAACCTTCTTCAAACAGGTCTAAAGGATAAGCTACATAAGCAATATATTGATTTTCTTCGCCAGCAACGGGCTCGATGTGGTAGCATCGTCCTTTGTAACGATCAAGGCTGGTAAGTCCATCAGTCCACACAGTTGTCCATGTACCAGTAGAAGATTCAGCAGCTACTGCAGCCCCTGCTTCCTCAGGTGGAACTCCGGGTTGAGGAGTTACTCGGAATGCTGCTAAGATATCAGTATCCTTGGTTTCATACTCAGGAGTATAATAAGTCAATTTATAATCTTTAACACCAGCTTTGAATCCAACACTTGCTTTAGTCTCTGTTTGGGGTGACAATT

***matK***:

TGCGTTAGGAGGATCACTTTATACATTTAACTTCTGTGTCAGATATAGAAATAACCATTCCCATTCATCTCGAGATATTGATTCAAACCCTACGCTACTGGGTGAAAGATGCTTCTTCGTTACATTTAGTACGATTCTTTCTTTATAAGTATGATAATTGGAATAGCCTTATTCCACGAAAGAAACCTATTTCCAGTTTTTTAAACTTTAAAAGGAATCCGAAATCTTTGTTGTTCCTGTATAATTATCATGTCTACGAATCCGAATTCATTCTCGGTTTTCTTCGTAACCAATCGGTGCATTTACCATCAACCTCTTTTGGAGTCTTTTTTGAGCGAATCCACTTCTATGAAAAAAAAAAACCACTTCTTGTAAACGTGTTTTTTATTCAAACCAAGCTAAGGTTGTTCAAGGATCCGGTTATTCATTATGTTAGGTATCAAGGAAAAGTTTTTTGGGGCGCAAAAGGTACGCTTCTCCTGATGAATAAATGGAAATATTATCTTATTACTTTATGGCAATGTCATTTTTACGTGTGGGTTCAACCCAGGCGAGTTCATATAAACCAACTATCTAAACATCCCTTCGACTTTATGGGTTATTTGTCGAATCTGGGACTAATTTTTTCAGTTGTCCGGAGTCAACTATTAAACAATTCATTTATAATTAAGAATGCTATTAAGCAGTTATATATTATAGTTCCCGTTTTGTCTCTTATTGACTCATTGGCTAAAGCCAAGTTTTGTAATCTATTTGGGCATCCTATTAGTAAACCTGCCTGGACCGATTCAGCCGATTCGGATATTATCGACCGATTTGTGTGGCTATGTCGAAATCTTTCTCACTATTACAGTGGATCTTCAAAAAAAAAAAGTTTCTATCGAATAAAGTATATATTGAGACTTTCGTGGTGCTAGAACTTTGGCTCGTATAGAAATAATAGTACGATCATGCTGCGAGCGG
