# Supplementary material for: Organelle genome architecture of Salvia plebeia reveals mitochondrial recombination and evolutionary dynamics
Source: Front Plant Sci. 2026 Jul 9;17:1865234. doi: 10.3389/fpls.2026.1865234 (PMC13391575; doi:10.3389/fpls.2026.1865234)
Supplement: Supplementary file 3 [file Table3.docx]

**Table S3 | The results of BOLD database using *matK* in *S.plebeia.***

| **Query ID** | **PID [BIN]** | **Phylum** | **Class** | **Order** | **Family** | **Subfamily** | **Genus** | **Species** | **Indels** | **ID%** |
| --- | --- | --- | --- | --- | --- | --- | --- | --- | --- | --- |
| matK | GBVU4565-13 | Tracheophyta | Magnoliopsida | Lamiales | Lamiaceae |  | Salvia | Salvia plebeia | 0 | 100 |
| matK | GBVU4566-13 | Tracheophyta | Magnoliopsida | Lamiales | Lamiaceae |  | Salvia | Salvia plebeia | 0 | 100 |
| matK | GBVU4567-13 | Tracheophyta | Magnoliopsida | Lamiales | Lamiaceae |  | Salvia | Salvia plebeia | 0 | 100 |
| matK | GBVU4569-13 | Tracheophyta | Magnoliopsida | Lamiales | Lamiaceae |  | Salvia | Salvia plebeia | 0 | 100 |
| matK | GBVU4568-13 | Tracheophyta | Magnoliopsida | Lamiales | Lamiaceae |  | Salvia | Salvia plebeia | 1 | 100 |
| matK | GBVU4530-13 | Tracheophyta | Magnoliopsida | Lamiales | Lamiaceae |  | Salvia | Salvia cavaleriei var. cavaleriei | 0 | 99.38 |
| matK | GBVU4531-13 | Tracheophyta | Magnoliopsida | Lamiales | Lamiaceae |  | Salvia | Salvia cavaleriei var. simplicifolia | 0 | 99.38 |
| matK | GBVU4570-13 | Tracheophyta | Magnoliopsida | Lamiales | Lamiaceae |  | Salvia | Salvia plectranthoides | 0 | 99.38 |
| matK | GBVU4528-13 | Tracheophyta | Magnoliopsida | Lamiales | Lamiaceae |  | Salvia | Salvia brachyloma | 0 | 99.29 |
| matK | GBVU4532-13 | Tracheophyta | Magnoliopsida | Lamiales | Lamiaceae |  | Salvia | Salvia cavaleriei var. simplicifolia | 0 | 99.29 |
| matK | GBVU4537-13 | Tracheophyta | Magnoliopsida | Lamiales | Lamiaceae |  | Salvia | Salvia cynica | 0 | 99.29 |
| matK | GBVU4543-13 | Tracheophyta | Magnoliopsida | Lamiales | Lamiaceae |  | Salvia | Salvia flava | 0 | 99.29 |
| matK | GBVU4547-13 | Tracheophyta | Magnoliopsida | Lamiales | Lamiaceae |  | Salvia | Salvia kiaometiensis | 0 | 99.29 |
| matK | GBVU4571-13 | Tracheophyta | Magnoliopsida | Lamiales | Lamiaceae |  | Salvia | Salvia prattii | 0 | 99.29 |
| matK | GBVU4574-13 | Tracheophyta | Magnoliopsida | Lamiales | Lamiaceae |  | Salvia | Salvia przewalskii | 0 | 99.29 |
| matK | GBVU4576-13 | Tracheophyta | Magnoliopsida | Lamiales | Lamiaceae |  | Salvia | Salvia roborowskii | 0 | 99.29 |
| matK | GBVU4582-13 | Tracheophyta | Magnoliopsida | Lamiales | Lamiaceae |  | Salvia | Salvia tricuspis | 0 | 99.29 |
| matK | GBVU4585-13 | Tracheophyta | Magnoliopsida | Lamiales | Lamiaceae |  | Salvia | Salvia yunnanensis | 0 | 99.29 |
| matK | GBVU4542-13 | Tracheophyta | Magnoliopsida | Lamiales | Lamiaceae |  | Salvia | Salvia flava | 0 | 99.2 |
| matK | BPTPS141-22 | Tracheophyta | Magnoliopsida | Asterales | Asteraceae |  | Helichrysum | Helichrysum italicum | 6 | 97.46 |
| matK | BPTPS108-22 | Tracheophyta | Magnoliopsida | Lamiales | Lamiaceae | Nepetoideae | Melissa | Melissa officinalis | 6 | 97.46 |
| matK | BPTPS081-22 | Tracheophyta | Magnoliopsida | Lamiales | Lamiaceae |  | Salvia | Salvia officinalis | 6 | 96.69 |
| matK | BPTPS130-22 | Tracheophyta | Magnoliopsida | Lamiales | Lamiaceae |  | Salvia | Salvia officinalis | 6 | 96.69 |
| matK | BPTPS149-22 | Tracheophyta | Magnoliopsida | Lamiales | Lamiaceae |  | Salvia | Salvia officinalis | 6 | 96.69 |
| matK | BPTPS182-22 | Tracheophyta | Magnoliopsida | Lamiales | Lamiaceae |  | Salvia | Salvia officinalis | 6 | 96.69 |
