# Supplementary material for: Organelle genome architecture of Salvia plebeia reveals mitochondrial recombination and evolutionary dynamics
Source: Front Plant Sci. 2026 Jul 9;17:1865234. doi: 10.3389/fpls.2026.1865234 (PMC13391575; doi:10.3389/fpls.2026.1865234)
Supplement: Supplementary file 4 [file Table4.docx]

**Table S4 | The results of BOLD database using *rbcL* in *S.plebeia.***

| **Query ID** | **PID [BIN]** | **Phylum** | **Class** | **Order** | **Family** | **Subfamily** | **Genus** | **Species** | **Indels** | **ID%** |
| --- | --- | --- | --- | --- | --- | --- | --- | --- | --- | --- |
| rbcL | GBVG3444-11 | Tracheophyta | Magnoliopsida | Lamiales | Lamiaceae |  | Salvia | Salvia japonica var. japonica | 0 | 99.54 |
| rbcL | GBVG3445-11 | Tracheophyta | Magnoliopsida | Lamiales | Lamiaceae |  | Salvia | Salvia japonica var. japonica | 0 | 99.54 |
| rbcL | GBVG3452-11 | Tracheophyta | Magnoliopsida | Lamiales | Lamiaceae |  | Salvia | Salvia lutescens var. lutescens | 0 | 99.38 |
| rbcL | GBVG3421-11 | Tracheophyta | Magnoliopsida | Lamiales | Lamiaceae |  | Salvia | Salvia glabrescens | 0 | 99.31 |
| rbcL | GBVG3467-11 | Tracheophyta | Magnoliopsida | Lamiales | Lamiaceae |  | Salvia | Salvia nipponica | 0 | 99.31 |
| rbcL | GBVG3468-11 | Tracheophyta | Magnoliopsida | Lamiales | Lamiaceae |  | Salvia | Salvia nipponica | 0 | 99.31 |
| rbcL | BPTPS029-22 | Tracheophyta | Magnoliopsida | Lamiales | Lamiaceae |  | Salvia | Salvia rosmarinus | 0 | 99.15 |
| rbcL | BPTPS049-22 | Tracheophyta | Magnoliopsida | Lamiales | Lamiaceae |  | Salvia | Salvia rosmarinus | 0 | 99.15 |
| rbcL | BPTPS081-22 | Tracheophyta | Magnoliopsida | Lamiales | Lamiaceae |  | Salvia | Salvia officinalis | 0 | 99 |
| rbcL | BPTPS130-22 | Tracheophyta | Magnoliopsida | Lamiales | Lamiaceae |  | Salvia | Salvia officinalis | 0 | 99 |
| rbcL | BPTPS149-22 | Tracheophyta | Magnoliopsida | Lamiales | Lamiaceae |  | Salvia | Salvia officinalis | 0 | 99 |
| rbcL | BPTPS182-22 | Tracheophyta | Magnoliopsida | Lamiales | Lamiaceae |  | Salvia | Salvia officinalis | 0 | 99 |
| rbcL | BPTPS014-22 | Tracheophyta | Magnoliopsida | Lamiales | Lamiaceae | Nepetoideae | Clinopodium | Clinopodium menthifolium | 0 | 98.77 |
| rbcL | BPTPS105-22 | Tracheophyta | Magnoliopsida | Lamiales | Lamiaceae | Nepetoideae | Clinopodium | Clinopodium menthifolium | 0 | 98.77 |
| rbcL | BPTPS168-22 | Tracheophyta | Magnoliopsida | Lamiales | Lamiaceae | Nepetoideae | Clinopodium | Clinopodium menthifolium | 0 | 98.77 |
| rbcL | GBVG3076-11 | Tracheophyta | Magnoliopsida | Lamiales | Lamiaceae | Nepetoideae | Melissa | Melissa officinalis | 0 | 98.69 |
| rbcL | BPTPS144-22 | Tracheophyta | Magnoliopsida | Lamiales | Lamiaceae | Nepetoideae | Mentha | Mentha aquatica | 0 | 98.69 |
| rbcL | BPTPS157-22 | Tracheophyta | Magnoliopsida | Lamiales | Lamiaceae | Nepetoideae | Mentha | Mentha aquatica | 0 | 98.69 |
| rbcL | BPTPS163-22 | Tracheophyta | Magnoliopsida | Lamiales | Lamiaceae | Nepetoideae | Mentha | Mentha cervina | 0 | 98.69 |
| rbcL | BPTPS147-22 | Tracheophyta | Magnoliopsida | Lamiales | Lamiaceae | Nepetoideae | Mentha | Mentha spicata | 0 | 98.69 |
| rbcL | BPTPS177-22 | Tracheophyta | Magnoliopsida | Lamiales | Lamiaceae | Nepetoideae | Mentha | Mentha spicata | 0 | 98.69 |
| rbcL | BPTPS143-22 | Tracheophyta | Magnoliopsida | Lamiales | Lamiaceae | Nepetoideae | Mentha | Mentha suaveolens | 0 | 98.69 |
| rbcL | BPTPS153-22 | Tracheophyta | Magnoliopsida | Lamiales | Lamiaceae | Nepetoideae | Mentha | Mentha x piperita | 0 | 98.69 |
| rbcL | BPTPS158-22 | Tracheophyta | Magnoliopsida | Lamiales | Lamiaceae |  | Satureja | Satureja montana | 0 | 98.69 |
| rbcL | BPTPS172-22 | Tracheophyta | Magnoliopsida | Lamiales | Lamiaceae | Nepetoideae | Thymus | Thymus caespititius | 0 | 98.69 |
