# Supplementary material for: Organelle genome architecture of Salvia plebeia reveals mitochondrial recombination and evolutionary dynamics
Source: Front Plant Sci. 2026 Jul 9;17:1865234. doi: 10.3389/fpls.2026.1865234 (PMC13391575; doi:10.3389/fpls.2026.1865234)
Supplement: Supplementary file 5 [file Table5.docx]

**Table S5 | Relative synonymous codon usage of the mitochondrial genome of *S. plebeia.***

| **Amino** | **Codon 1 RSCU** | **Codon 2 RSCU** | **Codon 3 RSCU** | **Codon 4 RSCU** | **Codon 5 RSCU** | **Codon 6 RSCU** |
| --- | --- | --- | --- | --- | --- | --- |
| Ala | GCA(0.983) | GCC(0.9471) | GCG(0.6484) | GCU(1.4215) |  |  |
| Arg | AGA(1.7323) | AGG(1.3148) | CGA(0.9578) | CGC(0.5967) | CGG(0.7483) | CGU(0.65) |
| Asn | AAC(0.8576) | AAU(1.1424) |  |  |  |  |
| Asp | GAC(0.7852) | GAU(1.2148) |  |  |  |  |
| Cys | UGC(0.995) | UGU(1.005) |  |  |  |  |
| Gln | CAA(1.1823) | CAG(0.8177) |  |  |  |  |
| Glu | GAA(1.207) | GAG(0.793) |  |  |  |  |
| Gly | GGA(1.3049) | GGC(0.84) | GGG(0.9468) | GGU(0.9082) |  |  |
| His | CAC(0.8109) | CAU(1.1891) |  |  |  |  |
| Ile | AUA(0.9628) | AUC(0.9437) | AUU(1.0935) |  |  |  |
| Leu | CUA(0.9338) | CUC(0.9464) | CUG(0.6702) | CUU(1.5162) | UUA(0.8677) | UUG(1.0657) |
| Lys | AAA(1.0171) | AAG(0.9829) |  |  |  |  |
| Met | AUG(1) |  |  |  |  |  |
| Phe | UUC(0.9652) | UUU(1.0348) |  |  |  |  |
| Pro | CCA(0.9966) | CCC(0.9854) | CCG(0.7252) | CCU(1.2928) |  |  |
| Ser | AGC(0.9304) | AGU(0.9213) | UCA(0.9899) | UCC(1.0116) | UCG(0.7207) | UCU(1.4261) |
| Ter | UAA(0.9906) | UAG(0.9874) | UGA(1.022) |  |  |  |
| Thr | ACA(0.9669) | ACC(1.0599) | ACG(0.6515) |  |  |  |
| Trp | UGG(1) |  |  |  |  |  |
| Tyr | UAC(0.8531) | UAU(1.1469) |  |  |  |  |
| Val | GUA(1.0275) | GUC(0.8975) | GUG(0.757) | GUU(1.3179) |  |  |
