# Supplementary material for: Organelle genome architecture of Salvia plebeia reveals mitochondrial recombination and evolutionary dynamics
Source: Front Plant Sci. 2026 Jul 9;17:1865234. doi: 10.3389/fpls.2026.1865234 (PMC13391575; doi:10.3389/fpls.2026.1865234)
Supplement: Supplementary file 6 [file Table6.docx]

**Table S6 | Simple sequence repeats in the mitochondrial genome of *S. plebeia.***

| **ID** | **SSR type** | **SSR** | **size** | **start** | **end** |
| --- | --- | --- | --- | --- | --- |
| mtDNA | p1 | (T)14 | 14 | 4684 | 4697 |
| mtDNA | p5 | (TTTAT)3 | 15 | 8635 | 8649 |
| mtDNA | p4 | (CCTC)3 | 12 | 12874 | 12885 |
| mtDNA | c | (CTTTT)3...(CCTG)3 | 790 | 15864 | 16653 |
| mtDNA | c | (TAAA)3...(T)10 | 1044 | 20694 | 21737 |
| mtDNA | p5 | (AATAT)3 | 15 | 22741 | 22755 |
| mtDNA | p2 | (AG)5 | 10 | 25933 | 25942 |
| mtDNA | c | (CAATA)3...(CTGG)3 | 1224 | 27386 | 28609 |
| mtDNA | c | (AG)5...(AAGA)3 | 580 | 32581 | 33160 |
| mtDNA | p4 | (CCTT)3 | 12 | 35207 | 35218 |
| mtDNA | p4 | (GAAG)3 | 12 | 39281 | 39292 |
| mtDNA | p2 | (AG)5 | 10 | 40430 | 40439 |
| mtDNA | p4 | (GCCG)3 | 12 | 43624 | 43635 |
| mtDNA | c | (AATAA)3...(CCATA)3 | 1679 | 45149 | 46827 |
| mtDNA | c | (CTT)4...(TTC)5 | 136 | 54606 | 54741 |
| mtDNA | p5 | (CTTAT)3 | 15 | 64754 | 64768 |
| mtDNA | c | (CTA)4...(TCTT)3 | 1034 | 66957 | 67990 |
| mtDNA | p4 | (CTAT)3 | 12 | 74335 | 74346 |
| mtDNA | p3 | (AAC)4 | 12 | 79317 | 79328 |
| mtDNA | c | (GAAG)3...(AT)5 | 472 | 82245 | 82716 |
| mtDNA | c | (T)10...(CCTT)3 | 891 | 85937 | 86827 |
| mtDNA | p4 | (TGTT)3 | 12 | 90906 | 90917 |
| mtDNA | c | (A)14...(AAGAG)3 | 979 | 93659 | 94637 |
| mtDNA | p4 | (TCTT)3 | 12 | 97794 | 97805 |
| mtDNA | p4 | (TCTT)3 | 12 | 98952 | 98963 |
| mtDNA | p4 | (GAGC)3 | 12 | 105694 | 105705 |
| mtDNA | c | (CTAT)3...(AAAG)3 | 611 | 116557 | 117167 |
| mtDNA | p2 | (AG)5 | 10 | 119184 | 119193 |
| mtDNA | c | (TTATA)3...(TTTC)3 | 90 | 122382 | 122471 |
| mtDNA | p5 | (ACTAG)3 | 15 | 128524 | 128538 |
| mtDNA | c | (A)10...(AG)5 | 359 | 131169 | 131527 |
| mtDNA | p4 | (GAAT)3 | 12 | 134782 | 134793 |
| mtDNA | p3 | (ATA)4 | 12 | 138487 | 138498 |
| mtDNA | p1 | (T)11 | 11 | 140715 | 140725 |
| mtDNA | c | (T)10...(T)11 | 1004 | 142707 | 143710 |
| mtDNA | p2 | (TA)5 | 10 | 146434 | 146443 |
| mtDNA | p4 | (CTAT)3 | 12 | 148292 | 148303 |
| mtDNA | p4 | (GAAA)3 | 12 | 155852 | 155863 |
| mtDNA | p2 | (TC)5 | 10 | 178651 | 178660 |
| mtDNA | p3 | (TAT)4 | 12 | 183945 | 183956 |
| mtDNA | p5 | (ATTTT)3 | 15 | 190937 | 190951 |
| mtDNA | p1 | (A)11 | 11 | 195889 | 195899 |
| mtDNA | c | (AATAA)3...(TC)5 | 178 | 198987 | 199164 |
| mtDNA | p3 | (TAG)4 | 12 | 201579 | 201590 |
| mtDNA | p3 | (GTC)4 | 12 | 204142 | 204153 |
| mtDNA | p2 | (TA)5 | 10 | 206684 | 206693 |
| mtDNA | c | (TTTAA)3...(ATA)4 | 1333 | 211388 | 212720 |
| mtDNA | p1 | (A)10 | 10 | 215836 | 215845 |
| mtDNA | p3 | (TAG)4 | 12 | 219049 | 219060 |
| mtDNA | p1 | (A)12 | 12 | 228028 | 228039 |
| mtDNA | c | (CTTA)3...(CTT)4 | 752 | 238118 | 238869 |
| mtDNA | p2 | (AG)6 | 12 | 242210 | 242221 |
| mtDNA | p2 | (TC)5 | 10 | 250079 | 250088 |
| mtDNA | p4 | (TTCT)3 | 12 | 254801 | 254812 |
| mtDNA | p4 | (ATTG)3 | 12 | 262007 | 262018 |
| mtDNA | c | (TTA)4...(AAGA)3 | 89 | 267103 | 267191 |
| mtDNA | c | (CCAA)3...(ATA)4 | 345 | 271945 | 272289 |
| mtDNA | p4 | (CATT)3 | 12 | 273470 | 273481 |
| mtDNA | p1 | (T)10 | 10 | 277115 | 277124 |
| mtDNA | p4 | (GGTC)3 | 12 | 281925 | 281936 |
| mtDNA | p3 | (AAT)5 | 15 | 283254 | 283268 |
| mtDNA | c | (AAGA)3...(GAA)4 | 493 | 285275 | 285767 |
| mtDNA | p2 | (TA)5 | 10 | 294015 | 294024 |
| mtDNA | p4 | (AAAG)3 | 12 | 296690 | 296701 |
| mtDNA | c | (AC)5...(TTTA)3 | 779 | 299521 | 300299 |
| mtDNA | p3 | (TAT)4 | 12 | 305248 | 305259 |
| mtDNA | p1 | (A)10 | 10 | 310127 | 310136 |
| mtDNA | p4 | (GAAA)3 | 12 | 317175 | 317186 |
| mtDNA | p1 | (T)10 | 10 | 318191 | 318200 |
| mtDNA | p4 | (AAAG)3 | 12 | 320593 | 320604 |
| mtDNA | p4 | (GAAA)3 | 12 | 321614 | 321625 |
| mtDNA | c | (ATT)4...(CTCTAA)3 | 790 | 323139 | 323928 |
| mtDNA | c | (AT)5...(AAAG)3 | 954 | 331850 | 332803 |
| mtDNA | p4 | (TATC)3 | 12 | 337071 | 337082 |
| mtDNA | p2 | (AG)5 | 10 | 339958 | 339967 |
| mtDNA | p4 | (AAAG)3 | 12 | 341813 | 341824 |
| mtDNA | p1 | (T)10 | 10 | 346945 | 346954 |
| mtDNA | p5 | (ATTTT)3 | 15 | 349241 | 349255 |
| mtDNA | c | (TC)5(AG)5 | 639 | 352604 | 353242 |
| mtDNA | c | (TCTT)3...(TTTC)3 | 893 | 360451 | 361343 |
| mtDNA | c | (TATTC)3...(TTCT)3 | 262 | 365136 | 365397 |
| mtDNA | p4 | (TCTG)3 | 12 | 368929 | 368940 |
| mtDNA | p2 | (AG)5 | 10 | 374476 | 374485 |
| mtDNA | p2 | (AG)5 | 10 | 394437 | 394446 |
| mtDNA | p4 | (AAGA)3 | 12 | 397379 | 397390 |
| mtDNA | p4 | (TAGA)3 | 12 | 398551 | 398562 |
| mtDNA | p5 | (TTATA)3 | 15 | 401621 | 401635 |
| mtDNA | p3 | (AAT)4 | 12 | 406470 | 406481 |
| mtDNA | p1 | (A)10 | 10 | 407498 | 407507 |
| mtDNA | p5 | (TAAAG)3 | 15 | 413418 | 413432 |
| mtDNA | p3 | (GAA)4 | 12 | 418846 | 418857 |
| mtDNA | p4 | (CAAA)3 | 12 | 420519 | 420530 |
| mtDNA | p2 | (TA)6 | 12 | 421976 | 421987 |
| mtDNA | c | (CTTT)3...(GCTT)3 | 1391 | 423072 | 424462 |
| mtDNA | p4 | (AATG)3 | 12 | 428460 | 428471 |
| mtDNA | p5 | (TAATA)3 | 15 | 430491 | 430505 |
| mtDNA | c | (AAGA)3...(TCT)4 | 598 | 432211 | 432808 |
| mtDNA | p4 | (AGTG)3 | 12 | 435705 | 435716 |
| mtDNA | p4 | (GACA)3 | 12 | 441743 | 441754 |
