# Supplementary material for: Organelle genome architecture of Salvia plebeia reveals mitochondrial recombination and evolutionary dynamics
Source: Front Plant Sci. 2026 Jul 9;17:1865234. doi: 10.3389/fpls.2026.1865234 (PMC13391575; doi:10.3389/fpls.2026.1865234)
Supplement: Supplementary file 7 [file Table7.docx]

**Table S7 | Dispersed repeats in *S. plebeia* mitogenome.**

| **The Chr that the first part located** | **The repeat length of the first part** | **The start site of the first part** | **Type** | **The Chr that the second part located** | **The repeat length of the second part** | **The start site of the second part** | **Interval distance of repeats** | **E-value** |
| --- | --- | --- | --- | --- | --- | --- | --- | --- |
| mtDNA | 6111 | 366648 | P | mtDNA | 6111 | 437925 | 0 | 0.00E+00 |
| mtDNA | 4627 | 189574 | F | mtDNA | 4627 | 347878 | 0 | 0.00E+00 |
| mtDNA | 2406 | 249708 | P | mtDNA | 2406 | 392411 | 0 | 0.00E+00 |
| mtDNA | 1076 | 111792 | F | mtDNA | 1076 | 433184 | 0 | 0.00E+00 |
| mtDNA | 445 | 4736 | P | mtDNA | 445 | 72687 | 0 | 6.72E-258 |
| mtDNA | 194 | 110009 | P | mtDNA | 194 | 343744 | 0 | 8.80E-107 |
| mtDNA | 148 | 118071 | F | mtDNA | 148 | 299276 | 0 | 4.36E-79 |
| mtDNA | 142 | 136798 | P | mtDNA | 142 | 189432 | 0 | 1.78E-75 |
| mtDNA | 120 | 72395 | F | mtDNA | 120 | 427042 | -1 | 1.13E-59 |
| mtDNA | 116 | 103964 | P | mtDNA | 116 | 213822 | -1 | 2.80E-57 |
| mtDNA | 111 | 63050 | P | mtDNA | 111 | 220417 | -2 | 4.52E-52 |
| mtDNA | 98 | 71598 | F | mtDNA | 98 | 431302 | 0 | 5.52E-49 |
| mtDNA | 109 | 109550 | P | mtDNA | 109 | 135708 | -3 | 7.46E-49 |
| mtDNA | 97 | 133621 | F | mtDNA | 97 | 223164 | 0 | 2.21E-48 |
| mtDNA | 93 | 72422 | F | mtDNA | 93 | 427069 | 0 | 5.65E-46 |
| mtDNA | 90 | 370226 | P | mtDNA | 90 | 426190 | 0 | 3.62E-44 |
| mtDNA | 90 | 426190 | F | mtDNA | 90 | 440368 | 0 | 3.62E-44 |
| mtDNA | 89 | 215964 | F | mtDNA | 89 | 255320 | 0 | 1.45E-43 |
| mtDNA | 88 | 66145 | P | mtDNA | 88 | 116916 | -1 | 1.53E-40 |
| mtDNA | 88 | 133552 | P | mtDNA | 88 | 367819 | -2 | 1.99E-38 |
| mtDNA | 88 | 133552 | F | mtDNA | 88 | 442777 | -2 | 1.99E-38 |
| mtDNA | 80 | 226942 | P | mtDNA | 80 | 332988 | -1 | 9.11E-36 |
| mtDNA | 72 | 132668 | P | mtDNA | 72 | 193636 | 0 | 2.49E-33 |
| mtDNA | 72 | 132668 | P | mtDNA | 72 | 351940 | 0 | 2.49E-33 |
| mtDNA | 75 | 136278 | P | mtDNA | 75 | 226062 | -1 | 8.74E-33 |
| mtDNA | 71 | 79459 | P | mtDNA | 71 | 353911 | 0 | 9.95E-33 |
| mtDNA | 78 | 109581 | P | mtDNA | 78 | 135708 | -2 | 1.64E-32 |
| mtDNA | 73 | 133567 | P | mtDNA | 73 | 367819 | -1 | 1.36E-31 |
| mtDNA | 73 | 133567 | F | mtDNA | 73 | 442792 | -1 | 1.36E-31 |
| mtDNA | 67 | 9637 | P | mtDNA | 67 | 238118 | 0 | 2.55E-30 |
| mtDNA | 66 | 46381 | F | mtDNA | 66 | 85009 | -1 | 2.02E-27 |
| mtDNA | 65 | 208330 | P | mtDNA | 65 | 329784 | -1 | 7.94E-27 |
| mtDNA | 60 | 46381 | P | mtDNA | 60 | 377372 | 0 | 4.17E-26 |
| mtDNA | 60 | 146567 | F | mtDNA | 60 | 156369 | 0 | 4.17E-26 |
| mtDNA | 67 | 112401 | P | mtDNA | 67 | 325607 | -2 | 5.07E-26 |
| mtDNA | 67 | 325607 | P | mtDNA | 67 | 433793 | -2 | 5.07E-26 |
| mtDNA | 62 | 41318 | P | mtDNA | 62 | 429545 | -1 | 4.85E-25 |
| mtDNA | 61 | 85008 | P | mtDNA | 61 | 377372 | -1 | 1.91E-24 |
| mtDNA | 57 | 208338 | P | mtDNA | 57 | 329784 | 0 | 2.67E-24 |
| mtDNA | 59 | 398216 | F | mtDNA | 59 | 404320 | -1 | 2.95E-23 |
| mtDNA | 52 | 46395 | F | mtDNA | 52 | 85023 | 0 | 2.73E-21 |
| mtDNA | 52 | 71680 | P | mtDNA | 52 | 328415 | 0 | 2.73E-21 |
| mtDNA | 58 | 36942 | F | mtDNA | 58 | 121297 | -2 | 9.93E-21 |
| mtDNA | 53 | 67115 | F | mtDNA | 53 | 148740 | -1 | 1.09E-19 |
| mtDNA | 53 | 109606 | P | mtDNA | 53 | 135708 | -1 | 1.09E-19 |
| mtDNA | 53 | 325621 | P | mtDNA | 53 | 433793 | -1 | 1.09E-19 |
| mtDNA | 49 | 227077 | P | mtDNA | 49 | 277954 | 0 | 1.75E-19 |
| mtDNA | 52 | 293095 | F | mtDNA | 52 | 380739 | -1 | 4.27E-19 |
| mtDNA | 51 | 109639 | F | mtDNA | 51 | 398223 | -1 | 1.67E-18 |
| mtDNA | 47 | 32573 | F | mtDNA | 47 | 40422 | 0 | 2.80E-18 |
| mtDNA | 47 | 104033 | P | mtDNA | 47 | 213822 | 0 | 2.80E-18 |
| mtDNA | 47 | 112862 | F | mtDNA | 47 | 254810 | 0 | 2.80E-18 |
| mtDNA | 47 | 343503 | P | mtDNA | 47 | 347783 | 0 | 2.80E-18 |
| mtDNA | 46 | 85023 | P | mtDNA | 46 | 377372 | 0 | 1.12E-17 |
| mtDNA | 49 | 186999 | F | mtDNA | 49 | 291455 | -1 | 2.57E-17 |
| mtDNA | 45 | 145630 | P | mtDNA | 45 | 167187 | 0 | 4.48E-17 |
| mtDNA | 51 | 109639 | F | mtDNA | 51 | 404327 | -2 | 1.25E-16 |
| mtDNA | 51 | 228401 | F | mtDNA | 51 | 328256 | -2 | 1.25E-16 |
| mtDNA | 44 | 192521 | P | mtDNA | 44 | 254534 | 0 | 1.79E-16 |
| mtDNA | 44 | 254534 | P | mtDNA | 44 | 350825 | 0 | 1.79E-16 |
| mtDNA | 53 | 75093 | P | mtDNA | 53 | 275537 | -3 | 4.32E-16 |
| mtDNA | 50 | 178738 | F | mtDNA | 50 | 186604 | -2 | 4.82E-16 |
| mtDNA | 43 | 55884 | F | mtDNA | 43 | 403486 | 0 | 7.17E-16 |
| mtDNA | 43 | 103439 | P | mtDNA | 43 | 146977 | 0 | 7.17E-16 |
| mtDNA | 43 | 118160 | F | mtDNA | 43 | 303788 | 0 | 7.17E-16 |
| mtDNA | 43 | 299365 | F | mtDNA | 43 | 303788 | 0 | 7.17E-16 |
| mtDNA | 42 | 38137 | P | mtDNA | 42 | 38854 | 0 | 2.87E-15 |
| mtDNA | 51 | 46396 | F | mtDNA | 51 | 321413 | -3 | 6.15E-15 |
| mtDNA | 51 | 85024 | F | mtDNA | 51 | 321413 | -3 | 6.15E-15 |
| mtDNA | 51 | 190756 | F | mtDNA | 51 | 404053 | -3 | 6.15E-15 |
| mtDNA | 51 | 349060 | F | mtDNA | 51 | 404053 | -3 | 6.15E-15 |
| mtDNA | 41 | 109649 | F | mtDNA | 41 | 398233 | 0 | 1.15E-14 |
| mtDNA | 50 | 213866 | P | mtDNA | 50 | 398135 | -3 | 2.31E-14 |
| mtDNA | 40 | 135816 | P | mtDNA | 40 | 365343 | 0 | 4.59E-14 |
| mtDNA | 40 | 397374 | F | mtDNA | 40 | 432206 | 0 | 4.59E-14 |
| mtDNA | 49 | 133651 | P | mtDNA | 49 | 193949 | -3 | 8.70E-14 |
| mtDNA | 49 | 133651 | P | mtDNA | 49 | 352253 | -3 | 8.70E-14 |
| mtDNA | 49 | 193949 | P | mtDNA | 49 | 223194 | -3 | 8.70E-14 |
| mtDNA | 49 | 223194 | P | mtDNA | 49 | 352253 | -3 | 8.70E-14 |
| mtDNA | 43 | 151042 | F | mtDNA | 43 | 164688 | -1 | 9.25E-14 |
| mtDNA | 46 | 79883 | P | mtDNA | 46 | 372547 | -2 | 1.04E-13 |
| mtDNA | 46 | 79883 | F | mtDNA | 46 | 438091 | -2 | 1.04E-13 |
| mtDNA | 39 | 181573 | F | mtDNA | 39 | 336013 | 0 | 1.83E-13 |
| mtDNA | 45 | 169877 | P | mtDNA | 45 | 374914 | -2 | 3.99E-13 |
| mtDNA | 45 | 396672 | F | mtDNA | 45 | 430780 | -2 | 3.99E-13 |
| mtDNA | 41 | 109649 | F | mtDNA | 41 | 404337 | -1 | 1.41E-12 |
| mtDNA | 44 | 133637 | P | mtDNA | 44 | 193968 | -2 | 1.53E-12 |
| mtDNA | 44 | 133637 | P | mtDNA | 44 | 352272 | -2 | 1.53E-12 |
| mtDNA | 44 | 193968 | P | mtDNA | 44 | 223180 | -2 | 1.53E-12 |
| mtDNA | 44 | 223180 | P | mtDNA | 44 | 352272 | -2 | 1.53E-12 |
| mtDNA | 37 | 208425 | F | mtDNA | 37 | 380921 | 0 | 2.94E-12 |
| mtDNA | 37 | 325637 | P | mtDNA | 37 | 433793 | 0 | 2.94E-12 |
| mtDNA | 46 | 103986 | F | mtDNA | 46 | 398135 | -3 | 4.59E-12 |
| mtDNA | 46 | 213877 | P | mtDNA | 46 | 398128 | -3 | 4.59E-12 |
| mtDNA | 40 | 226902 | P | mtDNA | 40 | 333067 | -1 | 5.50E-12 |
| mtDNA | 43 | 185542 | F | mtDNA | 43 | 203751 | -2 | 5.82E-12 |
| mtDNA | 45 | 321413 | P | mtDNA | 45 | 377372 | -3 | 1.72E-11 |
| mtDNA | 39 | 82801 | F | mtDNA | 39 | 118153 | -1 | 2.15E-11 |
| mtDNA | 39 | 82801 | F | mtDNA | 39 | 299358 | -1 | 2.15E-11 |
| mtDNA | 42 | 85034 | P | mtDNA | 42 | 200401 | -2 | 2.22E-11 |
| mtDNA | 42 | 150957 | F | mtDNA | 42 | 167343 | -2 | 2.22E-11 |
| mtDNA | 35 | 36965 | F | mtDNA | 35 | 121320 | 0 | 4.70E-11 |
| mtDNA | 35 | 55975 | P | mtDNA | 35 | 105452 | 0 | 4.70E-11 |
| mtDNA | 35 | 154346 | P | mtDNA | 35 | 365439 | 0 | 4.70E-11 |
| mtDNA | 35 | 362753 | P | mtDNA | 35 | 430707 | 0 | 4.70E-11 |
| mtDNA | 44 | 46873 | P | mtDNA | 44 | 256871 | -3 | 6.41E-11 |
| mtDNA | 44 | 99561 | P | mtDNA | 44 | 436881 | -3 | 6.41E-11 |
| mtDNA | 44 | 200402 | P | mtDNA | 44 | 321420 | -3 | 6.41E-11 |
| mtDNA | 41 | 9752 | P | mtDNA | 41 | 403558 | -2 | 8.46E-11 |
| mtDNA | 41 | 46406 | P | mtDNA | 41 | 200402 | -2 | 8.46E-11 |
| mtDNA | 41 | 71671 | F | mtDNA | 41 | 365152 | -2 | 8.46E-11 |
| mtDNA | 34 | 135629 | P | mtDNA | 34 | 397099 | 0 | 1.88E-10 |
| mtDNA | 34 | 226868 | P | mtDNA | 34 | 333101 | 0 | 1.88E-10 |
| mtDNA | 34 | 332600 | P | mtDNA | 34 | 402791 | 0 | 1.88E-10 |
| mtDNA | 43 | 36050 | F | mtDNA | 43 | 240866 | -3 | 2.39E-10 |
| mtDNA | 43 | 99411 | P | mtDNA | 43 | 366271 | -3 | 2.39E-10 |
| mtDNA | 43 | 113481 | F | mtDNA | 43 | 288132 | -3 | 2.39E-10 |
| mtDNA | 37 | 44839 | P | mtDNA | 37 | 432548 | -1 | 3.26E-10 |
| mtDNA | 37 | 75109 | P | mtDNA | 37 | 275537 | -1 | 3.26E-10 |
| mtDNA | 33 | 66504 | F | mtDNA | 33 | 306488 | 0 | 7.52E-10 |
| mtDNA | 33 | 254064 | F | mtDNA | 33 | 260351 | 0 | 7.52E-10 |
| mtDNA | 33 | 329835 | F | mtDNA | 33 | 333266 | 0 | 7.52E-10 |
| mtDNA | 39 | 154453 | F | mtDNA | 39 | 407638 | -2 | 1.22E-09 |
| mtDNA | 36 | 55953 | F | mtDNA | 36 | 204459 | -1 | 1.27E-09 |
| mtDNA | 36 | 370180 | P | mtDNA | 36 | 426279 | -1 | 1.27E-09 |
| mtDNA | 36 | 426279 | F | mtDNA | 36 | 440468 | -1 | 1.27E-09 |
| mtDNA | 32 | 46947 | P | mtDNA | 32 | 256813 | 0 | 3.01E-09 |
| mtDNA | 32 | 74370 | F | mtDNA | 32 | 75031 | 0 | 3.01E-09 |
| mtDNA | 41 | 259931 | P | mtDNA | 41 | 342415 | -3 | 3.30E-09 |
| mtDNA | 41 | 366356 | P | mtDNA | 41 | 399918 | -3 | 3.30E-09 |
| mtDNA | 38 | 81199 | F | mtDNA | 38 | 334724 | -2 | 4.64E-09 |
| mtDNA | 35 | 20934 | F | mtDNA | 35 | 108499 | -1 | 4.93E-09 |
| mtDNA | 31 | 66202 | P | mtDNA | 31 | 116916 | 0 | 1.20E-08 |
| mtDNA | 31 | 95199 | F | mtDNA | 31 | 200824 | 0 | 1.20E-08 |
| mtDNA | 31 | 144462 | F | mtDNA | 31 | 176625 | 0 | 1.20E-08 |
| mtDNA | 31 | 151054 | F | mtDNA | 31 | 164700 | 0 | 1.20E-08 |
| mtDNA | 31 | 333259 | P | mtDNA | 31 | 404316 | 0 | 1.20E-08 |
| mtDNA | 40 | 35337 | F | mtDNA | 40 | 419077 | -3 | 1.22E-08 |
| mtDNA | 40 | 63359 | P | mtDNA | 40 | 75174 | -3 | 1.22E-08 |
| mtDNA | 40 | 280754 | F | mtDNA | 40 | 429541 | -3 | 1.22E-08 |
| mtDNA | 40 | 294734 | P | mtDNA | 40 | 417100 | -3 | 1.22E-08 |
| mtDNA | 37 | 149104 | F | mtDNA | 37 | 221452 | -2 | 1.76E-08 |
| mtDNA | 34 | 35211 | P | mtDNA | 34 | 228296 | -1 | 1.92E-08 |
| mtDNA | 34 | 88381 | P | mtDNA | 34 | 422956 | -1 | 1.92E-08 |
| mtDNA | 34 | 131469 | P | mtDNA | 34 | 204592 | -1 | 1.92E-08 |
| mtDNA | 34 | 208382 | F | mtDNA | 34 | 380876 | -1 | 1.92E-08 |
| mtDNA | 39 | 910 | P | mtDNA | 39 | 294852 | -3 | 4.53E-08 |
| mtDNA | 30 | 62072 | F | mtDNA | 30 | 285498 | 0 | 4.81E-08 |
| mtDNA | 30 | 83224 | P | mtDNA | 30 | 344094 | 0 | 4.81E-08 |
| mtDNA | 30 | 154308 | P | mtDNA | 30 | 365467 | 0 | 4.81E-08 |
| mtDNA | 30 | 194097 | F | mtDNA | 30 | 369542 | 0 | 4.81E-08 |
| mtDNA | 30 | 194097 | P | mtDNA | 30 | 441112 | 0 | 4.81E-08 |
| mtDNA | 30 | 352401 | F | mtDNA | 30 | 369542 | 0 | 4.81E-08 |
| mtDNA | 30 | 352401 | P | mtDNA | 30 | 441112 | 0 | 4.81E-08 |
| mtDNA | 30 | 397203 | F | mtDNA | 30 | 397980 | 0 | 4.81E-08 |
| mtDNA | 38 | 46822 | P | mtDNA | 38 | 256927 | -3 | 1.67E-07 |
| mtDNA | 38 | 136877 | P | mtDNA | 38 | 208644 | -3 | 1.67E-07 |
| mtDNA | 38 | 153485 | P | mtDNA | 38 | 161038 | -3 | 1.67E-07 |
| mtDNA | 38 | 161030 | P | mtDNA | 38 | 400192 | -3 | 1.67E-07 |
| mtDNA | 38 | 189457 | F | mtDNA | 38 | 208644 | -3 | 1.67E-07 |
| mtDNA | 35 | 39309 | P | mtDNA | 35 | 387075 | -2 | 2.52E-07 |
| mtDNA | 35 | 200408 | F | mtDNA | 35 | 377372 | -2 | 2.52E-07 |
| mtDNA | 32 | 54611 | P | mtDNA | 32 | 369771 | -1 | 2.89E-07 |
| mtDNA | 32 | 54611 | F | mtDNA | 32 | 440881 | -1 | 2.89E-07 |
| mtDNA | 32 | 71354 | F | mtDNA | 32 | 191263 | -1 | 2.89E-07 |
| mtDNA | 32 | 71354 | F | mtDNA | 32 | 349567 | -1 | 2.89E-07 |
| mtDNA | 32 | 82808 | F | mtDNA | 32 | 303788 | -1 | 2.89E-07 |
| mtDNA | 37 | 161021 | P | mtDNA | 37 | 400202 | -3 | 6.16E-07 |
| mtDNA | 37 | 169928 | P | mtDNA | 37 | 374871 | -3 | 6.16E-07 |
| mtDNA | 37 | 213894 | P | mtDNA | 37 | 398120 | -3 | 6.16E-07 |
| mtDNA | 37 | 223394 | F | mtDNA | 37 | 341539 | -3 | 6.16E-07 |
| mtDNA | 37 | 369873 | F | mtDNA | 37 | 384501 | -3 | 6.16E-07 |
| mtDNA | 37 | 384501 | P | mtDNA | 37 | 440774 | -3 | 6.16E-07 |
| mtDNA | 34 | 150798 | F | mtDNA | 34 | 335138 | -2 | 9.49E-07 |
| mtDNA | 34 | 382897 | F | mtDNA | 34 | 430189 | -2 | 9.49E-07 |
| mtDNA | 31 | 51701 | F | mtDNA | 31 | 112475 | -1 | 1.12E-06 |
| mtDNA | 31 | 51701 | F | mtDNA | 31 | 433867 | -1 | 1.12E-06 |
| mtDNA | 31 | 79900 | P | mtDNA | 31 | 372545 | -1 | 1.12E-06 |
| mtDNA | 31 | 79900 | F | mtDNA | 31 | 438108 | -1 | 1.12E-06 |
| mtDNA | 31 | 108903 | P | mtDNA | 31 | 213579 | -1 | 1.12E-06 |
| mtDNA | 36 | 33849 | P | mtDNA | 36 | 367928 | -3 | 2.26E-06 |
| mtDNA | 36 | 33849 | F | mtDNA | 36 | 442720 | -3 | 2.26E-06 |
| mtDNA | 36 | 109820 | P | mtDNA | 36 | 436788 | -3 | 2.26E-06 |
| mtDNA | 36 | 154372 | P | mtDNA | 36 | 211953 | -3 | 2.26E-06 |
| mtDNA | 36 | 219310 | P | mtDNA | 36 | 253676 | -3 | 2.26E-06 |
| mtDNA | 33 | 39588 | P | mtDNA | 33 | 109560 | -2 | 3.57E-06 |
| mtDNA | 33 | 63104 | F | mtDNA | 33 | 156462 | -2 | 3.57E-06 |
| mtDNA | 33 | 156462 | P | mtDNA | 33 | 220441 | -2 | 3.57E-06 |
| mtDNA | 33 | 400616 | P | mtDNA | 33 | 402886 | -2 | 3.57E-06 |
| mtDNA | 30 | 0 | P | mtDNA | 30 | 304458 | -1 | 4.33E-06 |
| mtDNA | 30 | 14698 | P | mtDNA | 30 | 71586 | -1 | 4.33E-06 |
| mtDNA | 30 | 44698 | P | mtDNA | 30 | 398216 | -1 | 4.33E-06 |
| mtDNA | 30 | 44698 | P | mtDNA | 30 | 404320 | -1 | 4.33E-06 |
| mtDNA | 30 | 49484 | P | mtDNA | 30 | 228247 | -1 | 4.33E-06 |
| mtDNA | 30 | 66157 | F | mtDNA | 30 | 306468 | -1 | 4.33E-06 |
| mtDNA | 30 | 105641 | P | mtDNA | 30 | 325543 | -1 | 4.33E-06 |
| mtDNA | 30 | 109612 | F | mtDNA | 30 | 404302 | -1 | 4.33E-06 |
| mtDNA | 30 | 116962 | P | mtDNA | 30 | 306468 | -1 | 4.33E-06 |
| mtDNA | 30 | 134059 | P | mtDNA | 30 | 293259 | -1 | 4.33E-06 |
| mtDNA | 30 | 135725 | P | mtDNA | 30 | 404302 | -1 | 4.33E-06 |
| mtDNA | 30 | 150969 | F | mtDNA | 30 | 167355 | -1 | 4.33E-06 |
| mtDNA | 30 | 156501 | P | mtDNA | 30 | 202873 | -1 | 4.33E-06 |
| mtDNA | 30 | 258046 | P | mtDNA | 30 | 384628 | -1 | 4.33E-06 |
| mtDNA | 30 | 333259 | P | mtDNA | 30 | 398213 | -1 | 4.33E-06 |
| mtDNA | 35 | 29218 | P | mtDNA | 35 | 187771 | -3 | 8.30E-06 |
| mtDNA | 35 | 76049 | F | mtDNA | 35 | 372627 | -3 | 8.30E-06 |
| mtDNA | 35 | 76049 | P | mtDNA | 35 | 438022 | -3 | 8.30E-06 |
| mtDNA | 35 | 190839 | F | mtDNA | 35 | 404136 | -3 | 8.30E-06 |
| mtDNA | 35 | 255463 | F | mtDNA | 35 | 333145 | -3 | 8.30E-06 |
| mtDNA | 35 | 349143 | F | mtDNA | 35 | 404136 | -3 | 8.30E-06 |
| mtDNA | 32 | 10633 | P | mtDNA | 32 | 86232 | -2 | 1.34E-05 |
| mtDNA | 32 | 35337 | F | mtDNA | 32 | 308512 | -2 | 1.34E-05 |
| mtDNA | 32 | 61688 | F | mtDNA | 32 | 65116 | -2 | 1.34E-05 |
| mtDNA | 32 | 63367 | P | mtDNA | 32 | 75174 | -2 | 1.34E-05 |
| mtDNA | 32 | 99573 | P | mtDNA | 32 | 436881 | -2 | 1.34E-05 |
| mtDNA | 32 | 156461 | F | mtDNA | 32 | 275973 | -2 | 1.34E-05 |
| mtDNA | 32 | 219957 | F | mtDNA | 32 | 306109 | -2 | 1.34E-05 |
| mtDNA | 32 | 264183 | F | mtDNA | 32 | 298672 | -2 | 1.34E-05 |
| mtDNA | 32 | 328435 | P | mtDNA | 32 | 365161 | -2 | 1.34E-05 |
| mtDNA | 34 | 35335 | F | mtDNA | 34 | 435683 | -3 | 3.04E-05 |
| mtDNA | 34 | 48827 | F | mtDNA | 34 | 377241 | -3 | 3.04E-05 |
| mtDNA | 34 | 59661 | F | mtDNA | 34 | 249179 | -3 | 3.04E-05 |
| mtDNA | 34 | 117759 | P | mtDNA | 34 | 150179 | -3 | 3.04E-05 |
| mtDNA | 34 | 153183 | P | mtDNA | 34 | 366581 | -3 | 3.04E-05 |
| mtDNA | 34 | 181432 | P | mtDNA | 34 | 411943 | -3 | 3.04E-05 |
| mtDNA | 34 | 190781 | F | mtDNA | 34 | 404078 | -3 | 3.04E-05 |
| mtDNA | 34 | 308519 | F | mtDNA | 34 | 435692 | -3 | 3.04E-05 |
| mtDNA | 34 | 349085 | F | mtDNA | 34 | 404078 | -3 | 3.04E-05 |
| mtDNA | 31 | 1845 | F | mtDNA | 31 | 79327 | -2 | 5.03E-05 |
| mtDNA | 31 | 82072 | F | mtDNA | 31 | 250294 | -2 | 5.03E-05 |
| mtDNA | 31 | 82072 | P | mtDNA | 31 | 394200 | -2 | 5.03E-05 |
| mtDNA | 31 | 200415 | P | mtDNA | 31 | 321420 | -2 | 5.03E-05 |
| mtDNA | 31 | 246694 | F | mtDNA | 31 | 295703 | -2 | 5.03E-05 |
| mtDNA | 31 | 366174 | F | mtDNA | 31 | 368501 | -2 | 5.03E-05 |
| mtDNA | 31 | 366174 | P | mtDNA | 31 | 442152 | -2 | 5.03E-05 |
| mtDNA | 33 | 8103 | P | mtDNA | 33 | 156445 | -3 | 1.11E-04 |
| mtDNA | 33 | 35344 | F | mtDNA | 33 | 435692 | -3 | 1.11E-04 |
| mtDNA | 33 | 39588 | F | mtDNA | 33 | 135774 | -3 | 1.11E-04 |
| mtDNA | 33 | 102113 | P | mtDNA | 33 | 298907 | -3 | 1.11E-04 |
| mtDNA | 33 | 163711 | F | mtDNA | 33 | 398429 | -3 | 1.11E-04 |
| mtDNA | 33 | 190854 | F | mtDNA | 33 | 404151 | -3 | 1.11E-04 |
| mtDNA | 33 | 238165 | F | mtDNA | 33 | 377411 | -3 | 1.11E-04 |
| mtDNA | 33 | 349158 | F | mtDNA | 33 | 404151 | -3 | 1.11E-04 |
| mtDNA | 30 | 8076 | P | mtDNA | 30 | 156479 | -2 | 1.88E-04 |
| mtDNA | 30 | 33932 | F | mtDNA | 30 | 133579 | -2 | 1.88E-04 |
| mtDNA | 30 | 33932 | P | mtDNA | 30 | 367850 | -2 | 1.88E-04 |
| mtDNA | 30 | 33932 | F | mtDNA | 30 | 442804 | -2 | 1.88E-04 |
| mtDNA | 30 | 47010 | P | mtDNA | 30 | 256744 | -2 | 1.88E-04 |
| mtDNA | 30 | 105428 | P | mtDNA | 30 | 384776 | -2 | 1.88E-04 |
| mtDNA | 30 | 112439 | P | mtDNA | 30 | 325606 | -2 | 1.88E-04 |
| mtDNA | 30 | 113585 | F | mtDNA | 30 | 288162 | -2 | 1.88E-04 |
| mtDNA | 30 | 154307 | F | mtDNA | 30 | 422485 | -2 | 1.88E-04 |
| mtDNA | 30 | 163722 | F | mtDNA | 30 | 398440 | -2 | 1.88E-04 |
| mtDNA | 30 | 222568 | P | mtDNA | 30 | 252839 | -2 | 1.88E-04 |
| mtDNA | 30 | 226915 | P | mtDNA | 30 | 333064 | -2 | 1.88E-04 |
| mtDNA | 30 | 248741 | F | mtDNA | 30 | 336948 | -2 | 1.88E-04 |
| mtDNA | 30 | 280764 | F | mtDNA | 30 | 429551 | -2 | 1.88E-04 |
| mtDNA | 30 | 294604 | F | mtDNA | 30 | 295853 | -2 | 1.88E-04 |
| mtDNA | 30 | 329833 | P | mtDNA | 30 | 404312 | -2 | 1.88E-04 |
| mtDNA | 30 | 355172 | P | mtDNA | 30 | 404389 | -2 | 1.88E-04 |
| mtDNA | 30 | 377409 | F | mtDNA | 30 | 379049 | -2 | 1.88E-04 |
| mtDNA | 32 | 791 | P | mtDNA | 32 | 337443 | -3 | 4.03E-04 |
| mtDNA | 32 | 55905 | F | mtDNA | 32 | 210579 | -3 | 4.03E-04 |
| mtDNA | 32 | 68566 | P | mtDNA | 32 | 80869 | -3 | 4.03E-04 |
| mtDNA | 32 | 75122 | F | mtDNA | 32 | 298843 | -3 | 4.03E-04 |
| mtDNA | 32 | 90223 | F | mtDNA | 32 | 97389 | -3 | 4.03E-04 |
| mtDNA | 32 | 90893 | F | mtDNA | 32 | 123068 | -3 | 4.03E-04 |
| mtDNA | 32 | 109600 | F | mtDNA | 32 | 380761 | -3 | 4.03E-04 |
| mtDNA | 32 | 130470 | F | mtDNA | 32 | 205213 | -3 | 4.03E-04 |
| mtDNA | 32 | 135735 | P | mtDNA | 32 | 380761 | -3 | 4.03E-04 |
| mtDNA | 32 | 207506 | P | mtDNA | 32 | 261030 | -3 | 4.03E-04 |
| mtDNA | 32 | 265575 | P | mtDNA | 32 | 337649 | -3 | 4.03E-04 |
| mtDNA | 32 | 283669 | P | mtDNA | 32 | 288310 | -3 | 4.03E-04 |
| mtDNA | 32 | 284479 | P | mtDNA | 32 | 308511 | -3 | 4.03E-04 |
| mtDNA | 32 | 304177 | P | mtDNA | 32 | 423406 | -3 | 4.03E-04 |
| mtDNA | 32 | 321398 | F | mtDNA | 32 | 412470 | -3 | 4.03E-04 |
| mtDNA | 32 | 341823 | F | mtDNA | 32 | 366151 | -3 | 4.03E-04 |
| mtDNA | 32 | 365152 | F | mtDNA | 32 | 431375 | -3 | 4.03E-04 |
| mtDNA | 31 | 264 | P | mtDNA | 31 | 304037 | -3 | 1.46E-03 |
| mtDNA | 31 | 1305 | F | mtDNA | 31 | 336939 | -3 | 1.46E-03 |
| mtDNA | 31 | 4078 | F | mtDNA | 31 | 4093 | -3 | 1.46E-03 |
| mtDNA | 31 | 35348 | F | mtDNA | 31 | 419088 | -3 | 1.46E-03 |
| mtDNA | 31 | 41349 | P | mtDNA | 31 | 280758 | -3 | 1.46E-03 |
| mtDNA | 31 | 46831 | P | mtDNA | 31 | 256925 | -3 | 1.46E-03 |
| mtDNA | 31 | 47922 | F | mtDNA | 31 | 366208 | -3 | 1.46E-03 |
| mtDNA | 31 | 80913 | F | mtDNA | 31 | 117748 | -3 | 1.46E-03 |
| mtDNA | 31 | 85004 | P | mtDNA | 31 | 379046 | -3 | 1.46E-03 |
| mtDNA | 31 | 100777 | F | mtDNA | 31 | 304023 | -3 | 1.46E-03 |
| mtDNA | 31 | 105417 | F | mtDNA | 31 | 195319 | -3 | 1.46E-03 |
| mtDNA | 31 | 121697 | P | mtDNA | 31 | 260157 | -3 | 1.46E-03 |
| mtDNA | 31 | 248106 | F | mtDNA | 31 | 354105 | -3 | 1.46E-03 |
| mtDNA | 31 | 284594 | F | mtDNA | 31 | 367100 | -3 | 1.46E-03 |
| mtDNA | 31 | 284594 | P | mtDNA | 31 | 443553 | -3 | 1.46E-03 |
| mtDNA | 31 | 325556 | F | mtDNA | 31 | 339692 | -3 | 1.46E-03 |
| mtDNA | 31 | 328358 | P | mtDNA | 31 | 431450 | -3 | 1.46E-03 |
| mtDNA | 31 | 365468 | P | mtDNA | 31 | 422484 | -3 | 1.46E-03 |
| mtDNA | 30 | 731 | F | mtDNA | 30 | 416791 | -3 | 5.27E-03 |
| mtDNA | 30 | 924 | P | mtDNA | 30 | 294847 | -3 | 5.27E-03 |
| mtDNA | 30 | 12065 | F | mtDNA | 30 | 298018 | -3 | 5.27E-03 |
| mtDNA | 30 | 12447 | P | mtDNA | 30 | 352815 | -3 | 5.27E-03 |
| mtDNA | 30 | 14650 | P | mtDNA | 30 | 208362 | -3 | 5.27E-03 |
| mtDNA | 30 | 14650 | F | mtDNA | 30 | 329787 | -3 | 5.27E-03 |
| mtDNA | 30 | 46768 | P | mtDNA | 30 | 256976 | -3 | 5.27E-03 |
| mtDNA | 30 | 80437 | P | mtDNA | 30 | 149426 | -3 | 5.27E-03 |
| mtDNA | 30 | 81123 | F | mtDNA | 30 | 336830 | -3 | 5.27E-03 |
| mtDNA | 30 | 105401 | F | mtDNA | 30 | 195303 | -3 | 5.27E-03 |
| mtDNA | 30 | 105643 | P | mtDNA | 30 | 339677 | -3 | 5.27E-03 |
| mtDNA | 30 | 108911 | P | mtDNA | 30 | 213572 | -3 | 5.27E-03 |
| mtDNA | 30 | 109685 | P | mtDNA | 30 | 355172 | -3 | 5.27E-03 |
| mtDNA | 30 | 113337 | P | mtDNA | 30 | 317582 | -3 | 5.27E-03 |
| mtDNA | 30 | 135401 | P | mtDNA | 30 | 398484 | -3 | 5.27E-03 |
| mtDNA | 30 | 146906 | P | mtDNA | 30 | 197845 | -3 | 5.27E-03 |
| mtDNA | 30 | 147099 | F | mtDNA | 30 | 328853 | -3 | 5.27E-03 |
| mtDNA | 30 | 149783 | F | mtDNA | 30 | 305635 | -3 | 5.27E-03 |
| mtDNA | 30 | 153493 | F | mtDNA | 30 | 400192 | -3 | 5.27E-03 |
| mtDNA | 30 | 159762 | F | mtDNA | 30 | 213389 | -3 | 5.27E-03 |
| mtDNA | 30 | 161891 | P | mtDNA | 30 | 400776 | -3 | 5.27E-03 |
| mtDNA | 30 | 169936 | P | mtDNA | 30 | 374870 | -3 | 5.27E-03 |
| mtDNA | 30 | 178762 | F | mtDNA | 30 | 186628 | -3 | 5.27E-03 |
| mtDNA | 30 | 191208 | F | mtDNA | 30 | 347874 | -3 | 5.27E-03 |
| mtDNA | 30 | 206814 | F | mtDNA | 30 | 423428 | -3 | 5.27E-03 |
| mtDNA | 30 | 212896 | P | mtDNA | 30 | 264119 | -3 | 5.27E-03 |
| mtDNA | 30 | 252634 | P | mtDNA | 30 | 416010 | -3 | 5.27E-03 |
| mtDNA | 30 | 277385 | P | mtDNA | 30 | 411948 | -3 | 5.27E-03 |
| mtDNA | 30 | 285484 | P | mtDNA | 30 | 414939 | -3 | 5.27E-03 |
| mtDNA | 30 | 287416 | P | mtDNA | 30 | 287872 | -3 | 5.27E-03 |
| mtDNA | 30 | 316629 | F | mtDNA | 30 | 422298 | -3 | 5.27E-03 |
| mtDNA | 30 | 334800 | F | mtDNA | 30 | 337214 | -3 | 5.27E-03 |
| mtDNA | 30 | 347874 | F | mtDNA | 30 | 349512 | -3 | 5.27E-03 |
