# Supplementary material for: Organelle genome architecture of Salvia plebeia reveals mitochondrial recombination and evolutionary dynamics
Source: Front Plant Sci. 2026 Jul 9;17:1865234. doi: 10.3389/fpls.2026.1865234 (PMC13391575; doi:10.3389/fpls.2026.1865234)
Supplement: Supplementary file 8 [file Table8.docx]

**Table S8 | Tandem repeats in *S. plebeia* mitogenome*.***

| **Chr** | **Indices** | **Period Size** | **Copy Number** | **Consensus Size** | **Percent Matches** | **Percent Indels** | **Score** | **A** | **C** | **G** | **T** | **Entropy(0-2)** |
| --- | --- | --- | --- | --- | --- | --- | --- | --- | --- | --- | --- | --- |
| mtDNA | 4084--4124 | 15 | 2.7 | 15 | 96 | 0 | 73 | 17 | 21 | 0 | 60 | 1.35 |
| mtDNA | 44780--44832 | 21 | 2.5 | 21 | 71 | 0 | 52 | 49 | 20 | 13 | 16 | 1.79 |
| mtDNA | 116453--116482 | 13 | 2.2 | 14 | 94 | 5 | 53 | 50 | 16 | 26 | 6 | 1.7 |
| mtDNA | 220696--220735 | 21 | 2 | 19 | 90 | 9 | 62 | 40 | 15 | 20 | 25 | 1.9 |
| mtDNA | 257482--257513 | 16 | 2 | 16 | 93 | 0 | 55 | 37 | 15 | 21 | 25 | 1.93 |
