# Supplementary material for: Organelle genome architecture of Salvia plebeia reveals mitochondrial recombination and evolutionary dynamics
Source: Front Plant Sci. 2026 Jul 9;17:1865234. doi: 10.3389/fpls.2026.1865234 (PMC13391575; doi:10.3389/fpls.2026.1865234)
Supplement: Supplementary file 9 [file Table9.docx]

**Table S9 | BLASTn results among mitogenome and cpgenome.**

| **ID** | **query id** | **subject id** | **identity** | **alignment length** | **mismatches** | **gap openings** | **q.start** | **q.end** | **s.start** | **s.end** | **evalue** | **bit score** | **Gene in cpDNA** | **Gene in mtDNA** |
| --- | --- | --- | --- | --- | --- | --- | --- | --- | --- | --- | --- | --- | --- | --- |
| MTPT1 | cpDNA | mtDNA | 98.387 | 62 | 1 | 0 | 23770 | 23831 | 237707 | 237646 | 4.91E-23 | 110 | trnA-UGC |  |
| MTPT2 | cpDNA | mtDNA | 98.387 | 62 | 1 | 0 | 144732 | 144793 | 237646 | 237707 | 4.91E-23 | 110 | trnA-UGC |  |
| MTPT3 | cpDNA | mtDNA | 93.75 | 80 | 5 | 0 | 94447 | 94526 | 388665 | 388744 | 2.27E-26 | 121 | trnM-CAU | trnM-CAU |
| MTPT4 | cpDNA | mtDNA | 97.531 | 81 | 2 | 0 | 43063 | 43143 | 242818 | 242898 | 6.27E-32 | 139 | rpl23 | trnH-GUG |
| MTPT5 | cpDNA | mtDNA | 94.048 | 84 | 4 | 1 | 18892 | 18974 | 178328 | 178245 | 4.88E-28 | 126 | trnN-GUU | trnN-GUU |
| MTPT6 | cpDNA | mtDNA | 94.048 | 84 | 4 | 1 | 149589 | 149671 | 178245 | 178328 | 4.88E-28 | 126 | trnR-ACG | trnR-ACG |
| MTPT7 | cpDNA | mtDNA | 78.889 | 90 | 16 | 3 | 51240 | 51328 | 30755 | 30668 | 1.80E-07 | 58.4 | trnS-GCU | trnS-GGA |
| MTPT8 | cpDNA | mtDNA | 94.783 | 115 | 6 | 0 | 87477 | 87591 | 30654 | 30768 | 3.69E-44 | 180 | trnS-GGA | trnS-GGA |
| MTPT9 | cpDNA | mtDNA | 90.076 | 131 | 12 | 1 | 72756 | 72885 | 30190 | 30060 | 7.99E-41 | 169 | psbM | trnD-GUC |
| MTPT10 | cpDNA | mtDNA | 90.278 | 144 | 8 | 2 | 72017 | 72154 | 257062 | 257205 | 2.85E-45 | 183 |  |  |
| MTPT11 | cpDNA | mtDNA | 94.304 | 158 | 2 | 2 | 98762 | 98918 | 13992 | 14143 | 7.77E-61 | 235 | rbcl |  |
| MTPT12 | cpDNA | mtDNA | 89.266 | 177 | 8 | 6 | 134751 | 134924 | 37340 | 37172 | 1.31E-53 | 211 | ycf15 |  |
| MTPT13 | cpDNA | mtDNA | 89.266 | 177 | 8 | 6 | 33639 | 33812 | 37172 | 37340 | 1.31E-53 | 211 | ycf15 |  |
| MTPT14 | cpDNA | mtDNA | 87.64 | 178 | 11 | 4 | 88149 | 88326 | 30774 | 30940 | 3.67E-49 | 196 | rps4 |  |
| MTPT15 | cpDNA | mtDNA | 98.131 | 214 | 4 | 0 | 11949 | 12162 | 255828 | 255615 | 1.58E-102 | 374 | ndhA、ndhH |  |
| MTPT16 | cpDNA | mtDNA | 90.361 | 249 | 5 | 6 | 101052 | 101289 | 257442 | 257202 | 4.51E-83 | 309 |  |  |
| MTPT17 | cpDNA | mtDNA | 91.831 | 355 | 22 | 2 | 43773 | 44126 | 419359 | 419707 | 5.40E-137 | 488 | psbA |  |
| MTPT18 | cpDNA | mtDNA | 96.546 | 637 | 3 | 4 | 80784 | 81415 | 262773 | 263395 | 0 | 1037 | psaB |  |
| MTPT19 | cpDNA | mtDNA | 73.732 | 887 | 177 | 40 | 26027 | 26890 | 155518 | 154665 | 3.51E-79 | 296 | rrn16S | rrn18、trnP-CGG |
| MTPT20 | cpDNA | mtDNA | 73.732 | 887 | 177 | 40 | 141673 | 142536 | 154665 | 155518 | 3.51E-79 | 296 | rrn16S | rrn18、trnP-CGG |
| MTPT21 | cpDNA | mtDNA | 99.322 | 885 | 6 | 0 | 100158 | 101042 | 258441 | 257557 | 0 | 1602 |  |  |
| MTPT22 | cpDNA | mtDNA | 96.787 | 996 | 7 | 7 | 86105 | 87076 | 347387 | 346393 | 0 | 1639 | ycf3 |  |
| MTPT23 | cpDNA | mtDNA | 98 | 1050 | 13 | 5 | 107206 | 108248 | 224518 | 225566 | 0 | 1816 | petL、petG、trnW-CCA | trnW-CCA |
| MTPT24 | cpDNA | mtDNA | 95.539 | 1771 | 21 | 11 | 66297 | 68050 | 269112 | 270841 | 0 | 2780 |  |  |
| MTPT25 | cpDNA | mtDNA | 99.534 | 2144 | 5 | 1 | 62562 | 64700 | 332084 | 329941 | 0 | 3899 |  |  |
