# Supplementary material for: Organelle genome architecture of Salvia plebeia reveals mitochondrial recombination and evolutionary dynamics
Source: Front Plant Sci. 2026 Jul 9;17:1865234. doi: 10.3389/fpls.2026.1865234 (PMC13391575; doi:10.3389/fpls.2026.1865234)
Supplement: Supplementary file 10 [file Table10.docx]

**Table S10 | RNA editing sites prediction in *S. plebia* mitogenome*.***

| **Gene** | **Base** | **Aa** | **Triplet pos.** | **Bases** | **Codon** | **Aa change** |
| --- | --- | --- | --- | --- | --- | --- |
| matR | 44 | 15 | 2 | C→U | CCC→CUC | P→L |
| matR | 326 | 109 | 2 | C→U | CCA→CUA | P→L |
| matR | 413 | 138 | 2 | C→U | UCG→UUG | S→L |
| matR | 688 | 230 | 1 | C→U | CUC→UUC | L→F |
| matR | 1198 | 400 | 1 | C→U | CUU→UUU | L→F |
| matR | 1430 | 477 | 2 | C→U | GCA→GUA | A→V |
| matR | 1679 | 560 | 2 | C→U | UCC→UUC | S→F |
| matR | 1700 | 567 | 2 | C→U | CCU→CUU | P→L |
| matR | 1720 | 574 | 1 | C→U | CGC→UGC | R→C |
| matR | 1756 | 586 | 1 | C→U | CAC→UAC | H→Y |
| matR | 1787 | 596 | 2 | C→U | CCG→CUG | P→L |
| matR | 1826 | 609 | 2 | C→U | CCA→CUA | P→L |
| matR | 1844 | 615 | 2 | C→U | UCA→UUA | S→L |
| nad1 | 215 | 72 | 2 | C→U | UCC→UUC | S→F |
| nad1 | 265 | 89 | 1 | C→U | CGG→UGG | R→W |
| nad1 | 308 | 103 | 2 | C→U | CCG→CUG | P→L |
| nad1 | 376 | 126 | 1 | C→U | CGG→UGG | R→W |
| nad1 | 725 | 242 | 2 | C→U | CCA→CUA | P→L |
| nad1 | 734 | 245 | 2 | C→U | UCG→UUG | S→L |
| nad1 | 740 | 247 | 2 | C→U | UCU→UUU | S→F |
| nad1 | 743 | 248 | 2 | C→U | CCA→CUA | P→L |
| nad1 | 755 | 252 | 2 | C→U | CCG→CUG | P→L |
| nad1 | 779 | 260 | 2 | C→U | UCC→UUC | S→F |
| nad1 | 823 | 275 | 1 | C→U | CUC→UUC | L→F |
| nad1 | 898 | 300 | 1 | C→U | CGG→UGG | R→W |
| nad1 | 928 | 310 | 1 | C→U | CGG→UGG | R→W |
| nad1 | 490 | 164 | 1 | C→U | CCC→UCC | P→S |
| nad1 | 493 | 165 | 1 | C→U | CGU→UGU | R→C |
| nad1 | 500 | 167 | 2 | C→U | UCG→UUG | S→L |
| nad1 | 536 | 179 | 2 | C→U | UCC→UUC | S→F |
| nad1 | 635 | 212 | 2 | C→U | UCA→UUA | S→L |
| nad1 | 761 | 254 | 2 | C→U | ACU→AUU | T→I |
| nad1 | 764 | 255 | 2 | C→U | UCA→UUA | S→L |
| nad1 | 401 | 134 | 2 | C→U | UCU→UUU | S→F |
| nad1 | 436 | 146 | 1 | C→U | CCU→UCU | P→S |
| ccmC | 5 | 2 | 2 | C→U | UCC→UUC | S→F |
| ccmC | 76 | 26 | 1 | C→U | CGG→UGG | R→W |
| ccmC | 103 | 35 | 1 | C→U | CAU→UAU | H→Y |
| ccmC | 115 | 39 | 1 | C→U | CGG→UGG | R→W |
| ccmC | 133 | 45 | 1 | C→U | CUU→UUU | L→F |
| ccmC | 161 | 54 | 2 | C→U | CCC→CUC | P→L |
| ccmC | 179 | 60 | 2 | C→U | GCG→GUG | A→V |
| ccmC | 184 | 62 | 1 | C→U | CGG→UGG | R→W |
| ccmC | 209 | 70 | 2 | C→U | GCU→GUU | A→V |
| ccmC | 281 | 94 | 2 | C→U | ACA→AUA | T→I |
| ccmC | 331 | 111 | 1 | C→U | CGG→UGG | R→W |
| ccmC | 400 | 134 | 1 | C→U | CUU→UUU | L→F |
| ccmC | 436 | 146 | 1 | C→U | CCU→UCU | P→S |
| ccmC | 446 | 149 | 2 | C→U | CCG→CUG | P→L |
| ccmC | 451 | 151 | 1 | C→U | CCU→UCU | P→S |
| ccmC | 458 | 153 | 2 | C→U | UCA→UUA | S→L |
| ccmC | 463 | 155 | 1 | C→U | CGU→UGU | R→C |
| ccmC | 467 | 156 | 2 | C→U | GCU→GUU | A→V |
| ccmC | 473 | 158 | 2 | C→U | CCG→CUG | P→L |
| ccmC | 497 | 166 | 2 | C→U | UCU→UUU | S→F |
| ccmC | 521 | 174 | 2 | C→U | UCG→UUG | S→L |
| ccmC | 568 | 190 | 1 | C→U | CCU→UCU | P→S |
| ccmC | 575 | 192 | 2 | C→U | CCC→CUC | P→L |
| ccmC | 605 | 202 | 2 | C→U | UCC→UUC | S→F |
| ccmC | 608 | 203 | 2 | C→U | CCC→CUC | P→L |
| ccmC | 614 | 205 | 2 | C→U | UCA→UUA | S→L |
| ccmC | 650 | 217 | 2 | C→U | CCU→CUU | P→L |
| ccmC | 656 | 219 | 2 | C→U | CCA→CUA | P→L |
| ccmC | 673 | 225 | 1 | C→U | CCU→UCU | P→S |
| atp6 | 418 | 140 | 1 | C→U | CCC→UCC | P→S |
| atp6 | 514 | 172 | 1 | C→U | CAU→UAU | H→Y |
| atp6 | 596 | 199 | 2 | C→U | CCG→CUG | P→L |
| atp6 | 665 | 222 | 2 | C→U | UCG→UUG | S→L |
| atp6 | 683 | 228 | 2 | C→U | UCG→UUG | S→L |
| atp6 | 691 | 231 | 1 | C→U | CGU→UGU | R→C |
| atp6 | 830 | 277 | 2 | C→U | UCA→UUA | S→L |
| atp6 | 889 | 297 | 1 | C→U | CCU→UCU | P→S |
| atp6 | 892 | 298 | 1 | C→U | CAU→UAU | H→Y |
| cob | 53 | 18 | 2 | C→U | ACA→AUA | T→I |
| cob | 325 | 109 | 1 | C→U | CAU→UAU | H→Y |
| cob | 358 | 120 | 1 | C→U | CGG→UGG | R→W |
| cob | 568 | 190 | 1 | C→U | CAU→UAU | H→Y |
| cob | 853 | 285 | 1 | C→U | CAU→UAU | H→Y |
| cob | 908 | 303 | 2 | C→U | UCA→UUA | S→L |
| cob | 982 | 328 | 1 | C→U | CAC→UAC | H→Y |
| cob | 1015 | 339 | 1 | C→U | CGC→UGC | R→C |
| cob | 1084 | 362 | 1 | C→U | CCU→UCU | P→S |
| rps14 | 305 | 102 | 2 | C→U | ACA→AUA | T→I |
| rpl5 | 35 | 12 | 2 | C→U | UCA→UUA | S→L |
| rpl5 | 47 | 16 | 2 | C→U | CCG→CUG | P→L |
| rpl5 | 64 | 22 | 1 | C→U | CAC→UAC | H→Y |
| rpl5 | 160 | 54 | 1 | C→U | CCG→UCG | P→S |
| rpl5 | 509 | 170 | 2 | C→U | CCA→CUA | P→L |
| rpl5 | 512 | 171 | 2 | C→U | CCG→CUG | P→L |
| nad2 | 788 | 263 | 2 | C→U | UCU→UUU | S→F |
| nad2 | 800 | 267 | 2 | C→U | UCA→UUA | S→L |
| nad2 | 809 | 270 | 2 | C→U | UCU→UUU | S→F |
| nad2 | 928 | 310 | 1 | C→U | CAU→UAU | H→Y |
| nad2 | 958 | 320 | 1 | C→U | CGU→UGU | R→C |
| nad2 | 1028 | 343 | 2 | C→U | UCA→UUA | S→L |
| nad2 | 1058 | 353 | 2 | C→U | UCA→UUA | S→L |
| nad2 | 1276 | 426 | 1 | C→U | CGU→UGU | R→C |
| nad2 | 223 | 75 | 1 | C→U | CUU→UUU | L→F |
| nad2 | 308 | 103 | 2 | C→U | UCU→UUU | S→F |
| nad2 | 311 | 104 | 2 | C→U | UCC→UUC | S→F |
| nad2 | 356 | 119 | 2 | C→U | CCA→CUA | P→L |
| nad2 | 361 | 121 | 1 | C→U | CCU→UCU | P→S |
| nad2 | 367 | 123 | 1 | C→U | CGC→UGC | R→C |
| nad2 | 401 | 134 | 2 | C→U | UCA→UUA | S→L |
| nad2 | 428 | 143 | 2 | C→U | CCU→CUU | P→L |
| nad2 | 497 | 166 | 2 | C→U | UCG→UUG | S→L |
| nad2 | 1298 | 433 | 2 | C→U | GCG→GUG | A→V |
| nad2 | 1400 | 467 | 2 | C→U | UCA→UUA | S→L |
| nad2 | 1409 | 470 | 2 | C→U | CCA→CUA | P→L |
| nad2 | 1457 | 486 | 2 | C→U | UCA→UUA | S→L |
| nad2 | 26 | 9 | 2 | C→U | UCC→UUC | S→F |
| rps12 | 104 | 35 | 2 | C→U | CCG→CUG | P→L |
| rps12 | 196 | 66 | 1 | C→U | CAC→UAC | H→Y |
| rps12 | 221 | 74 | 2 | C→U | UCG→UUG | S→L |
| rps12 | 284 | 95 | 2 | C→U | UCC→UUC | S→F |
| rps12 | 104 | 35 | 2 | C→U | CCG→CUG | P→L |
| atp4 | 59 | 20 | 2 | C→U | UCU→UUU | S→F |
| atp4 | 71 | 24 | 2 | C→U | UCA→UUA | S→L |
| atp4 | 89 | 30 | 2 | C→U | UCA→UUA | S→L |
| atp4 | 215 | 72 | 2 | C→U | UCG→UUG | S→L |
| atp4 | 227 | 76 | 2 | C→U | CCC→CUC | P→L |
| atp4 | 248 | 83 | 2 | C→U | CCU→CUU | P→L |
| atp4 | 251 | 84 | 2 | C→U | CCG→CUG | P→L |
| atp4 | 395 | 132 | 2 | C→U | UCA→UUA | S→L |
| atp4 | 407 | 136 | 2 | C→U | CCA→CUA | P→L |
| atp4 | 416 | 139 | 2 | C→U | ACU→AUU | T→I |
| atp4 | 455 | 152 | 2 | C→U | ACA→AUA | T→I |
| atp4 | 470 | 157 | 2 | C→U | UCA→UUA | S→L |
| atp4 | 523 | 175 | 1 | C→U | CUC→UUC | L→F |
| nad4L | 2 | 1 | 2 | C→U | ACG→AUG | T→M |
| nad4L | 8 | 3 | 2 | C→U | CCU→CUU | P→L |
| nad4L | 41 | 14 | 2 | C→U | UCU→UUU | S→F |
| nad4L | 47 | 16 | 2 | C→U | UCA→UUA | S→L |
| nad4L | 55 | 19 | 1 | C→U | CGG→UGG | R→W |
| nad4L | 86 | 29 | 2 | C→U | CCU→CUU | P→L |
| nad4L | 95 | 32 | 2 | C→U | UCA→UUA | S→L |
| nad4L | 100 | 34 | 1 | C→U | CCA→UCA | P→S |
| nad4L | 110 | 37 | 2 | C→U | UCA→UUA | S→L |
| nad4L | 131 | 44 | 2 | C→U | UCG→UUG | S→L |
| nad4L | 158 | 53 | 2 | C→U | UCG→UUG | S→L |
| nad4L | 179 | 60 | 2 | C→U | UCA→UUA | S→L |
| nad4L | 188 | 63 | 2 | C→U | UCA→UUA | S→L |
| nad4L | 197 | 66 | 2 | C→U | UCA→UUA | S→L |
| nad4L | 281 | 94 | 2 | C→U | UCU→UUU | S→F |
| ccmFn | 98 | 33 | 2 | C→U | CCU→CUU | P→L |
| ccmFn | 137 | 46 | 2 | C→U | UCG→UUG | S→L |
| ccmFn | 142 | 48 | 1 | C→U | CGU→UGU | R→C |
| ccmFn | 151 | 51 | 1 | C→U | CCU→UCU | P→S |
| ccmFn | 248 | 83 | 2 | C→U | UCA→UUA | S→L |
| ccmFn | 256 | 86 | 1 | C→U | CGG→UGG | R→W |
| ccmFn | 346 | 116 | 1 | C→U | CUU→UUU | L→F |
| ccmFn | 365 | 122 | 2 | C→U | UCG→UUG | S→L |
| ccmFn | 698 | 233 | 2 | C→U | CCU→CUU | P→L |
| ccmFn | 707 | 236 | 2 | C→U | UCA→UUA | S→L |
| ccmFn | 745 | 249 | 1 | C→U | CGU→UGU | R→C |
| ccmFn | 767 | 256 | 2 | C→U | UCA→UUA | S→L |
| ccmFn | 779 | 260 | 2 | C→U | CCA→CUA | P→L |
| ccmFn | 794 | 265 | 2 | C→U | UCA→UUA | S→L |
| ccmFn | 920 | 307 | 2 | C→U | GCC→GUC | A→V |
| ccmFn | 943 | 315 | 1 | C→U | CGC→UGC | R→C |
| ccmFn | 1261 | 421 | 1 | C→U | CGG→UGG | R→W |
| ccmFn | 1289 | 430 | 2 | C→U | CCA→CUA | P→L |
| ccmFn | 1306 | 436 | 1 | C→U | CAU→UAU | H→Y |
| ccmFn | 1321 | 441 | 1 | C→U | CGG→UGG | R→W |
| ccmFn | 1339 | 447 | 1 | C→U | CGG→UGG | R→W |
| ccmFn | 1372 | 458 | 1 | C→U | CGG→UGG | R→W |
| ccmFn | 1457 | 486 | 2 | C→U | CCA→CUA | P→L |
| ccmFn | 1469 | 490 | 2 | C→U | UCA→UUA | S→L |
| ccmFn | 1504 | 502 | 1 | C→U | CCC→UCC | P→S |
| cox1 | 761 | 254 | 2 | C→U | UCC→UUC | S→F |
| cox1 | 1186 | 396 | 1 | C→U | CAC→UAC | H→Y |
| cox1 | 1405 | 469 | 1 | C→U | CGU→UGU | R→C |
| cox1 | 1433 | 478 | 2 | C→U | UCA→UUA | S→L |
| cox1 | 1489 | 497 | 1 | C→U | CCA→UCA | P→S |
| cox1 | 242 | 81 | 2 | C→U | UCU→UUU | S→F |
| cox1 | 254 | 85 | 2 | C→U | UCU→UUU | S→F |
| cox1 | 452 | 151 | 2 | C→U | UCU→UUU | S→F |
| cox1 | 515 | 172 | 2 | C→U | UCC→UUC | S→F |
| cox1 | 551 | 184 | 2 | C→U | UCA→UUA | S→L |
| cox1 | 590 | 197 | 2 | C→U | CCA→CUA | P→L |
| cox1 | 715 | 239 | 1 | C→U | CGG→UGG | R→W |
| rps10 | 2 | 1 | 2 | C→U | ACG→AUG | T→M |
| rps10 | 43 | 15 | 1 | C→U | CCA→UCA | P→S |
| rps10 | 238 | 80 | 1 | C→U | CGG→UGG | R→W |
| rps10 | 302 | 101 | 2 | C→U | UCG→UUG | S→L |
| rps10 | 331 | 111 | 1 | C→U | CGA→UGA | R→* |
| rps10 | 56 | 19 | 2 | C→U | UCA→UUA | S→L |
| rps10 | 100 | 34 | 1 | C→U | CGU→UGU | R→C |
| rps10 | 287 | 96 | 2 | C→U | UCG→UUG | S→L |
| atp9 | 137 | 46 | 2 | C→U | UCU→UUU | S→F |
| atp9 | 212 | 71 | 2 | C→U | UCA→UUA | S→L |
| rps3 | 92 | 31 | 2 | C→U | UCA→UUA | S→L |
| rps3 | 512 | 171 | 2 | C→U | UCA→UUA | S→L |
| rps3 | 713 | 238 | 2 | C→U | UCG→UUG | S→L |
| rps3 | 865 | 289 | 1 | C→U | CGC→UGC | R→C |
| rps3 | 1364 | 455 | 2 | C→U | CCG→CUG | P→L |
| rps3 | 1502 | 501 | 2 | C→U | UCA→UUA | S→L |
| rps3 | 1555 | 519 | 1 | C→U | CGU→UGU | R→C |
| rps3 | 1588 | 530 | 1 | C→U | CCU→UCU | P→S |
| rps3 | 1555 | 519 | 1 | C→U | CGU→UGU | R→C |
| rps3 | 1588 | 530 | 1 | C→U | CCU→UCU | P→S |
| rpl16 | 104 | 35 | 2 | C→U | ACU→AUU | T→I |
| rpl16 | 407 | 136 | 2 | C→U | UCG→UUG | S→L |
| cox2 | 38 | 13 | 2 | C→U | CCU→CUU | P→L |
| cox2 | 71 | 24 | 2 | C→U | UCU→UUU | S→F |
| cox2 | 161 | 54 | 2 | C→U | UCG→UUG | S→L |
| cox2 | 443 | 148 | 2 | C→U | ACG→AUG | T→M |
| cox2 | 461 | 154 | 2 | C→U | CCA→CUA | P→L |
| cox2 | 476 | 159 | 2 | C→U | UCA→UUA | S→L |
| cox2 | 523 | 175 | 1 | C→U | CAU→UAU | H→Y |
| cox2 | 544 | 182 | 1 | C→U | CCU→UCU | P→S |
| cox2 | 557 | 186 | 2 | C→U | CCU→CUU | P→L |
| cox2 | 632 | 211 | 2 | C→U | UCG→UUG | S→L |
| cox2 | 721 | 241 | 1 | C→U | CCU→UCU | P→S |
| cox2 | 742 | 248 | 1 | C→U | CGG→UGG | R→W |
| nad5 | 242 | 81 | 2 | C→U | CCG→CUG | P→L |
| nad5 | 359 | 120 | 2 | C→U | UCU→UUU | S→F |
| nad5 | 374 | 125 | 2 | C→U | CCA→CUA | P→L |
| nad5 | 398 | 133 | 2 | C→U | UCU→UUU | S→F |
| nad5 | 539 | 180 | 2 | C→U | CCU→CUU | P→L |
| nad5 | 548 | 183 | 2 | C→U | UCG→UUG | S→L |
| nad5 | 608 | 203 | 2 | C→U | GCC→GUC | A→V |
| nad5 | 629 | 210 | 2 | C→U | UCU→UUU | S→F |
| nad5 | 676 | 226 | 1 | C→U | CUU→UUU | L→F |
| nad5 | 713 | 238 | 2 | C→U | UCG→UUG | S→L |
| nad5 | 725 | 242 | 2 | C→U | UCA→UUA | S→L |
| nad5 | 835 | 279 | 1 | C→U | CCG→UCG | P→S |
| nad5 | 1310 | 437 | 2 | C→U | UCA→UUA | S→L |
| nad5 | 1490 | 497 | 2 | C→U | CCC→CUC | P→L |
| nad5 | 1550 | 517 | 2 | C→U | ACC→AUC | T→I |
| nad5 | 1568 | 523 | 2 | C→U | CCG→CUG | P→L |
| nad5 | 41 | 14 | 2 | C→U | UCC→UUC | S→F |
| nad5 | 155 | 52 | 2 | C→U | CCG→CUG | P→L |
| nad5 | 1895 | 632 | 2 | C→U | UCA→UUA | S→L |
| nad5 | 1916 | 639 | 2 | C→U | UCU→UUU | S→F |
| nad5 | 1918 | 640 | 1 | C→U | CGU→UGU | R→C |
| nad5 | 1958 | 653 | 2 | C→U | UCG→UUG | S→L |
| nad4 | 608 | 203 | 2 | C→U | UCA→UUA | S→L |
| nad4 | 659 | 220 | 2 | C→U | UCU→UUU | S→F |
| nad4 | 836 | 279 | 2 | C→U | UCC→UUC | S→F |
| nad4 | 857 | 286 | 2 | C→U | CCA→CUA | P→L |
| nad4 | 887 | 296 | 2 | C→U | UCG→UUG | S→L |
| nad4 | 1010 | 337 | 2 | C→U | CCG→CUG | P→L |
| nad4 | 1016 | 339 | 2 | C→U | UCA→UUA | S→L |
| nad4 | 1109 | 370 | 2 | C→U | UCA→UUA | S→L |
| nad4 | 1129 | 377 | 1 | C→U | CUC→UUC | L→F |
| nad4 | 1132 | 378 | 1 | C→U | CCU→UCU | P→S |
| nad4 | 1151 | 384 | 2 | C→U | UCC→UUC | S→F |
| nad4 | 1172 | 391 | 2 | C→U | UCA→UUA | S→L |
| nad4 | 1307 | 436 | 2 | C→U | GCG→GUG | A→V |
| nad4 | 1355 | 452 | 2 | C→U | CCA→CUA | P→L |
| nad4 | 1373 | 458 | 2 | C→U | UCC→UUC | S→F |
| nad4 | 74 | 25 | 2 | C→U | ACU→AUU | T→I |
| nad4 | 77 | 26 | 2 | C→U | CCU→CUU | P→L |
| nad4 | 107 | 36 | 2 | C→U | CCG→CUG | P→L |
| nad4 | 158 | 53 | 2 | C→U | CCU→CUU | P→L |
| nad4 | 166 | 56 | 1 | C→U | CGG→UGG | R→W |
| nad4 | 197 | 66 | 2 | C→U | UCU→UUU | S→F |
| nad4 | 362 | 121 | 2 | C→U | ACA→AUA | T→I |
| nad4 | 368 | 123 | 2 | C→U | UCU→UUU | S→F |
| nad4 | 376 | 126 | 1 | C→U | CGU→UGU | R→C |
| nad4 | 416 | 139 | 2 | C→U | CCU→CUU | P→L |
| nad4 | 433 | 145 | 1 | C→U | CUU→UUU | L→F |
| nad4 | 436 | 146 | 1 | C→U | CCC→UUC | P→F |
| nad4 | 437 |  | 2 | C→U |  |  |
| nad4 | 449 | 150 | 2 | C→U | CCA→CUA | P→L |
| nad4 | 1417 | 473 | 1 | C→U | CAC→UAC | H→Y |
| nad4 | 1433 | 478 | 2 | C→U | CCG→CUG | P→L |
| sdh4 | 292 | 98 | 1 | C→U | CCC→UCC | P→S |
| atp8 | 47 | 16 | 2 | C→U | UCA→UUA | S→L |
| atp8 | 58 | 20 | 1 | C→U | CUC→UUC | L→F |
| cox3 | 245 | 82 | 2 | C→U | CCU→CUU | P→L |
| cox3 | 304 | 102 | 1 | C→U | CGG→UGG | R→W |
| cox3 | 311 | 104 | 2 | C→U | UCU→UUU | S→F |
| cox3 | 314 | 105 | 2 | C→U | UCU→UUU | S→F |
| cox3 | 419 | 140 | 2 | C→U | CCC→CUC | P→L |
| cox3 | 422 | 141 | 2 | C→U | CCU→CUU | P→L |
| cox3 | 566 | 189 | 2 | C→U | UCC→UUC | S→F |
| cox3 | 754 | 252 | 1 | C→U | CGG→UGG | R→W |
| cox3 | 764 | 255 | 2 | C→U | CCA→CUA | P→L |
| sdh4 | 67 | 23 | 1 | C→U | CCA→UCA | P→S |
| nad3 | 44 | 15 | 2 | C→U | UCG→UUG | S→L |
| nad3 | 62 | 21 | 2 | C→U | CCA→CUA | P→L |
| nad3 | 80 | 27 | 2 | C→U | CCA→CUA | P→L |
| nad3 | 124 | 42 | 1 | C→U | CAC→UAC | H→Y |
| nad3 | 146 | 49 | 2 | C→U | UCC→UUC | S→F |
| nad3 | 208 | 70 | 1 | C→U | CCU→UUU | P→F |
| nad3 | 209 |  | 2 | C→U |  |  |
| nad3 | 215 | 72 | 2 | C→U | CCG→CUG | P→L |
| nad3 | 230 | 77 | 2 | C→U | UCC→UUC | S→F |
| nad3 | 247 | 83 | 1 | C→U | CCU→UCU | P→S |
| nad3 | 251 | 84 | 2 | C→U | CCC→CUC | P→L |
| nad3 | 266 | 89 | 2 | C→U | CCC→CUC | P→L |
| nad3 | 275 | 92 | 2 | C→U | UCU→UUU | S→F |
| nad3 | 317 | 106 | 2 | C→U | UCU→UUU | S→F |
| nad3 | 344 | 115 | 2 | C→U | UCG→UUG | S→L |
| nad3 | 349 | 117 | 1 | C→U | CGG→UGG | R→W |
| ccmFc | 38 | 13 | 2 | C→U | UCC→UUC | S→F |
| ccmFc | 50 | 17 | 2 | C→U | CCU→CUU | P→L |
| ccmFc | 52 | 18 | 1 | C→U | CGU→UGU | R→C |
| ccmFc | 103 | 35 | 1 | C→U | CCC→UCC | P→S |
| ccmFc | 116 | 39 | 2 | C→U | CCU→CUU | P→L |
| ccmFc | 122 | 41 | 2 | C→U | UCC→UUC | S→F |
| ccmFc | 146 | 49 | 2 | C→U | CCU→CUU | P→L |
| ccmFc | 151 | 51 | 1 | C→U | CCU→UCU | P→S |
| ccmFc | 155 | 52 | 2 | C→U | UCA→UUA | S→L |
| ccmFc | 310 | 104 | 1 | C→U | CGU→UGU | R→C |
| ccmFc | 334 | 112 | 1 | C→U | CUU→UUU | L→F |
| ccmFc | 406 | 136 | 1 | C→U | CGU→UGU | R→C |
| ccmFc | 527 | 176 | 2 | C→U | CCC→CUC | P→L |
| ccmFc | 1234 | 412 | 1 | C→U | CGG→UGG | R→W |
| nad6 | 89 | 30 | 2 | C→U | CCC→CUC | P→L |
| nad6 | 169 | 57 | 1 | C→U | CAU→UAU | H→Y |
| nad6 | 191 | 64 | 2 | C→U | UCA→UUA | S→L |
| nad6 | 463 | 155 | 1 | C→U | CCU→UCU | P→S |
| rps4 | 50 | 17 | 2 | C→U | UCA→UUA | S→L |
| rps4 | 176 | 59 | 2 | C→U | UCA→UUA | S→L |
| rps4 | 205 | 69 | 1 | C→U | CAU→UAU | H→Y |
| rps4 | 278 | 93 | 2 | C→U | CCA→CUA | P→L |
| rps4 | 290 | 97 | 2 | C→U | UCG→UUG | S→L |
| rps4 | 302 | 101 | 2 | C→U | CCG→CUG | P→L |
| rps4 | 347 | 116 | 2 | C→U | CCG→CUG | P→L |
| rps4 | 446 | 149 | 2 | C→U | GCG→GUG | A→V |
| rps4 | 494 | 165 | 2 | C→U | UCA→UUA | S→L |
| rps4 | 917 | 306 | 2 | C→U | UCG→UUG | S→L |
| rps4 | 928 | 310 | 1 | C→U | CAU→UAU | H→Y |
| rps4 | 938 | 313 | 2 | C→U | CCA→CUA | P→L |
| rps4 | 953 | 318 | 2 | C→U | UCU→UUU | S→F |
| rps4 | 1004 | 335 | 2 | C→U | CCA→CUA | P→L |
| rps4 | 1018 | 340 | 1 | C→U | CGG→UGG | R→W |
| rps4 | 50 | 17 | 2 | C→U | UCA→UUA | S→L |
| rps4 | 176 | 59 | 2 | C→U | UCA→UUA | S→L |
| rps4 | 205 | 69 | 1 | C→U | CAU→UAU | H→Y |
| rps4 | 278 | 93 | 2 | C→U | CCA→CUA | P→L |
| rps4 | 290 | 97 | 2 | C→U | UCG→UUG | S→L |
| rps4 | 302 | 101 | 2 | C→U | CCG→CUG | P→L |
| rps4 | 347 | 116 | 2 | C→U | CCG→CUG | P→L |
| rps4 | 446 | 149 | 2 | C→U | GCG→GUG | A→V |
| rps4 | 494 | 165 | 2 | C→U | UCA→UUA | S→L |
| nad9 | 92 | 31 | 2 | C→U | UCU→UUU | S→F |
| nad9 | 113 | 38 | 2 | C→U | CCA→CUA | P→L |
| nad9 | 167 | 56 | 2 | C→U | UCG→UUG | S→L |
| nad9 | 239 | 80 | 2 | C→U | ACU→AUU | T→I |
| nad9 | 298 | 100 | 1 | C→U | CCG→UCG | P→S |
| nad9 | 328 | 110 | 1 | C→U | CGG→UGG | R→W |
| nad9 | 368 | 123 | 2 | C→U | UCC→UUC | S→F |
| nad9 | 398 | 133 | 2 | C→U | UCA→UUA | S→L |
| nad9 | 439 | 147 | 1 | C→U | CUU→UUU | L→F |
| ccmB | 28 | 10 | 1 | C→U | CAU→UAU | H→Y |
| ccmB | 80 | 27 | 2 | C→U | UCG→UUG | S→L |
| ccmB | 128 | 43 | 2 | C→U | UCA→UUA | S→L |
| ccmB | 137 | 46 | 2 | C→U | UCC→UUC | S→F |
| ccmB | 149 | 50 | 2 | C→U | CCG→CUG | P→L |
| ccmB | 154 | 52 | 1 | C→U | CGG→UGG | R→W |
| ccmB | 160 | 54 | 1 | C→U | CCU→UCU | P→S |
| ccmB | 164 | 55 | 2 | C→U | CCG→CUG | P→L |
| ccmB | 172 | 58 | 1 | C→U | CCU→UCU | P→S |
| ccmB | 179 | 60 | 2 | C→U | CCU→CUU | P→L |
| ccmB | 193 | 65 | 1 | C→U | CCU→UUU | P→F |
| ccmB | 194 |  | 2 | C→U |  |  |
| ccmB | 286 | 96 | 1 | C→U | CGG→UGG | R→W |
| ccmB | 304 | 102 | 1 | C→U | CGU→UGU | R→C |
| ccmB | 313 | 105 | 1 | C→U | CGU→UGU | R→C |
| ccmB | 338 | 113 | 2 | C→U | CCG→CUG | P→L |
| ccmB | 367 | 123 | 1 | C→U | CGG→UGG | R→W |
| ccmB | 380 | 127 | 2 | C→U | CCA→CUA | P→L |
| ccmB | 424 | 142 | 1 | C→U | CGU→UGU | R→C |
| ccmB | 428 | 143 | 2 | C→U | UCG→UUG | S→L |
| ccmB | 467 | 156 | 2 | C→U | UCG→UUG | S→L |
| ccmB | 476 | 159 | 2 | C→U | CCA→CUA | P→L |
| ccmB | 485 | 162 | 2 | C→U | UCA→UUA | S→L |
| ccmB | 494 | 165 | 2 | C→U | UCA→UUA | S→L |
| ccmB | 503 | 168 | 2 | C→U | CCA→CUA | P→L |
| ccmB | 512 | 171 | 2 | C→U | UCU→UUU | S→F |
| ccmB | 514 | 172 | 1 | C→U | CGU→UGU | R→C |
| ccmB | 551 | 184 | 2 | C→U | UCA→UUA | S→L |
| ccmB | 554 | 185 | 2 | C→U | UCG→UUG | S→L |
| ccmB | 566 | 189 | 2 | C→U | UCC→UUC | S→F |
| ccmB | 569 | 190 | 2 | C→U | UCU→UUU | S→F |
| ccmB | 572 | 191 | 2 | C→U | CCG→CUG | P→L |
| ccmB | 596 | 199 | 2 | C→U | UCG→UUG | S→L |
| ccmB | 611 | 204 | 2 | C→U | UCA→UUA | S→L |
| rpl10 | 344 | 115 | 2 | C→U | GCA→GUA | A→V |
| rpl10 | 445 | 149 | 1 | C→U | CGG→UGG | R→W |
| nad7 | 244 | 82 | 1 | C→U | CAU→UAU | H→Y |
| nad7 | 251 | 84 | 2 | C→U | UCA→UUA | S→L |
| nad7 | 316 | 106 | 1 | C→U | CGU→UGU | R→C |
| nad7 | 335 | 112 | 2 | C→U | UCA→UUA | S→L |
| nad7 | 344 | 115 | 2 | C→U | UCA→UUA | S→L |
| nad7 | 383 | 128 | 2 | C→U | UCA→UUA | S→L |
| nad7 | 578 | 193 | 2 | C→U | UCA→UUA | S→L |
| nad7 | 724 | 242 | 1 | C→U | CAU→UAU | H→Y |
| nad7 | 739 | 247 | 1 | C→U | CCU→UUU | P→F |
| nad7 | 740 |  | 2 | C→U |  |  |
| nad7 | 769 | 257 | 1 | C→U | CGC→UGC | R→C |
| nad7 | 944 | 315 | 2 | C→U | CCU→CUU | P→L |
| nad7 | 1057 | 353 | 1 | C→U | CGU→UGU | R→C |
| nad7 | 1103 | 368 | 2 | C→U | UCU→UUU | S→F |
| nad7 | 1124 | 375 | 2 | C→U | CCA→CUA | P→L |
| nad7 | 1166 | 389 | 2 | C→U | UCU→UUU | S→F |
| nad7 | 77 | 26 | 2 | C→U | UCA→UUA | S→L |
| nad7 | 137 | 46 | 2 | C→U | UCA→UUA | S→L |
| nad7 | 308 | 103 | 2 | C→U | GCU→GUU | A→V |
| nad7 | 140 | 47 | 2 | C→U | CCG→CUG | P→L |
| nad7 | 200 | 67 | 2 | C→U | UCU→UUU | S→F |
