# Supplementary material for: Organelle genome architecture of Salvia plebeia reveals mitochondrial recombination and evolutionary dynamics
Source: Front Plant Sci. 2026 Jul 9;17:1865234. doi: 10.3389/fpls.2026.1865234 (PMC13391575; doi:10.3389/fpls.2026.1865234)
Supplement: Supplementary file 11 [file Table11.docx]

**Table S11 | Summary of RNA-editing Events and Their Impact on Amino Acid Properties.**

| **Type** | **RNA-editing** | **Number** | **Percentage** |
| --- | --- | --- | --- |
| hydrophilic- hydrophilic | CAC(H)→UAC(Y) | 7 |  |
|  | CAU(H)→UAU(Y) | 16 |  |
|  | CGC(R)→UGC(C) | 6 |  |
|  | CGU(R)→UGU(C) | 22 |  |
|  | total | 51 | 12.59% |
| hydrophilic- hydrophobic | ACA(T)→AUA(I) | 5 |  |
|  | ACC(T)→AUC(I) | 1 |  |
|  | ACU(T)→AUU(I) | 5 |  |
|  | ACG(T)→AUG(M) | 3 |  |
|  | CGG(R)→UGG(W) | 29 |  |
|  | UCA(S)→UUA(L) | 66 |  |
|  | UCG(S)→UUG(L) | 34 |  |
|  | UCC(S)→UUC(F) | 23 |  |
|  | UCU(S)→UUU(F) | 32 |  |
|  | total | 198 | 48.89% |
| hydrophobic- hydrophilic | CCA(P)→UCA(S) | 4 |  |
|  | CCC(P)→UCC(S) | 5 |  |
|  | CCG(P)→UCG(S) | 3 |  |
|  | CCU(P)→UCU(S) | 19 |  |
|  | total | 31 | 7.65% |
| hydrophobic- hydrophobic | CCA(P)→CUA(L) | 30 |  |
|  | CCC(P)→CUC(L) | 11 |  |
|  | CCG(P)→CUG(L) | 28 |  |
|  | CCU(P)→CUU(L) | 21 |  |
|  | CCC(P)→UUC(F) | 2 |  |
|  | CCU(P)→UUU(F) | 6 |  |
|  | GCA(A)→GUA(V) | 2 |  |
|  | GCC(A)→GUC(V) | 2 |  |
|  | GCG(A)→GUG(V) | 5 |  |
|  | GCU(A)→GUU(V) | 3 |  |
|  | CUC(L)→UUC(F) | 5 |  |
|  | CUU(L)→UUU(F) | 9 |  |
|  | total | 124 | 30.61% |
| hydrophilic-stop | CGA(R)→UGA(*) | 1 |  |
|  | total | 1 | 2.47% |
|  | all | 405 | 100.00% |
