# Supplementary material for: Organelle genome architecture of Salvia plebeia reveals mitochondrial recombination and evolutionary dynamics
Source: Front Plant Sci. 2026 Jul 9;17:1865234. doi: 10.3389/fpls.2026.1865234 (PMC13391575; doi:10.3389/fpls.2026.1865234)
Supplement: Supplementary file 12 [file Table12.docx]

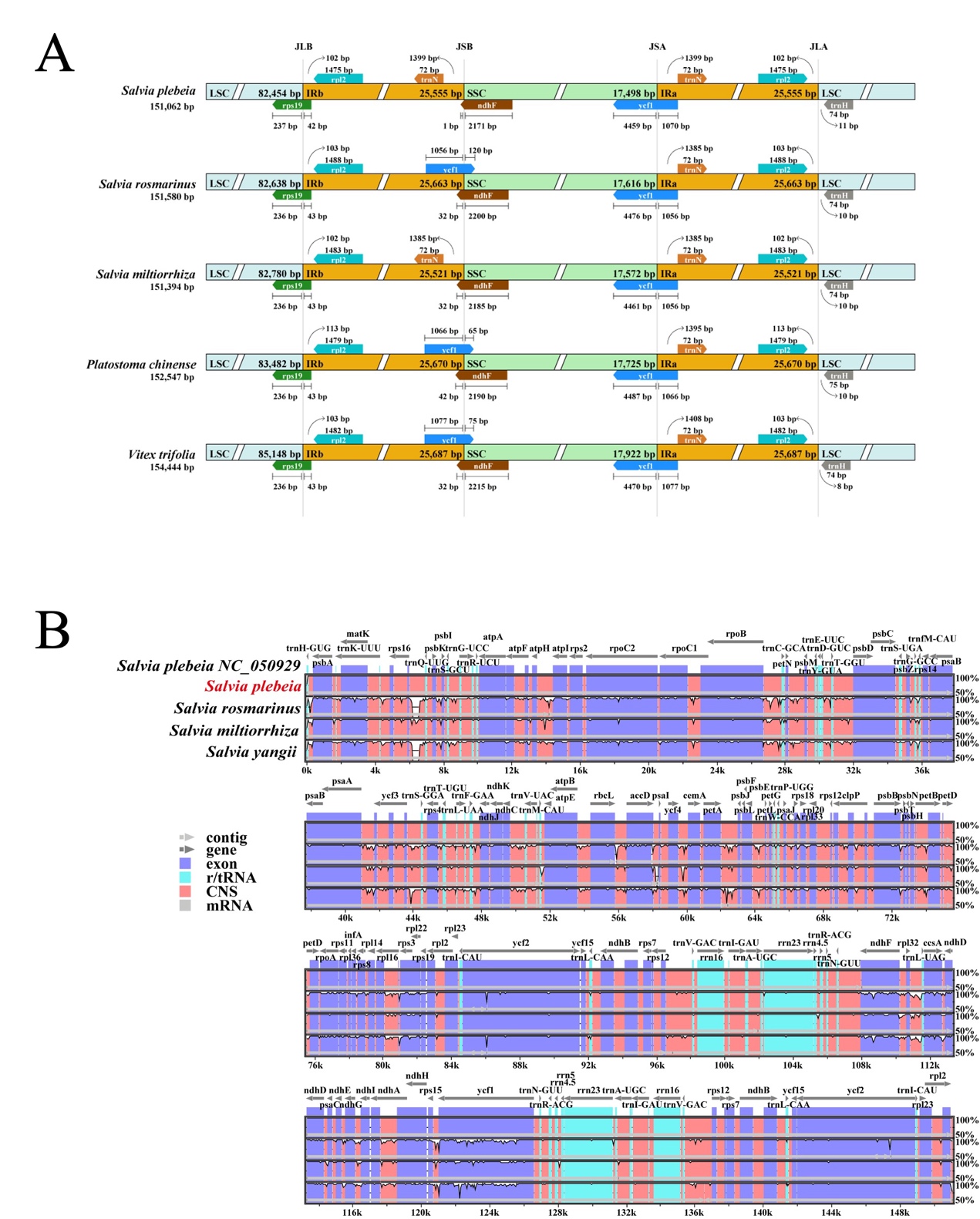
**Figure S1 | Structural and comparative analysis of *S. plebeia* cpgenome and MTPT integration.** (A) Comparative analysis of LSC, SSC, and IR boundaries among cpgenome from four Lamiaceae species, demonstrating differences in genomic structure and boundary positioning across the family. (B) Comparative analysis of genome structure among cpgenome from five Salvia species using mVISTA.
