# Supplementary material for: Organelle genome architecture of Salvia plebeia reveals mitochondrial recombination and evolutionary dynamics
Source: Front Plant Sci. 2026 Jul 9;17:1865234. doi: 10.3389/fpls.2026.1865234 (PMC13391575; doi:10.3389/fpls.2026.1865234)

**Figure S2 | Dot plots comparing the chloroplast sequence of *S. plebeia* against four Salvia plants*.* (**A-D) Dot plots of *S. plebeia* againest *Salvia plebeia NC_050929, Salvia rosmarinus, Salvia miltiorrhiza* and *Salvia yangii*.


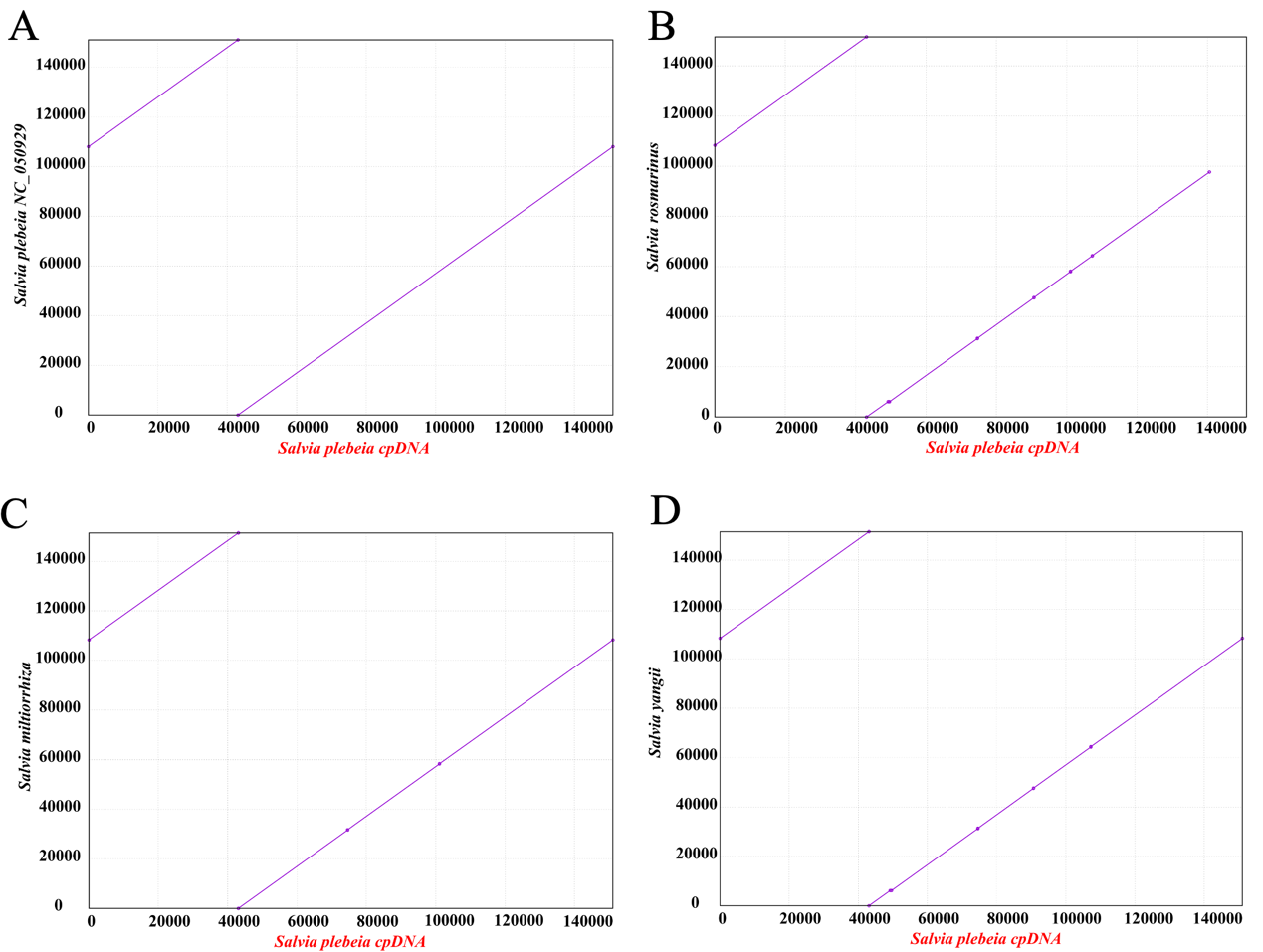

Supplement: Supplementary file 13 [file Table13.docx]
