# Supplementary material for: Organelle genome architecture of Salvia plebeia reveals mitochondrial recombination and evolutionary dynamics
Source: Front Plant Sci. 2026 Jul 9;17:1865234. doi: 10.3389/fpls.2026.1865234 (PMC13391575; doi:10.3389/fpls.2026.1865234)
Supplement: Supplementary file 14 [file Table14.docx]

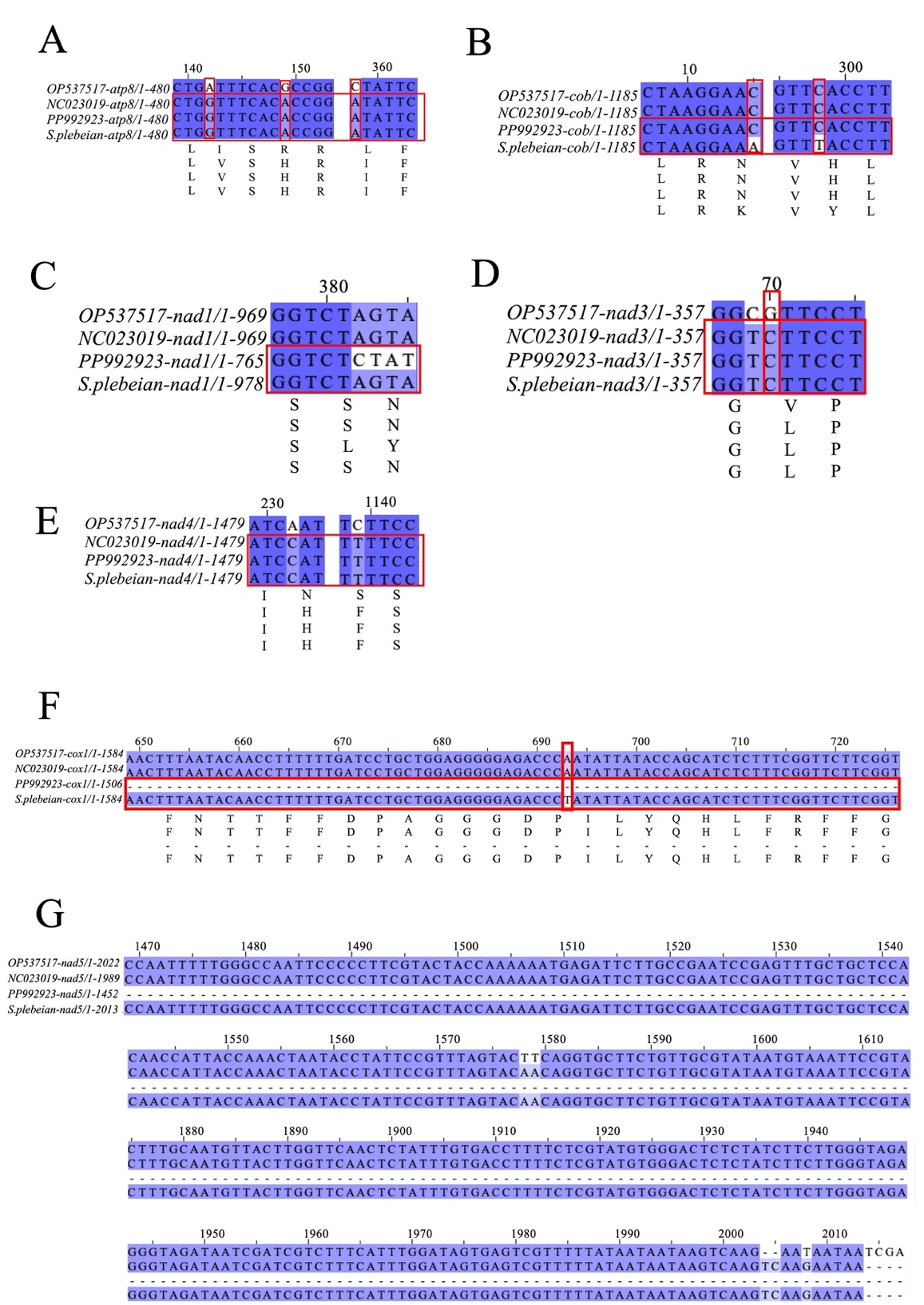
**Figure S3 | Alignments of the nucleotide and amino acid sequences of PCGs from four mitogenomes.** Multiple sequence alignments were conducted using MAFFT software with default parameters. Panels (A-G) correspond to the alignment results of the *atp8*, *cob*, *nad1*, *nad3*, *nad4*, *cox1*, and *nad5* genes, respectively. The mitogenomes represented by PP9929923, NC023019 and OP537517 correspond to *S. rosmainus*, *S. miltiorrhiz*a and *P. chinense*.
